# Implementing an Operational Framework to Develop a Streamflow Duration Assessment Method: A Case Study from the Arid West United States

**DOI:** 10.3390/w13223310

**Published:** 2021-11-22

**Authors:** Raphael D. Mazor, Brian J. Topping, Tracie-Lynn Nadeau, Ken M. Fritz, Julia E. Kelso, Rachel A. Harrington, Whitney S. Beck, Kenneth S. McCune, Aaron O. Allen, Robert Leidy, James T. Robb, Gabrielle C. L. David

**Affiliations:** 1 Southern California Coastal Water Research Project, Costa Mesa, CA 92626, USA; 2 Office of Wetlands, Oceans, and Watersheds, U.S. Environmental Protection Agency, Washington, DC 20460, USA; 3 Region 10, U.S. Environmental Protection Agency, Portland, OR 97205, USA; 4 Center for Environmental Measurement and Modeling, Office of Research and Development, U.S. Environmental Protection Agency, Cincinnati, OH 45268, USA; 5 Oak Ridge Institute of Science and Education (ORISE) Fellow, Oak Ridge, TN 37831, USA; 6 Region 8, U.S. Environmental Protection Agency, Denver, CO 80202, USA; 7 Los Angeles District, U.S. Army Corps of Engineers, Los Angeles, CA 90017, USA; 8 Region 9, U.S. Environmental Protection Agency, San Francisco, CA 94105, USA; 9 Sacramento District, U.S. Army Corps of Engineers, Sacramento, CA 95814, USA; 10 Engineer Research and Development Center Cold Regions Research and Engineering Laboratory U.S. Army Corps of Engineers, Hanover, NH 03755, USA

**Keywords:** classification, flow duration, streams, perennial, intermittent, ephemeral, temporary, flow permanence, intermittency, rapid assessment

## Abstract

Streamflow duration information underpins many management decisions. However, hydrologic data are rarely available where needed. Rapid streamflow duration assessment methods (SDAMs) classify reaches based on indicators that are measured in a single brief visit. We evaluated a proposed framework for developing SDAMs to develop an SDAM for the Arid West United States that can classify reaches as perennial, intermittent, or ephemeral. We identified 41 candidate biological, geomorphological, and hydrological indicators of streamflow duration in a literature review, evaluated them for a number of desirable criteria (e.g., defensibility and consistency), and measured 21 of them at 89 reaches with known flow durations. We selected metrics for the SDAM based on their ability to discriminate among flow duration classes in analyses of variance, as well as their importance in a random forest model to predict streamflow duration. This approach resulted in a “beta” SDAM that uses five biological indicators. It could discriminate between ephemeral and non-ephemeral reaches with 81% accuracy, but only 56% accuracy when distinguishing 3 classes. A final method will be developed following expanded data collection. This Arid West study demonstrates the effectiveness of our approach and paves the way for more efficient development of scientifically informed SDAMs.

## 1. Introduction

Streamflow duration drives biodiversity patterns and ecosystem functions in stream reaches and their adjacent riparian zones, and knowledge of a reach’s streamflow duration may be used to inform a wide range of management decisions, such as determining applicable water quality standards or setting goals for restoration efforts [1]. However, accurate characterization of streamflow duration requires long-term hydrologic data, which is typically only available at a relatively small number of gaged reaches [2,3]. Hydrologic models have been developed to predict streamflow duration (e.g., [4-8]). However, hydrologic models typically lack the ability to distinguish between ephemeral and intermittent reaches (due to the scarcity of gaged ephemeral reaches), and models based on gages with undisturbed catchments may not apply to reaches with altered hydrology [1]. Even without these limitations, managers would still need empirical methods to validate predictions from hydrologic models at ungagged reaches. Consequently, resource managers and researchers need rapid methods to assess streamflow duration at the reach scale where long-term data are unavailable; streamflow duration assessment methods (SDAMs) meet this need.

The term “streamflow duration” describes the extent to which a stream reach has continuous surface flow over time, typically on an annual time-scale. Perennial stream reaches have uninterrupted surface flow throughout the year, except in years with extreme drought conditions; non-perennial streams cease to flow for some period of time [1,9]. The period that non-perennial streams cease to flow varies greatly depending on climate, geology and land cover; classification of streamflow duration can also depend on the spatial and temporal scale being evaluated [10]. Stream drying periods exist along a continuum, and it remains unknown how diverse or predictable non-perennial flow regimes are from one another within and among geographic regions [8]. Nevertheless, distinct flow classes are often used to describe variability in non-perennial flow regimes based on flow metrics and watershed characteristics. For example, flow metrics (e.g., mean and variability in zero-flow, low flows, and high flows) were used to classify flow regimes of stream reaches into seven non-perennial archetypes in the Arid West U.S. [8] and three non-perennial archetypes in the Ozark-Ouachita highlands [11]. Resource managers and regulators typically require clearly defined flow classifications that can be determined based on widely available and easily interpretable data or field indicators. For example, the U.S. Texas Commission on Environmental Quality simply distinguishes between intermittent streams with and without perennial pools [12].

Streamflow duration is a primary component of stream hydrological regimes, along with the magnitude, frequency, rate of change, and timing of flow or drying events. It is a fundamental driver of stream ecosystem dynamics, life-history strategies, and diversity patterns (e.g., [13-19]). Streamflow duration affects fluvial processes, such as the delivery of nutrients, energy and other materials, and flux with terrestrial systems [20]. Stream networks in arid regions are characterized by a high degree of spatial and temporal heterogeneity [21], and transitions between flowing, pooled, and dry phases lead to greater temporal variation in in-stream environmental conditions in non-perennial streams compared to perennial streams [22]. Correspondingly, the biodiversity and ecosystem functions supported by different reaches within a watershed vary, depending on their duration of flow [19,22-24].

There are multiple implications of streamflow duration for water resource management. Whether a stream is perennial or non-perennial informs implementation of state and local mandates and ordinances, such as riparian buffer requirements. Knowledge of stream-flow class improves predictability for ecological assessment of streams to set appropriate water quality expectations, predict the provision of ecosystem services, and may inform the prioritization of restoration and protection efforts. Additionally, this information could help determine whether a stream may be subject to jurisdiction under the U.S. Clean Water Act, which encompasses several regulatory and non-regulatory programs affecting the management of water resources.

**Figure 1.**
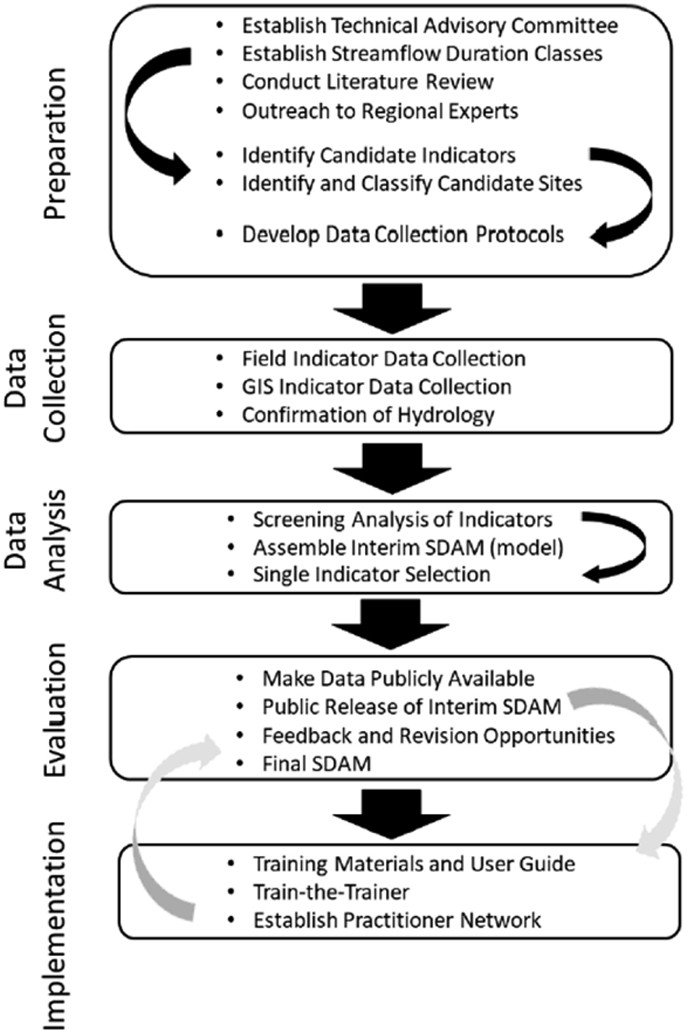
Operational framework for SDAM development. Small black arrows indicate stepwise actions within a process step, although some actions occur simultaneously and may be repeated throughout the project (e.g., outreach to local experts). The gray arrows denote that implementation actions are iterative, ideally supporting public release of an interim beta SDAM and then a final SDAM. Reproduced from Fritz and others [1].

Given the importance of streamflow duration to management decisions, there is a need for rapid streamflow duration assessment methods (SDAMs) to evaluate reaches where long-term hydrologic data are lacking. Fritz and others [1] proposed an approach to developing SDAMs, based in part on experiences in developing SDAMs for the Pacific Northwest [25]. Fritz and others [1] present a conceptual framework that outlines a process to integrate three components of an SDAM development study (hydrological data, indicators, and study reaches) into a data-driven method. They also present an operational framework detailing five process steps needed for SDAM development: preparation, data collection, data analysis, evaluation, and implementation (Figure 1). End-users are engaged through each step of the SDAM development process to ensure that the final method can be used consistently and accurately in the field. Our effort to develop an SDAM for the Arid West (AW) represents a case study to evaluate this approach. This effort culminated in a beta method jointly released by the U.S. Environmental Protection Agency (USEPA) and U.S. Army Corps of Engineers (Corps) for use in a one-year interim period during which additional data collection and public comment will occur [26]. Our objectives in this paper are to document the development of the beta SDAM for the AW (SDAM AW), evaluate its performance compared to other SDAMs used in portions of the region, and identify the successes and challenges that we experienced when following the approach of Fritz and others [1].

## 2. Materials and Methods

We followed the process outlined by Fritz and others [1] in developing this SDAM.

### 2.1. Streamflow Duration Classes

Streamflow duration classifications are applied to a reach where streamflow duration information is needed. A reach is a section of stream or river along which similar hydrologic conditions exist (e.g., discharge, depth, velocity, or sediment transport dynamics) and consistent drivers of hydrology are evident (e.g., slope, substrate, geomorphology, or confinement). A channel is an area that is confined by banks and a bed and contains flowing water (continuously or not). Our definitions of streamflow duration classes followed those used by Nadeau [27]:

- Ephemeral reaches flow only in direct response to precipitation. Water typically flows only during and/or shortly after large precipitation events, the streambed is always above the water table, and stormwater runoff is the primary water source.
- Intermittent reaches contain sustained flowing water for only part of the year, typically during the wet season, where the streambed may be below the water table or where the snowmelt from surrounding uplands provides sustained flow. The flow may vary greatly with stormwater runoff.
- Perennial reaches contain flowing water continuously during a year of normal rainfall, often with the streambed located below the water table for most of the year. Groundwater typically supplies the baseflow for perennial reaches, but the baseflow may also be supplemented by stormwater runoff or snowmelt.

### 2.2. Study Area

**Figure 2.**
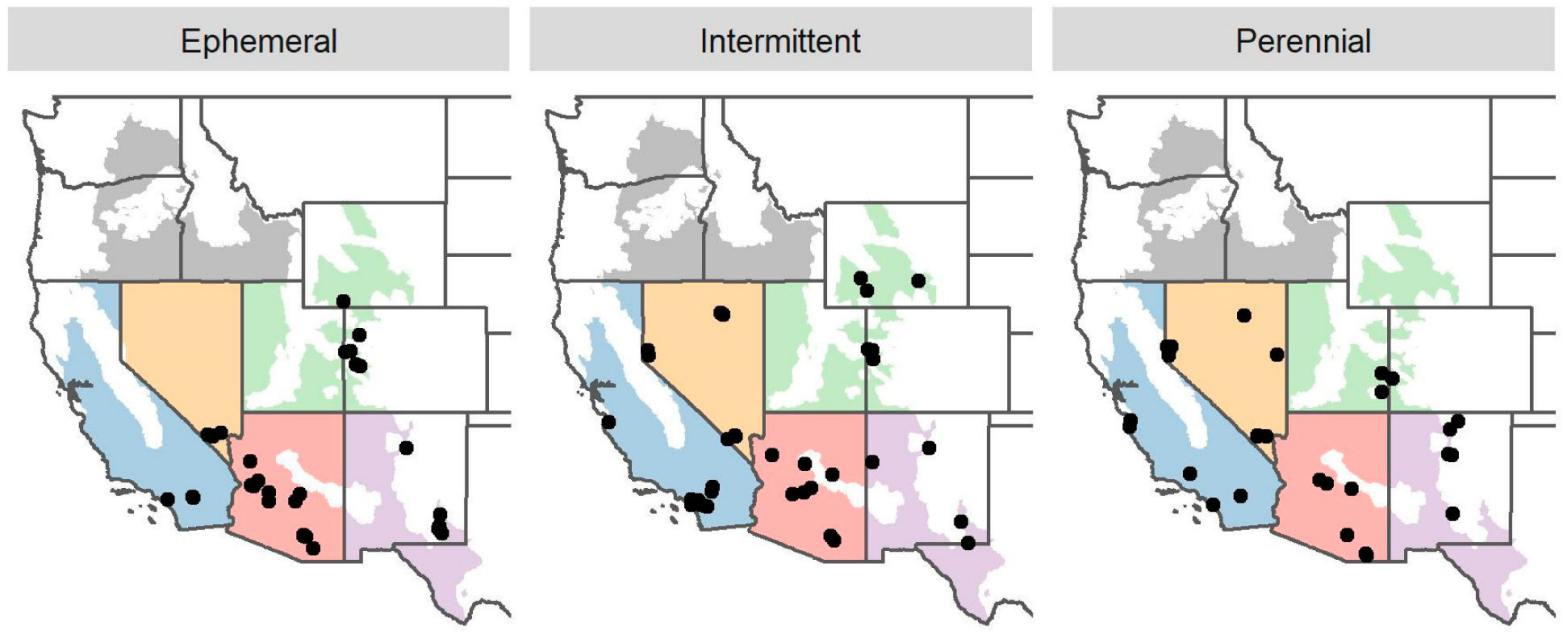
Study reaches in the AW. Colors indicate the 5 strata used for ensuring geographic representativeness of the dataset (blue: California; orange: Nevada; red: Arizona, purple: New Mexico and Texas; and green: Utah, Colorado, Wyoming, and a small portion of Montana). The gray area indicates arid portions of the Pacific Northwest, which are covered by the method of Nadeau [27] and was excluded from the present study. Black dots indicate sampling reaches.

The AW encompasses over 1.4 million km^2^ in the western United States, covering portions of states from California to Texas and Montana. The region is defined by a combination of variables related to climatic, landcover, vegetation, and soil conditions (Figure 2) [28]. The AW includes deserts, grasslands, steppes, shrublands, and woodlands, and is characterized by relatively low rainfall (i.e., <15 inches per year) [28]. In the Mediterranean climate of coastal California, rainfall is restricted to mild and wet winter months; many stream reaches cease to flow in the dry summer [29]. In contrast, other portions of the AW are characterized by desert climates, where rainfall is less predictable. The Sonoran Desert is characterized by both a wet and mild winter, as well as summer monsoons, while the Great Basin, Mojave, and Chihuahuan Deserts regularly experience freezing winters. Snowmelt has minimal influence on stream hydrology in the Arid West, except in high-elevation areas [28]. Hydrologic models suggest that the vast majority of stream-miles in the region are nonperennial [30,31], although the relative extent of ephemeral and intermittent reaches is not well documented. Non-perennial streams dominate the region [21], and (in contrast to other regions) often occur in the middle or lower portions of watersheds in addition to headwaters [28].

Many of the largest metropolitan areas in the USA are located within the AW, and the region continues to experience rapid population growth with expanding urbanization (e.g., [32,33]). Thus, the need for an SDAM in permitting and management programs is high in this region. Within the AW, at least two SDAMs are currently in use, applicable to only specific geographic areas: the Pacific Northwest (PNW) method [27], and the New Mexico (NM) method [34]. However, prior to the current study, the rest of the region lacked any tool to assess streamflow duration. Our effort focused on the portion of the AW outside the PNW (Figure 2).

### 2.3. Preparation

#### 2.3.1. Establish an Advisory Committee

Fritz and others [1] recommend establishing a technical advisory committee comprised of scientific staff from state, tribal, federal, and local agencies involved in water resources management likely to use the SDAM. At the outset of the project, we assembled a regional steering committee (RSC) consisting of technical staff at Corps Districts and USEPA Regional Offices in the AW region that manage programs where streamflow duration information is often needed (e.g., U.S. Clean Water Act programs, including permits and enforcement). RSC members were selected based on their expertise in both scientific and programmatic elements relevant to streamflow duration classification needs. The RSC served several functions in the development process, such as reviewing technical products, facilitating connections with local experts, and identifying resources such as sources of hydrologic data.

#### 2.3.2. Identify Candidate Indicators

We identified potential indicators that were supported by the scientific literature (reviewed in [35]) or used in existing SDAMs developed for portions of the AW; specifically, the New Mexico SDAM (NM method) [34], and the SDAM for the PNW (PNW method) [27]. Following input from the RSC, these candidate indicators were then screened using the criteria described by Fritz and others [1], including:

- Consistency: Does the indicator consistently discriminate among flow duration classes (e.g., demonstrated in multiple studies)?
- Repeatability: Can different practitioners take similar measurements, given sufficient training and standardization?
- Defensibility: Does the indicator have a rational mechanistic relationship with flow duration, as either a response or a driver?
- Rapidness: Can the indicator be measured during a one-day reach-visit (even if subsequent lab analyses are required)?
- Objectivity: Does the indicator rely on objective (often quantitative) measures, as opposed to subjective judgments of practitioners?
- Robustness: Does human activity complicate indicator measurement or interpretation (e.g., poor water quality may affect the expression of some biological indicators)?
- Practicality: Can practitioners realistically sample the indicator with typical capacity, skills, and resources?

Indicators were included in this study (Table 1) if they met all of the above criteria or were included in the NM or PNW SDAMs to facilitate comparison among the methods [35]. At a typical site, a field crew consisting of two people could measure all indicators and complete an assessment in about an hour (not including time to identify aquatic invertebrates).

#### 2.3.3. Identify candidate reaches

##### Goals in Selecting Reaches for Method Development

We had two objectives in selecting candidate reaches for the AW region covered by this study: first, to include a sufficient number of reaches in each streamflow duration class to characterize variability in indicator measurements; second, to select reaches representing the range of key natural and disturbance gradients within the region to ensure that the method would work in all conditions where assessments were needed. To support our goal of geographic representativeness, we divided the AW into five subregional strata: one stratum each for California, Arizona, and Nevada; a stratum combining New Mexico and Texas; and a stratum comprising the remaining states (i.e., Colorado, Wyoming, Utah, and Montana; Figure 2). We aimed to select 100 publicly accessible stream reaches (one assessed location per reach) with equal representation of perennial, intermittent, and ephemeral flow duration among and within the five AW subregions.

##### Classifying Streamflow Duration Based on Hydrologic Data

In order to determine the accuracy of our candidate indicators and the resulting beta method, it was necessary to assign each study reach an independently determined flow classification based on direct observations of stream hydrology (e.g., flow versus no flow), independent of indirect indicators of stream flow duration observed remotely or in the field (e.g., watershed size, hydrophytic vegetation, and channel structure). To screen reaches for use in method development, we first compiled a list of candidate study reaches based on existing hydrologic data records (e.g., USGS stream gages, water presence logger, wildlife cameras, and field photos), published studies, and interviews with local experts familiar with the specific reach’s hydrology. Continuous data records (e.g., daily flow loggers and wildlife cameras) were used to classify a reach as perennial if they indicated fewer than 5% zero-flow days, ephemeral if they indicated fewer than 5% flowing days, or intermittent if they indicated an intermediate number of flowing days over the period of record (which varied from a single year to many decades, depending on the data source). These criteria have been used in previous studies (e.g., [40]), and they serve to reduce the influence of extreme climatic events or rare instrumentation failure that could otherwise modify a classification [3]. Discontinuous data (e.g., field notes, field photos) were used to confirm reach flow duration if direct observations of stream hydrology indicated flowing and dry conditions at appropriate times (e.g., seasonal wet or dry periods), or to supplement flow classifications based on multiple data sources. Details about reach classification and correction of misclassified reaches are presented in Supplementary File S1a.

Classified reaches were prioritized for study inclusion based on the number and type of data sources available to determine actual streamflow duration classification. Reaches where flow duration could be determined based on multiple data sources (e.g., water presence loggers and expert knowledge) were categorized as “preferred” for study inclusion. Reaches where flow classes were determined based solely on interpretation of USGS stream gage data without consultation of a local expert were categorized as “USGS gage” reaches. Reaches identified through local expertise alone were categorized as “acceptable” and included in this study where necessary to fill data gaps in study subregions where a sufficient number of “preferred” and “USGS gage” reaches with an intermittent or ephemeral flow classification could not be identified.

##### Selecting Reaches for Inclusion in This Study

Once a list of classified candidate study reaches of known streamflow duration was generated as previously described, we first identified ephemeral reaches (that is, the most limited classification among our candidates due to the paucity of hydrologic data on ephemeral reaches) in each of the five subregions. We then added intermittent and perennial reaches in close proximity to create “clusters” of multiple reaches following the design of Nadeau and others [25] to maximize the number of study reaches that could be sampled by limiting travel time and access issues. Whenever possible, “preferred” reaches were selected before “acceptable” reaches. Backup reaches were identified for every cluster in case a reach was inaccessible. All selected study reaches were on publicly accessible property and within a 20 min walk of an access point. Most of these reaches were visited only once (typically in summer); ten percent of reaches were targeted for an additional visit under different seasons to provide information about temporal variability of the indicators and consistency of the final method. This process resulted in a list of 100 target coordinates representing the downstream end of potential study reaches.

#### 2.3.4. Focus-Area Studies

In addition to the reaches described above, we included additional reaches of interest to local water resource managers in two watersheds: the Santa Margarita River in California and the Hassayampa River in Arizona. Reaches in these watersheds served as focus-area studies. Each focus-area study was led by practitioners with different backgrounds, but each frequently need streamflow duration information as part of their job duties. Thus, they could provide the method development team with early independent feedback of how well the method is likely to suit their needs. Each practitioner was provided with a day of training in the same protocols described below, after which they collected data from each reach during multiple repeated visits throughout the year. Apart from the greater frequency of sampling, data collection procedures at focus-area reaches were identical to procedures conducted at other reaches in this study.

Reaches within each focus area were located along a longitudinal gradient from headwaters to mainstems, without prior knowledge of flow duration. Stream Temperature, Intermittence and Conductivity loggers (STIC loggers, [41]) were installed at each focus-area study reach to enable their eventual classification. The goal in selecting these reaches was to enable study leads to test methods in a real-world application and provide feedback on challenges that became evident in their experience. Additionally, data generated from these focus-area study reaches were also included in method development at reaches where the true streamflow duration could be determined following the same approach used to determine true streamflow duration classes at other study reaches.

### 2.4. Data Collection

To guide data collection, we developed a protocol that described measurement of indicators identified in the literature review, or were part of SDAMs used within the region. Specifically, we included “Level 1” indicators of the NM method [34], and all indicators of the PNW method [27]. Indicators are summarized in Table 1, and the complete protocol provided in Supplementary File S1. A study reach was established by first approaching the target coordinates and measuring bankfull width at three locations (at the target location, 15 m upstream, and 30 m upstream). The total study reach length was then defined as 40 times the average width, but no longer than 200 m and no shorter than 40 m. If necessary, the study reach boundaries were adjusted to exclude major tributaries, improve access, or to maintain consistency with channel features that could affect streamflow duration (e.g., valley confinement, streambed substrate, proximity to a culvert or road crossing). Details about quality assurance are provided in the Quality Assurance Project Plan (Supplementary File S1).

Measured indicators were grouped into several types, briefly described as follows:

#### 2.4.1. Geomorphic Indicators

Valley slope was measured with a handheld clinometer, and bankfull width was measured with measuring tape or a stadia rod. Several indicators were measured based on visual estimation following the scoring guidance in the NM method, including sinuosity, floodplain and channel dimensions (i.e., the entrenchment ratio), in-channel structure/riffle-pool sequence, the extent of deposition sediment on plants and debris on the floodplain, and the extent of substrate sorting.

#### 2.4.2. Hydrologic Indicators

The extent of surface and subsurface flow, as well as the number of isolated pools was visually estimated following the PNW method [27]. The extent of water in the channel was scored following the guidance in the NM method [34]. The presence of seeps or springs within one-half channel width of the channel was noted. The presence of hydric soils was evaluated by digging in the top 6 inches of substrate at the toe of the banks in up to 3 locations. In channels without surface water, soil moisture and texture were measured at three locations. The number of woody jams within the reach or up to 10 m outside the reach was noted. For our purposes, a woody jam must contain at least 3 large pieces of wood (>1 m long and >10 cm in diameter), span the entire width of the channel, and be in contact with the streambed such that it would disrupt the movement of water or sediment during flowing conditions.

For hydrologic indicators, we distinguished between those reflecting direct measures of water presence (e.g., percent of the reach with surface flow and soil moisture) from indirect measures (e.g., hydric soils and number of woody jams). Doing so allowed us to compare models with or without these types of indicators.

#### 2.4.3. Biological Indicators

The abundance of selected biological indicators was scored following the guidance in the NM method: fish, amphibians, aquatic invertebrates (referred to as benthic macroinvertebrates in the NM method [34]), and filamentous algae. Other indicators derived from the NM method requiring subjective scoring included differences in vegetation between the riparian corridor and adjacent uplands, and the absence of upland rooted plants in the streambed. The presence of iron-oxidizing fungi or bacteria were also noted.

Aquatic invertebrates were collected for up to 15 min from at least 6 locations representing the range of microhabitats available in an assessment reach (e.g., riffles, pools, undercut banks). In dry streams, suitable microhabitats (e.g., remnant pools, under large cobbles, and stream margins) were searched for shells, cases, exuviae, and other evidence of aquatic invertebrates. Specimens were identified to the best practical level in the field (generally family), and vouchers of every taxon encountered were retained and sent to a lab to verify identifications. Up to 10 individuals per morphotaxon were counted and recorded. The presence of taxa designated as indicators of perennial flow by Nadeau [27] were noted.

Hydrophytes (i.e., those with Facultative-Wet [FACW] or Obligate [OBL] status in the AW Regional Wetland Plant List from the US Army Corps of Engineers by Lichvar and others [36]) growing within the channel or within a half-channel-width of the channel were noted, regardless of prevalence or dominance. Taxa not included by Lichvar and others [36], such as *Populus freemontii*, were not considered to be hydrophytes. Where necessary, photo vouchers or specimens were collected to verify identifications.

Observations of live fish or aquatic life stages of snakes and amphibians were noted. Non-native mosquitofish (*Gambusia* sp., typically *G. affinis*) were noted separately.

Streambed cover by live or dead algal mats, liverworts, or mosses with “streamer” morphology were also estimated.

#### 2.4.4. Geospatial Data

Geospatial data were collected in order to evaluate potential indicators and as co-variates in models. Level 2 and Level 3 Omernik Ecoregions were derived by overlaying points on shapefiles downloaded from the EPA [39]. Climate data derived from PRISM were assessed for each sampling location using the *PRISM* package [38]. A large number of landscape-scale metrics relating to watershed characteristics (e.g., soil type, geology) were acquired from the StreamCat dataset [37] by first determining the unique identifier of the nearest stream segment in the National Hydrography Dataset Plus (NHD Plus, [42]); however, a handful of study reaches were located on stream segments that are not represented in the NHD Plus dataset, and therefore had no data available in StreamCat.

### 2.5. Data Analysis

All data and code used in analyses is provided in Supplementary File S1.

#### 2.5.1. Calculation of Metrics

Data from indicator measurements were converted into metrics that could be used in an SDAM. For example, “number of mayflies” is a metric derived from the aquatic invertebrate indicator data. Whenever possible, metrics were expressed in continuous or ordinal formats, although binary metrics (e.g., “presence of hydrophytic plants”) were also considered. This process resulted in 54 metrics derived from field-collected data (7 geomorphic metrics, 7 hydrological metrics, 40 biological metrics), and 101 metrics derived from geospatial data, of which 83 were derived from StreamCat. Most biological metrics for aquatic invertebrates were expressed as richness or abundance, both relativized to sample totals and in unrelativized (raw) forms; because our collection methods may have undercounted non-insects in streams where insects dominated, metrics that focused on non-insects (e.g., Gastropoda, Oligochaeta, and Diptera [GOLD] taxa) were only evaluated in relativized forms. The full list of analyzed metrics are presented in Supplementary File S3.

#### 2.5.2. Metric Screening

As an initial data exploration step, we visualized the relationships between streamflow duration class and indicators by ordinating all 155 metrics for all samples in the dataset in a nonmetric multidimensional scaling using Gowers’ distance. Convex hulls were drawn around each streamflow duration class to help visualize their distributions in ordination space. The 2-axis ordination was computed using the metaMDS function in the *vegan* R package [43]. Correlation coefficients (Spearman’s rho) were calculated between ordination axes and metric values.

Metrics were evaluated against a number of criteria to determine their suitability for inclusion in an SDAM (Table 2). We developed criteria following approaches for screening metrics in bioassessment indices (e.g., [44]), and applied them to data from initial reach-visits (i.e., data from revisits were withheld from analysis). One criterion was a distribution statistic, calculated as percent dominance of the most common value (which was typically zero); all metrics had to meet this criterion. The remaining criteria were based on measures of responsiveness. Most of these measures were based on statistical comparisons of mean values at different subsets of reaches (e.g., t-statistic from a comparison of metric values at perennial and non-perennial reaches), as has been used in other studies [45-47]. Another responsiveness statistic was based on variable importance (specifically, mean decrease in accuracy) from a random forest model to predict streamflow duration class from all possible metrics; the model was calibrated using the default option from the randomForest function in the *randomForest* package in R [48]. Metrics had to meet at least one responsiveness criterion to be considered in further analyses.

#### 2.5.3. Metric Selection

Once a limited number of candidate metrics could be identified by the screening process, we used the recursive feature elimination (rfe) function in the R *caret* package [49] to select a final set of metrics for the beta SDAM based on their importance in random forest models. Briefly, rfe is a form of stepwise selection where complex (i.e., those based on many metrics) are calibrated, and simpler models are considered by calibrating new models after eliminating the least important metrics. We considered the most complex model (i.e., all candidate metrics included), eliminating 5 variables at a time in each step based on low variable importance until a 20-variable model was identified; after this point, only one variable was eliminated in each step. The best performing model (highest accuracy in predicting streamflow duration class, as measured by Cohen’s Kappa) was identified, and the simplest model (i.e., the one with the fewest variables) with a Kappa statistic within 1% of the best was selected to identify the final set of metrics. If the best-performing model selected by this approach had more than 20 variables, the 20-variable model was selected.

We applied this modeling process to different subsets of the dataset, including:

- The full region-wide dataset, and
- Five separate datasets, one for each subregion shown in Figure 2. For both of these two options, the modeling process was implemented:
- With or without considering geospatial metrics; and
- With or without considering metrics based on direct measures of water presence.

Thus, we developed 24 models to explore eight approaches (1 full vs. 5 subregional models, with or without geospatial metrics, with or without metrics based on direct measures of water presence). Analyses were conducted on data from the initial reach visits alone. For each of the 24 models, data were split into 80% calibration and 20% validation datasets, stratified by the 5 subregions and 3 streamflow duration classes.

#### 2.5.4. Model Calibration and Performance Evaluation

Models were fit for each of the 24 options identified in the previous step. We explored two types of models: random forest models, and models based on single classification trees (following the method of Nadeau and others [25] for the PNW). A method based on classification trees are easier for inexperienced practitioners to interpret and use, but are more prone to overfitting data than random forest models, which are based on large numbers of classification trees.

Random forest models were fit using the randomForest function in the *randomForest* package in R [48] using default parameters, except that the number of trees was set to 1500 instead of the default 500. Classification trees were fit using the rpart function in the *rpart* package in R [50]. Only the initial visit for reaches in the calibration dataset were used for model fitting.

Model performance evaluation focused on two aspects: accuracy and repeatability. Accuracy was assessed by calculating the same comparisons used to evaluate metric responsiveness during the metric screening phase (e.g., ephemeral versus at least intermittent reaches and perennial versus wet intermittent reaches; Table 2). Accuracy was measured using the initial reach-visit in both the calibration and validation datasets independently. We compared validation and calibration measures to see if models validated poorly, suggesting that they may be overfit.

Repeatability was assessed using data from the 12 reaches that were revisited and was calculated as the percent of reaches where classifications from both visits were the same (regardless of whether the classification is correct). Due to the limited amount of data, repeatability was only assessed on a region-wide basis, and not within each subregion.

#### 2.5.5. Selection of a Final Model

A final model was selected based on its performance, as well as the advice we received from the RSC. We presented performance measures (i.e., accuracy and repeatability) and other characteristics of final models to the RSC to provide feedback and advice on selecting a final model. In particular, we asked them:

- Is a subregionally stratified approach warranted?
- Should we include geospatial metrics in the model?
- Should we include direct measures of water presence in the model?
- Should we use a single decision tree or a random forest model?

##### Refinement and Creation of a Final Beta Method

After selecting a final model, we made several revisions to facilitate its use and acceptance by the management community, based on feedback and interactions with the RSC. As explained below, the RSC ultimately recommended a random forest model over a single decision tree, and many of these refinements were conducted with these types of models in mind. Performance of the refined model was re-evaluated following these modifications.

##### Refinement of Indicators

The metric selection process described above identified an optimal set of metrics to use in the SDAM, but it did so without considering difficulties in measuring each metric or effort required to measure all of the metrics. For example, rfe may have selected a metric based on the total number of aquatic invertebrates, even if there was little new information provided once 20 were observed. That is, field crews might be able to cease counting aquatic invertebrates once they found 20 individuals. Thus, we explored ways to simplify metrics in order to reduce the burden on field crews and facilitate use of the method (e.g., avoid reliance on access to statistical software). We also identified metrics that could be eliminated because they were closely related to another metric in the final method. Metrics that were more complicated to measure were rejected if a simpler to measure alternative was available, and continuous metrics were converted to binary or ordinal variables based on visual interpretation of random forest partial dependence curves. Accuracy and repeatability measures were re-evaluated to ensure that overall model performance was not substantially affected by the modifications.

##### Increased Confidence Required for Classifications

Random forest models, when used in classification mode, traditionally make assignments based on the class that receives the highest number of votes by each “tree” in the forest. Thus, in a 3-way decision, the “winning” class could receive much less than a majority of votes—as low as 34%. The RSC believed such low-confidence classifications may be insufficient for certain uses of the SDAM, and instead recommended exploring approaches to distinguish between high- and low-confidence classifications.

Based on this input from the RSC, we explored increasing the minimum number of votes required to make a confident classification from 50% to 100% by increments of 2.5%. When the final model was applied to a novel test reach and a single class received a sufficient percent of votes, then the reach was classified accordingly. If none met the minimum, but the combined percent of votes for intermittent and perennial classes exceeded the minimum, then the reach was classified as *At least intermittent*. In all other cases, the reach was classified as *Need more information*. This decision framework reflects the opinion of the RSC that distinguishing between ephemeral and at least intermittent reaches was a high priority use of the SDAM, more so than distinguishing between perennial and nonperennial reaches. We calculated the percent of reaches under each of the five possible classifications with increasing minimum vote agreement thresholds, and presented the results to the RSC to select a minimum threshold.

##### Addition of Single Indicators

Single indicators can supersede model classifications of *Ephemeral* or *Need more information* to *At least intermittent*. Single indicators provide technical benefits (i.e., improved accuracy), as well as non-technical benefits (such as greater acceptance of the SDAM, given public understanding of the role of streamflow duration in supporting wildlife), which is why they are used in most other SDAMs (e.g., [27,34,51,52]). We evaluated the following potential single indicators, based on recommendations from the RSC:

- Presence of live fish,
- Presence of live amphibians,
- Presence of any living aquatic vertebrate (fish, amphibians, or reptiles), and
- Live or dead (desiccated) algal cover on the streambed ≥10%.

We evaluated the number of instances where the change would correct a misclassification (i.e., the reach was truly intermittent or perennial), and the number of times it would introduce a misclassification (i.e., the reach was truly ephemeral).

#### 2.5.6. Evaluation of the Final Beta SDAM and Comparison with Other SDAMs Used in Portions of the AW

We applied the final beta SDAM model to the dataset and calculated the same accuracy and repeatability measures described above. We investigated reaches where the beta SDAM classifications did not correspond to known streamflow duration class for that reach or resulted in classifications of *Need more information*.

Classifications and performance measures from the beta SDAM for the AW were compared to the PNW method [27] and the NM method [34]. Our data collection only allowed classification following the first phase of the NM method, which may result in “gray zone” classifications of tentatively intermittent and tentatively perennial. For our assessments, these results were treated as intermittent and perennial, respectively.

### 2.6. Application to Two Focus-Area Studies

Each reach assessed as part of the focus-area studies was classified according to the final Beta SDAM AW. These classifications and underlying indicator values were presented to the practitioners. We then asked them whether the classifications agreed with their understanding of the study area, what elements of the protocol worked well, and which presented challenges. Finally, we asked for their thoughts on whether the final method would be suitable for their programs and monitoring needs.

## 3. Results

### 3.1. Identification of Candidate Indicators

We identified eight flow duration methods for temperate regions, two of which cover portions of the AW: the SDAM for the Pacific Northwest (the PNW method [27]), and the New Mexico method (the NM method [34]). Other methods that were identified covered arid regions in other parts of the world (e.g., Mediterranean Europe [53,54]), or non-arid portions of the United States (e.g., North Carolina [52], Ohio [55], Kentucky [56], Oregon [51], and temperate portions of the United States [57]; the Oregon method was a predecessor to the PNW method). From these methods, as well as the scientific literature, 12 geomorphological, 14 hydrological, and 15 biological candidate indicators were identified. Based on the initial screening, as well as precedented use in the PNW or NM methods, a subset of 6 geomorphic, 7 hydrologic, and 13 biological indicators were selected for further evaluation, as described by McCune and Mazor [35]. In addition, we identified five classes of geospatial indicators to explore in addition to these field-measured indicators (Table 1).

### 3.2. Identification of Candidate Study Reaches

Our efforts yielded 725 candidate reaches, of which 13% were “preferred” (meaning that multiple sources of hydrologic data were available). Across the region, 48% of these preferred reaches were perennial, 36% were intermittent, and 16% were ephemeral. Data from USGS stream gages were available for 87% of the preferred perennial reaches, and 67% of intermittent reaches, but only 42% of ephemeral reaches. The remaining reaches were designated as “acceptable”.

### 3.3. Data Collection

From the list of 725 candidate reaches, 100 were targeted for a sampling campaign that spanned 2018 and 2019 (Figure 2). We initially targeted preferred reaches and reaches with USGS gages, but we included acceptable reaches in order to achieve the desired number of ephemeral and intermittent reaches in each subregion, and to allow field crews to visit multiple reaches in a single day. Overall, 36% of ephemeral reaches had “acceptable” status, in contrast with 12% of perennial reaches and 23% of intermittent reaches.

Reach-visits with incomplete data, or where crews did not sample the intended location (e.g., tributaries close to the target coordinates, but where the true streamflow duration class could not be determined) were excluded from further analysis, yielding a final dataset of 89 reaches, 12 of which were visited on two occasions (Table 3).

### 3.4. Data Analysis

#### 3.4.1. Metric Screening

**Figure 3.**
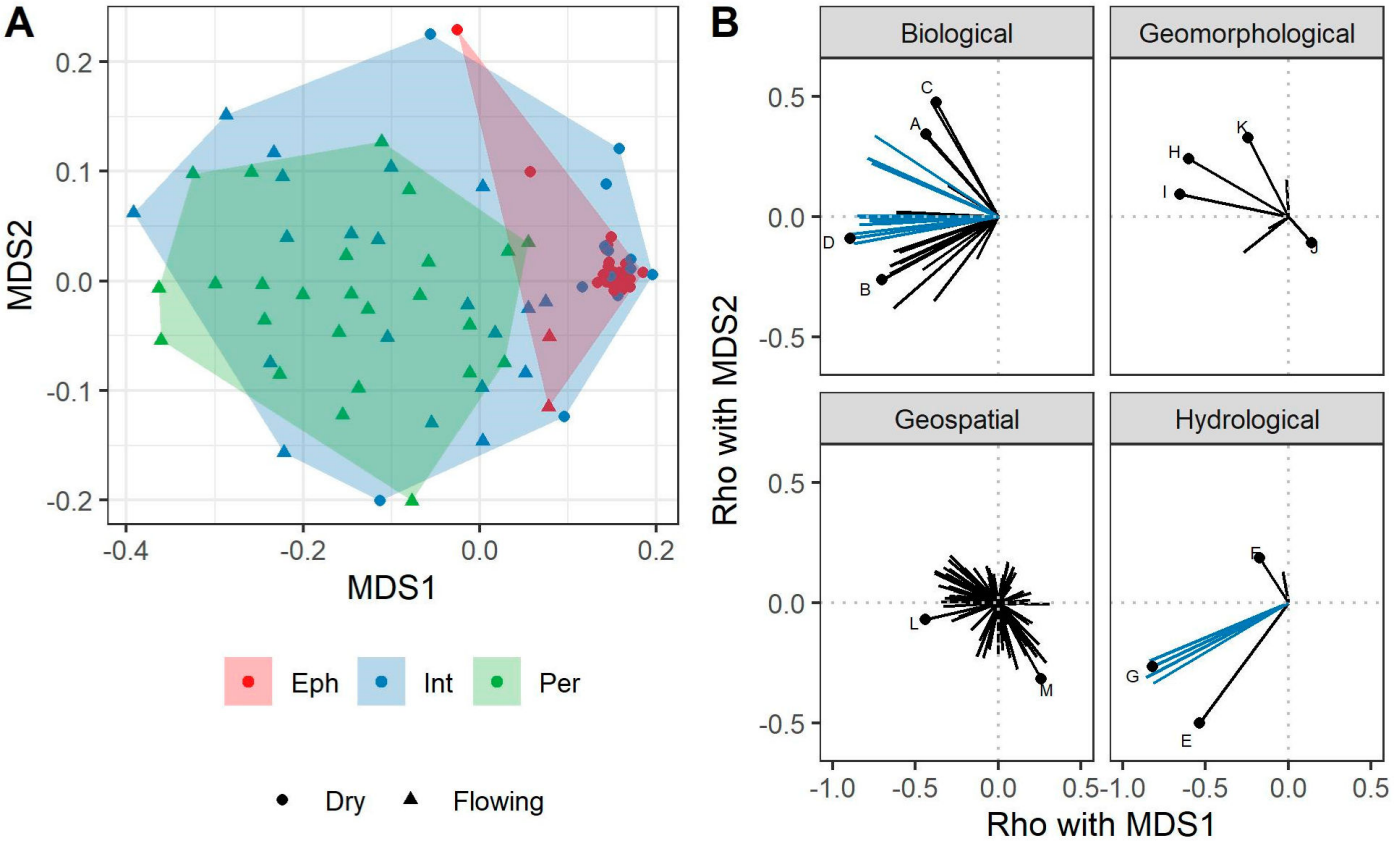
A two-axis nonmetric multidimensional scaling of metrics based on biological, geomorphic, and hydrologic indicators. (**A**) shows individual reaches. MDS: Multidimensional scaling axis 1 or 2. Eph: Ephemeral reaches. Int: Intermittent reaches. Per: Perennial reaches. (**B**) shows correlations (Spearman’s rho) between selected metrics and ordination axis scores; metrics with rho^2^ > 0.5 are highlighted in blue (no geomorphological or geospatial metrics had rho^2^ > 0.5, nor did any metric have rho^2^ > 0.5 with the second axis). Selected metrics are labeled: Biological metrics: A. Number of vertebrate types observed. B. Number of hydrophytic plant species observed. C. Algal cover on the streambed. D. Total abundance of aquatic invertebrates. Hydrological metrics: E. Presence of hydric soils. F. Presence of springs. G. Percent of reach with flowing surface water. Geomorphological metrics: H. Riffle-pool sequence score. I. Substrate sorting score. J. Mean bankfull width. K. Valley slope. Geospatial metrics: L. Mean annual precipitation in the watershed from 1981 to 2010. M. Watershed topographic wetness index. Correlations for all metrics are provided in Supplementary File S3.

Ordination of all 155 biological, hydrological, and geomorphic metrics using data from the first visit of all 89 reaches showed although ephemeral and perennial reaches were distinct from each other, intermittent stream reaches were highly variable and overlapped with the other classes (Figure 3). Ephemeral reaches were tightly clustered, indicating that they are relatively homogenous with respect to flow duration indicator metrics compared to perennial and intermittent stream reaches. Several biological and hydrological metrics were strongly correlated (i.e., rho^2^ > 0.5) with an ordination axis, but no geomorphological or geospatial metrics did. All of these strongly correlated metrics had negative relationships with the first ordination axis (i.e., the axis that separated perennial from ephemeral reaches), indicating that higher values of these metrics were largely indicators of longer flow durations, and no metrics had higher values at reaches with shorter flow durations. Metrics related to vertebrates were positively correlated with axis 2, but none with a rho^2^ > 0.5 (correlation coefficients are provided in Supplementary File S3). That is, there were no metrics for which high values were associated with ephemeral reaches.

The within-metric metric distribution and responsiveness screens shown in Table 2 applied to data from the first visit of all 89 reaches reduced the total number of candidate metrics from 155 to 38 biological metrics, 4 geomorphological metrics, and 5 hydrologic metrics, in addition to 53 geospatial metrics. Most metrics (95%) passed the % dominance criterion, in contrast to the more restrictive responsiveness criteria. Slightly more than half the metrics were able to discriminate among three streamflow duration classes (F > 2), and a similar number could distinguish between ephemeral and at least intermittent reaches (t > 2). However, only 23% could discriminate between ephemeral and dry intermittent reaches, and just 12% (mostly invertebrate metrics, and metrics related to Ephemeroptera, Plecoptera, and Trichoptera [EPT] taxa in particular) could discriminate between perennial and flowing intermittent reaches. Screening criteria for biological, hydrological, and geomorphological metrics that passed screens are shown in Table 4, and the full list of metrics are presented in Supplementary File S3.

#### 3.4.2. Metric Selection

**Figure 4.**
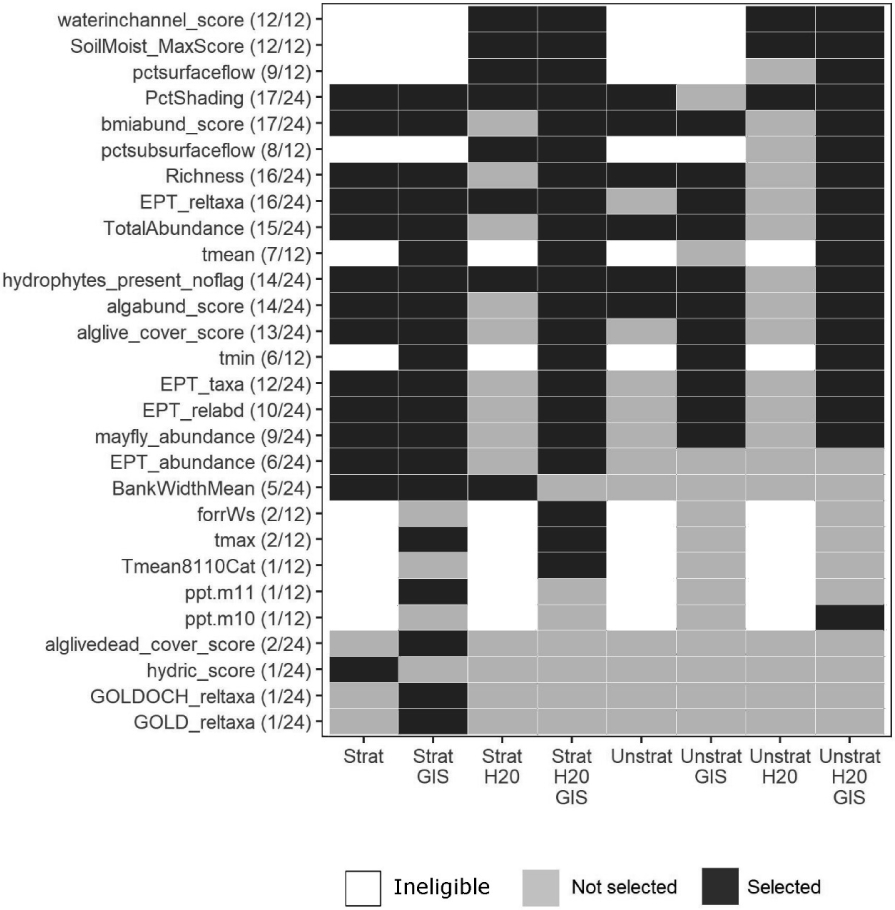
Summary of metric selection. Metrics are described in Table 4 and Supplementary File S3. Numbers in parentheses indicate the number of times the metric was selected, and the number of times the metric was eligible for selection. Strat: Stratified approaches. Unstrat: Regional (unstratified) approaches. GIS: Approaches that considered geospatial metrics. H2O: Approaches that considered direct measures of water presence. Each cell indicates if a metric was selected by recursive feature elimination applied to fitting random forest models. Black cells indicate that the metric was selected (for at least one subregional model in stratified approaches). Gray cells indicate that the metric was not selected. White cells indicate that the metric was ineligible for selection (either geospatial metrics or direct measures of water presence). Only metrics that were selected at least once are shown.

A total of 28 metrics were selected by recursive feature elimination for at least one of the 24 model iterations described in the Metric Selection section of the Methods above. Fifteen biological metrics, seven geospatial metrics, five hydrological metrics, and one geomorphological metric were selected at least one time (Figure 4). Some of the most frequently selected metrics include those related to invertebrate abundance, hydrophytic vegetation, relative abundance of EPT taxa, and algal abundance. Soil moisture (*SoilMoist_MaxScore*) and water in channel (*waterinchannel_score*) were selected every time they were eligible (i.e., in models that allowed direct measures of water presence).

#### 3.4.3. Model Calibration and Performance Evaluation

**Figure 5.**
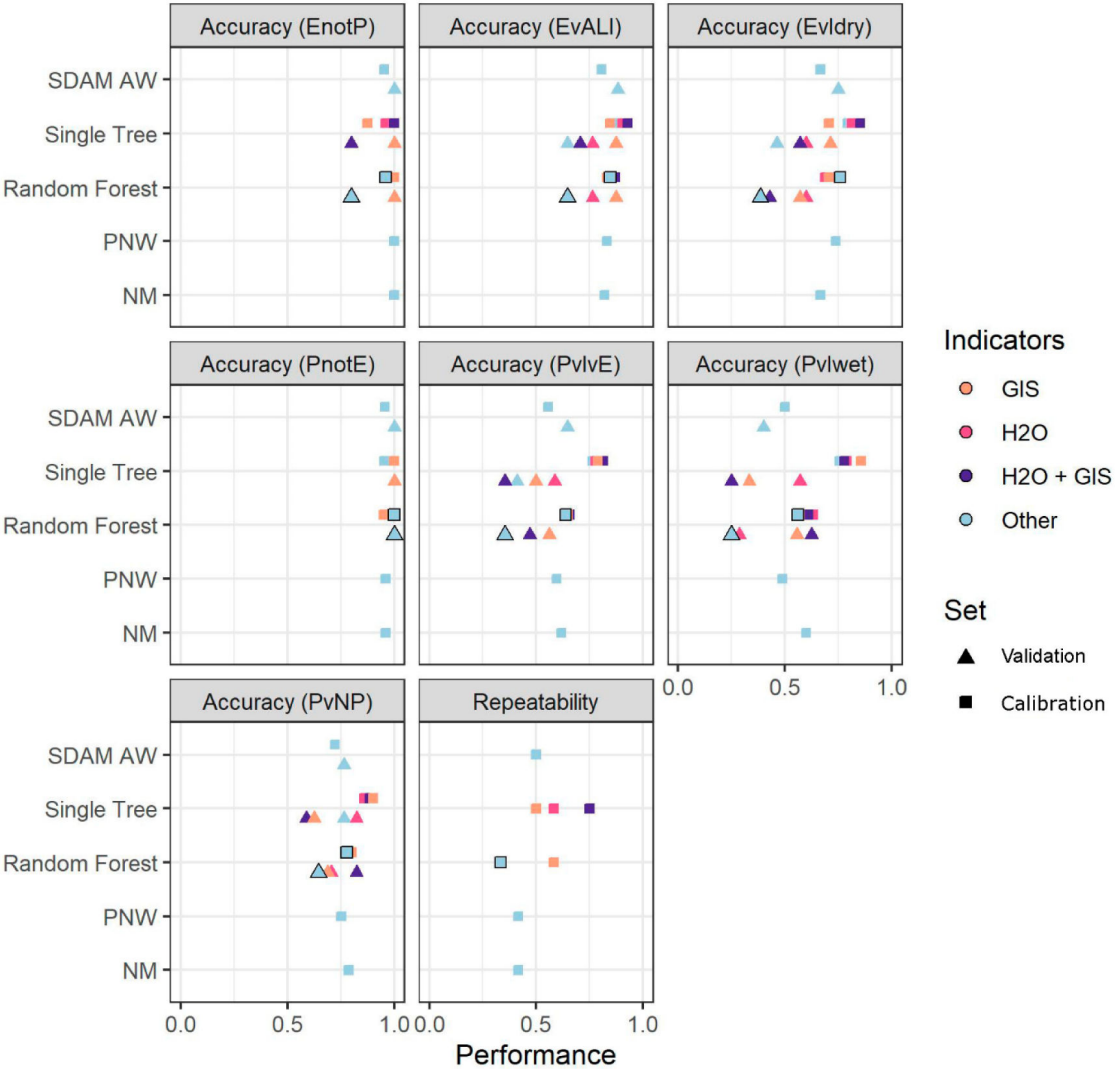
Performance evaluation measures. Accuracy measures are proportion of correct classifications. EnotP: Proportion of ephemeral reaches correctly not classified as perennial. EvALI: Proportion of reaches correctly classified as ephemeral or at least intermittent reaches. EvIdry: Proportion of dry reaches correctly classified as ephemeral or intermittent. PnotE: Proportion of perennial reaches correctly not classified as ephemeral. PvIvE: Proportion of reaches correctly classified as perennial, intermittent, or ephemeral. PvIwet: Proportion of flowing reaches correctly classified as perennial or intermittent. PvNP: Proportion of reaches correctly classified as perennial or non-perennial. Repeatability: Proportion of revisited reaches with the same classification for each visit. The outlined symbols represent final selected model, and SDAM AW represents the final, simplified version that includes single indicators. GIS: Models that include geospatial data. H2O: Models that include direct measures of water presence. Other: Models that either exclude geospatial metrics and direct measures of water presence, or results from the PNW and NM SDAMs. Results for stratified models are not shown, but are included in Supplementary File S4.

Performance measures for most models were similar, regardless of whether stratification was applied, or if geospatial metrics or direct measures of water presence were included. Accuracy in discriminating among the three flow duration classes ranged from 0.52 to 0.79 proportion of reaches correctly classified, but 0.77 to 0.88 when discriminating ephemeral from at least intermittent reaches. Repeatability ranged from a low of 0.33 (i.e., only a third of reaches had the same classification on two visits) to 0.78 (Table 5). In general, the single decision tree models showed a marked decline in some performance measures when independent validation data were evaluated (Table 5, Figure 5). There was little evidence of benefit from stratified approaches implemented at subregional scales, whether evaluated across the region (Table 5) or within individual subregions.

#### 3.4.4. Selection of a Final Model

The RSC was presented with results for a range of options for several factors in model construction: regional stratification, the inclusion of geospatial metrics, the inclusion of metrics that directly measure the presence of water, and the choice of a model based on single decision trees versus random forest. The RSC initially expressed a preference for whatever options provided the best performance. However, in the absence of clear differences, they made recommendations based on several non-technical concerns. For example, they advised against a sub-regionally stratified approach. The primary disadvantage of stratified approaches is that they introduced needless complexity. Several RSC members expressed skepticism that subregional stratification would be successful given the amount of available data.

The RSC expressed concerns about using geospatial metrics as indicators in the SDAM because indicators derived from NHD flowlines (e.g., StreamCat data) may not represent unmapped headwaters or other reaches where the SDAM is likely to be used [42], and because there was little clear benefit to including geospatial metrics, in terms of improved accuracy. Additionally, the RSC advised against including hydrologic metrics based on direct measures of water presence. Although such metrics might provide valuable supporting information in an assessment, including it in the SDAM could introduce circularity (i.e., using hydrologic data to classify actual flow duration in method development and to classify flow duration in applying the method), and reduce acceptance of the tool by certain communities, as previously described [1].

The RSC initially preferred single tree models over random forest models because the former are relatively transparent and easy to use in the field. However, they ultimately recommended a random forest model. The individual trees created in our calibration steps were relatively simple, and while this made them easy to use, they led to a number of outcomes the RSC felt were indefensible and would reduce acceptance of the method. For example, one of the trees we produced identified ephemeral streams based on the absence of aquatic invertebrates even though no signs of aquatic invertebrates were observed at 45% of dry intermittent streams. Such outcomes were likely the result of our relatively small dataset, which could only support the calibration of relatively simplistic trees. Random forest models avoid this scenario by incorporating a large number of trees, each with their own unique subset of “in-bag” calibration reaches, and reduce over-fitting. Thus, the RSC advised us to select a random forest model (contingent on ensuring such a complex method would remain accessible to practitioners), and to reconsider a single-tree approach after additional data collection.

**Figure 6.**
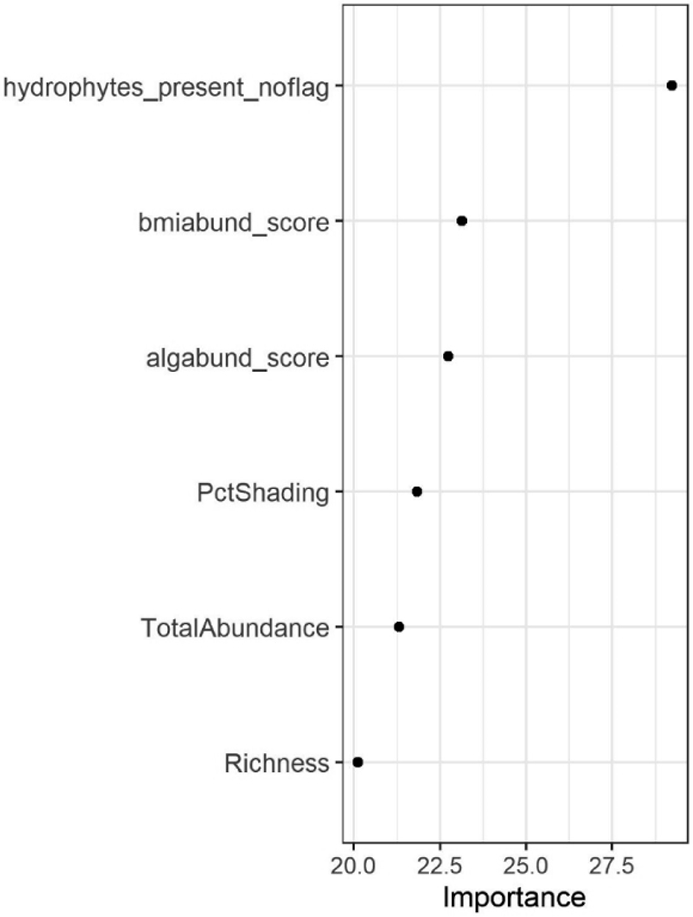
Variable importance (calculated as the % mean decrease in accuracy when that variable is removed) of the selected model—an unstratified random forest model based on six biological metrics. Metric descriptions are provided in Table 4.

Based on this feedback, we selected the random forest model that did not include geospatial metrics or direct measures of water presence. This model contained six metrics, all of which were biological (Figure 6). Two of the metrics were based on riparian vegetation (i.e., the number of hydrophytic plant species, *hydrophytes_present_noflag* in Figure 6, and stream shading cast by riparian vegetation (i.e., *PctShading*). Three metrics were related to aquatic invertebrates. Two of these measures were related to abundance (i.e., *TotalAbundance*, which is derived from the tally of individuals collected during sampling, and *bmiabund_score*, a qualitative assessment of abundance derived from the NM method), and one to taxonomic composition (i.e., *Richness*, family-level richness). The final metric was a qualitative measure of algal abundance following the NM method.

#### 3.4.5. Refinement and Creation of a Final Beta Method

Subsequent to the model refinements described below, the RSC recommended adoption of the method, henceforth called the streamflow duration assessment method for the AW (SDAM AW), as a “beta” method to be evaluated in an interim period during which additional data collection could occur, and feedback from end-users in the region could be obtained.

The accuracy and repeatability of the refined method versus the unrefined model based on metrics selected by recursive feature elimination can be evaluated by comparing the highlighted rows in Table 5.

##### Refinement of Indicators

We modified the selected metrics as described below. Our intent was to reduce the time and expertise required to measure indicators without sacrificing performance of the final method. In addition, we wanted to replace continuous metrics with ordinal categories that would be easier to measure and interpret. These two simplifications (i.e., reducing the overall number of metrics, and converting continuous metrics to ordinal metrics with few categories) would enable us to create an easy-to-use table that could provide the same function as a complex statistical model, but without the need for statistical software. Following these refinements, the performance of the final method was re-evaluated.

##### Riparian Vegetation

**Figure 7.**
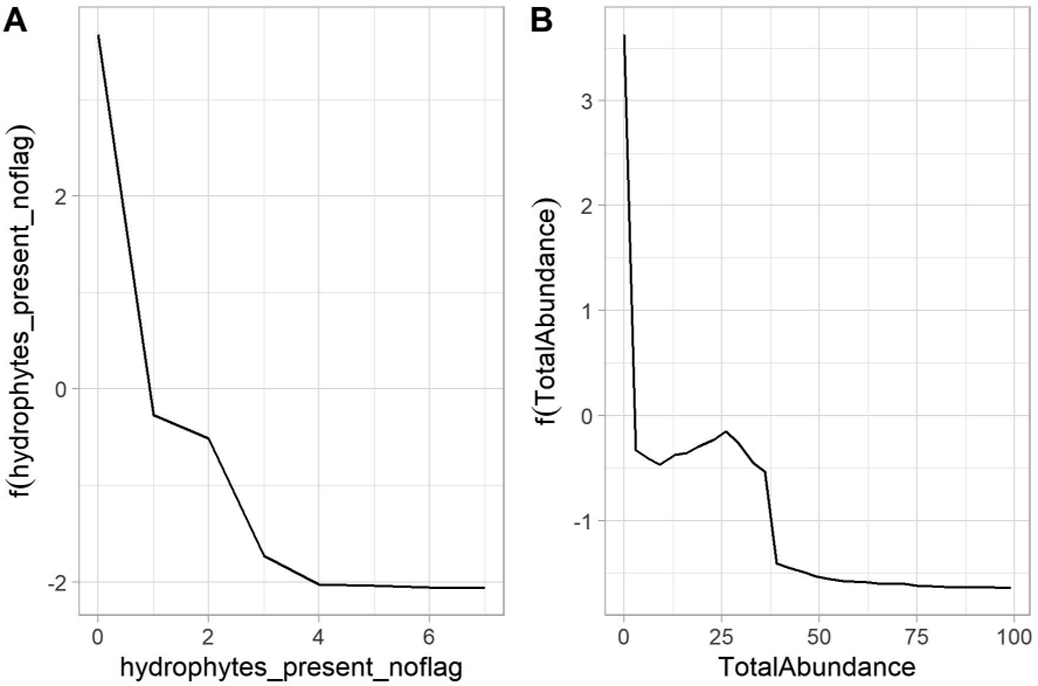
Partial dependence plots for two metrics included in the final model: (**A**). Number of hydrophytic plant species, and (**B**). Total abundance of aquatic invertebrates. Plots were generated using the pdp package in R [58].

Two metrics related to riparian vegetation were included in the final model: the number of hydrophytic plant species reported in the reach, and streambed shading. We selected only the former because it was a more direct measure of the plant community, whereas measures of shading may capture sources unrelated to streamflow duration, such as canyon walls or nearby structures. Furthermore, the use of hydrophytic plants in the PNW method as well as in jurisdictional wetland delineation methods meant that its inclusion would likely have a greater degree of acceptance among end-users. We converted the continuous measurement to three categories (no hydrophytic species observed, one to two species observed, or three or more species observed) based on inspection of partial dependence plots (Figure 7). Partial dependence plots illustrate how variation in a single predictor can affect the outcome of a model when other predictor values are held constant, and they can be used to identify important change-points in the relationship between an indicator and streamflow duration outcomes.

##### Aquatic Invertebrate Abundance

Similarly, two metrics related to aquatic invertebrate abundance were included in the final model: a qualitative assessment based on the effort required to observe aquatic invertebrates throughout the assessment reach, and a quantitative assessment based on a tally of collected, sorted, and identified organisms. We selected the latter because the subjective assessments were difficult to standardize and could be prone to differences in expertise among practitioners. We converted the continuous measurement to three categories (no aquatic invertebrates observed, one to nineteen individuals observed, or twenty or more individuals observed) based on visual inspection of partial dependence plots (Figure 7).

##### Aquatic Invertebrate Composition

One metric in the final model was related to aquatic invertebrate composition: taxonomic richness at the family level. Although family-level identifications are used in other SDAMs (e.g., [27]), many likely practitioners lack the training to generate these data. We therefore substituted this metric with a simpler to measure metric: the presence or absence of Ephemeroptera, Plecoptera, or Trichoptera (EPT) taxa. First, this metric only requires order-level identifications. In addition, EPT-related metrics were selected in most other models (Figure 4). Thus, this metric was a relatively simple way to retain information about the taxonomic composition of the aquatic invertebrate assemblage that was still relevant to streamflow duration assessment.

##### Algal Abundance

One metric of algal abundance was included in the final model: a qualitative assessment based on the effort required to observe algae throughout the assessment reach. As with aquatic invertebrates, this qualitative assessment is difficult to standardize (i.e., it is based on the level of effort required to observe algae, rather than a quantitative estimate of algal cover). Therefore, we substituted it with a more quantitative metric based on the total streambed cover of live or dead algal mats (excluding dead mats that were clearly deposited from upstream sources). The metric was originally measured in 5 categories (i.e., not detected, <2% cover, 2 to 10% cover, 10 to 40% cover, and ≥40% cover). We reduced this to a binary measure of presence/absence. As described below, algal cover ≥10% may be used as a single indicator, so practitioners may record this indicator in three categories (i.e., not detected, <10% cover, and ≥10% cover).

##### Increased Confidence Required for Classifications

**Figure 8.**
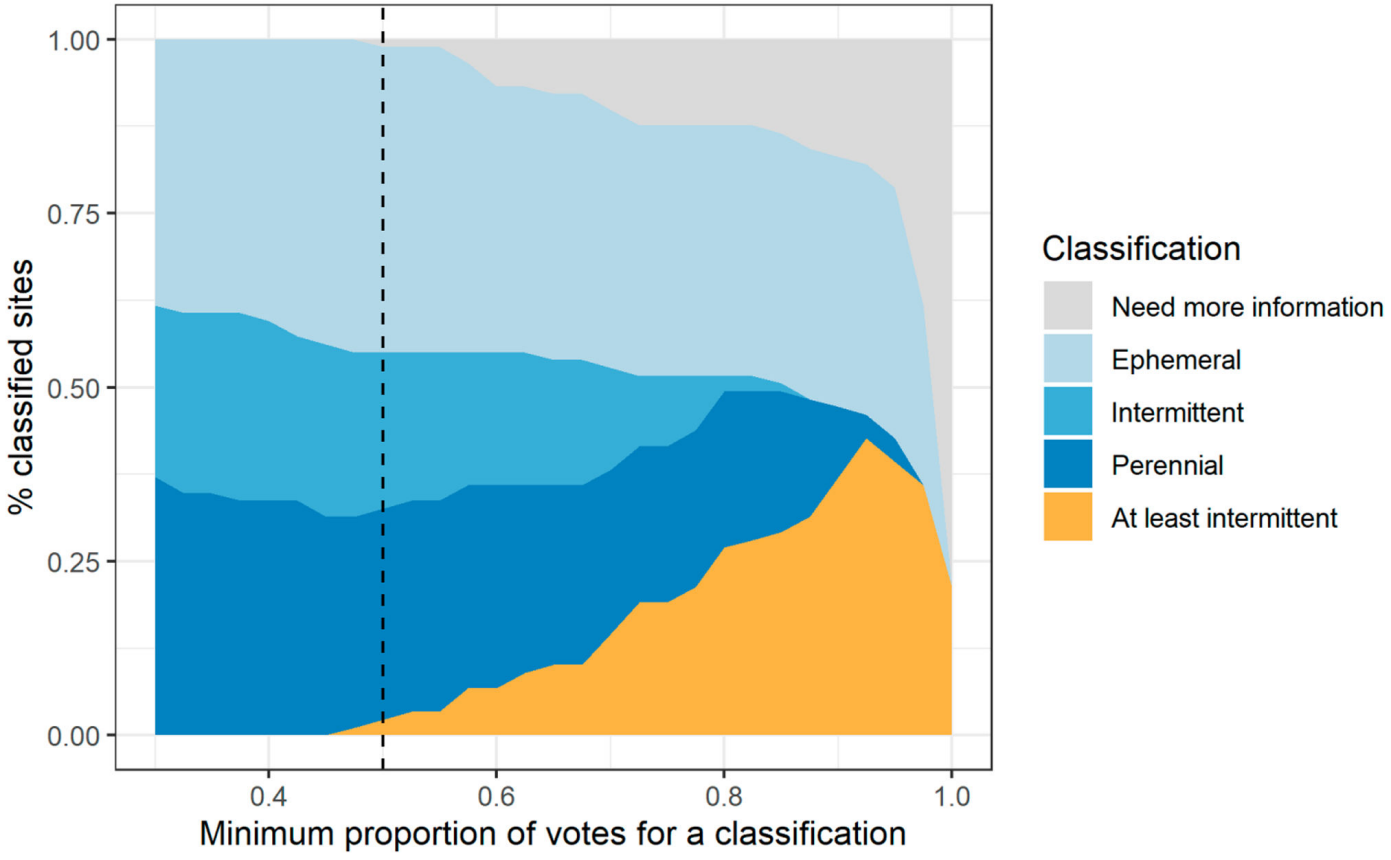
Percent of classified reaches versus the minimum proportion of votes from a random forest model required to make a classification. The dashed line represents the minimum proportion selected by the RSC (i.e., 0.5). This figure was created using data from both calibration and validation reaches.

Although increasing the minimum number of votes from a random forest model required to make a classification improved overall accuracy, it did so at the expense of being able to make precise classifications (Figure 8). For example, when 90% of the votes were required to make a classification, no reaches were classified as intermittent, whereas 28% were classified as *At least intermittent*. At the same time, the number of reaches where classifications could not be determined (i.e., *Need more information* classifications) increased to 17%. Based on these factors, the RSC recommended a minimum threshold of 50% of votes required to make a classification.

##### Addition of Single Indicators

Most of the single indicators that we considered had no impact on the accuracy of the method. For example, fish were only detected at reaches that were already classified as *Intermittent*, *Perennial*, or *At least intermittent*. Amphibians corrected a misclassification at one reach, but introduced a misclassification at another reach. The presence of ≥10% algal cover corrected three misclassifications without introducing additional misclassifications.

Based on these results, the RSC recommended inclusion of two single indicators: fish, because of their broad acceptance by the public as an important resource provided by perennial and intermittent streams, and algal cover ≥10% because it improved accuracy. Amphibians were not recommended because they decreased the method accuracy, although they endorsed the reporting of amphibians as supplemental information when conducting assessments.

#### 3.4.6. Evaluation of the Final Beta SDAM and Comparison with Other SDAMs Used in Portions of the AW

Because the method was based on a small number of indicators with few categories, it was possible to generate a table to crosswalk all possible combinations of indicator values to their outcomes in a simple table (Table 6). Thus, practitioners would be able to obtain a classification without the use of statistical software to run the underlying random forest model.

Among the 89 reaches in the development dataset, the Beta SDAM AW had better success at classifying ephemeral reaches (80% correct, and 81% if revisits are counted) and perennial reaches (84% and 76%) than intermittent reaches (50% and 54%; Table 7). Two ephemeral reaches sampled under flowing conditions were both classified as *Need more information*, as were three of the ephemeral reaches sampled under dry conditions. Surprisingly, two dry reaches were classified as perennial. One reach was thought to be truly intermittent based on gage data (Sabino Canyon, USGS gage 0948400), but it had large permanent pools that likely contributed to the high levels of indicators observed. The other reach (a reach on Cottonwood Creek in Arizona) was thought to be truly ephemeral, and although it lacked water at the time of sampling, several hydrophytes and caddis cases were observed, as well as algal mats, damp soil, and adult amphibians. The original determination was based on a single year of data from a wildlife camera, and thus may have underestimated the extent of flows at this reach. Intermittent reaches were more likely to be correctly classified when they were flowing (64%) than when they were dry (38%). Among the 17 reaches classified as *At least intermittent*, about half were perennial and half were intermittent. No perennial reaches received a classification of *Need more information*. There was no apparent relationship between error rates and whether the original classification of a reach was “acceptable” or “preferred”.

The SDAM AW’s performance was comparable to those of the PNW and NM methods (Table 5, Figure 5, Supplementary File S4), and they agreed more than two-thirds of the time (Table 8). While the overall rates of agreement between the beta SDAM AW and the other methods were nearly identical, there were large differences in how intermittent or perennial reaches were classified. For example, for reaches classified by the SDAM AW as perennial, the NM method agreed 91% of the time, whereas the PNW agreed only 55%; in contrast, the NM agreed with the beta SDAM AW’s classifications of intermittent only 30% of the time, whereas the PNW method agreed 65%. For reaches classified by the beta SDAM AW as “need more information”, the other methods were both more likely to classify these reaches as intermittent. Based on this comparability, the RSC determined that programs that had been relying on the NM or PNW method could switch to the beta SDAM AW with little consequence for the majority of reaches they are likely to encounter.

### 3.5. Application to Two Focus-Area Studies

Results from the two focus-area studies are presented in Supplementary File S5.

## 4. Discussion

### 4.1. The Beta SDAM AW Can Support a Range of Management and Monitoring Needs

Following the process described in [1], we were able to develop an effective SDAM that may be used to classify reaches in the AW where streamflow duration information is needed. The accuracy with which the SDAM AW distinguishes ephemeral reaches from at least intermittent (81%) is lower than accuracy reported for other methods (e.g., 94% in PNW [25], 96% in NM [34]) but should still be sufficient for many applications. The finding that the beta SDAM had greater success classifying ephemeral and perennial reaches than intermittent reaches corroborates previous studies evaluating indicators and assessment methods of streamflow class [16,25,59]. The RSC wanted an unbiased method that did not consistently over- or under-estimate streamflow duration, but they prioritized the ability to discriminate between ephemeral and at least intermittent reaches over the ability to discriminate between perennial and non-perennial reaches. The performance of the beta SDAM AW indicates that it is sufficient for use in research and management applications where streamflow duration information is needed during the beta testing period.

Although methods were already available for portions of the AW [27,34], the development of this method on a large geographic scale greatly reduces uncertainty about assessing streamflow duration, particularly in areas outside the intended scope of existing methods. Nonetheless, the relatively small dataset (e.g., 89 reaches vs. 264 reaches in the PNW method), combined with the lower than desired accuracy in distinguishing perennial from intermittent streams suggests that improvements may be possible with additional data collection. Thus, the beta method we present here will be used during an interim period while data collection continues. In addition, this testing period creates an opportunity to solicit additional feedback from users, which may further improve the performance, ease of use, and acceptance of a final method.

The SDAM AW has already been adopted into a few management and monitoring programs. For example, the Arizona Department of Environmental Quality has begun collecting data with the full development protocol in order to determine where to apply ephemeral, intermittent, and perennial aquatic life uses in the Hassayampa River (see focus-area studies in Supplementary File S5). Outside of regulatory contexts, the Stormwater Monitoring Coalition of Southern California is using the method to map streams and identify reaches where future bioassessment may be warranted [60].

### 4.2. Strengths and Limitations of the Beta SDAM AW

The beta SDAM AW combines the strengths of both the PNW and NM methods, while minimizing their weaknesses. Like the PNW method, the SDAM AW relies on a small set of objectively measured indicators, each linked with streamflow duration. We were able to avoid including the subjectively assessed indicators of the NM method, and it did not include any geomorphological indicators that reflect aspects of the hydrologic regime unrelated to duration (such as magnitude or stream power). However, like the NM method, the SDAM AW can withstand a degree of error and sampling variability without greatly affecting the likelihood of obtaining a correct classification. This robustness is due to the multiple pathways through which one can arrive at the same classification, despite the low number of required indicators (Table 6). In contrast, the PNW’s decision tree has relatively few pathways to any one classification, and it is highly vulnerable to errors where indicators are missed or misidentified.

The beta SDAM AW method has room for improvement. The relatively poor ability to distinguish perennial from intermittent streams may be due to the possibility that the indicators themselves are not strongly different in the two classes of streams, at least at the level of effort we used to measure them. For example, many studies show that aquatic invertebrate communities have significantly different composition in intermittent and perennial streams (e.g., [19,61-63]), these differences are less apparent when assessed at a more coarse (e.g., family-level) taxonomic resolution (e.g., [64,65]). It is likely that many reaches in the AW experience drying with some frequency, even if they are apparently perennial based on available periods of record from stream gages [3]; thus, the biota of both perennial and intermittent streams may be relatively similar, as both stream types would demand life histories that are adapted to dry conditions. Although additional data collection may improve the ability to distinguish between these stream types, it is possible that, at least in the AW, the similarity of these streams with respect to ecological measures and other indicators may limit the potential for improvement.

Although the beta SDAM appears to work in the majority of settings we evaluated, a few may present challenges. For example, streams managed as flood control channels may undergo frequent maintenance to remove some or all vegetation in the assessment area. Although some biological indicators recover quickly from these disturbances, the results from assessments conducted shortly after such disturbances may be misleading. Poor water quality in streams may also affect biological indicators—notably, the presence of EPT taxa. Indeed, several studies have documented the absence of these sensitive taxa in effluent-dominated rivers in the Southwest (e.g., [66-68]). However, upgrades to water treatment plants can lead to a recovery of mayfly taxa [69]. Consequently, the SDAM AW may fail to identify perennial systems as *Perennial* in situations where water quality has been severely degraded by wastewater or other types of stress such that EPT taxa are eliminated. The SDAM AW includes other biological indicators that are less affected by poor water quality, and therefore it will typically classify such streams as *At least intermittent*.

We need to provide better guidance on resolving ambiguous *Need more information* classifications. Although this classification was uncommon in our development data, it may be more common in real-world applications, which are likely to focus on borderline ephemeral/intermittent reaches, where ambiguous outcomes could result in contentious management decisions. The focus-area study in Arizona confirmed that *Need more information* was more common in real-world applications than expected, based on results from our development data (Supplementary File S5). To support managers needing to resolve these ambiguous classifications, we include a number of resources and supplemental information in the user manual to help them make a classification [70], such as evaluating additional information gathered during the assessment (such as the presence of aquatic vertebrates or aquatic invertebrate families that prefer long-duration flows), or gathered through desktop analysis (such as reviewing databases of historic aerial imagery, or the USACE’s Antecedent Precipitation Tool [71]). However, more structured guidance would be helpful.

### 4.3. Indicators Used in the Beta SDAM AW Have a Strong Conceptual Link to Streamflow Duration

Biological indicators appear to be particularly well suited for streamflow duration assessment. The SDAM AW consists exclusively of biological indicators, and no other indicator types were selected in our data-driven metric selection process. Biological indicators are widely used in ecological assessments because of their ability to integrate and reflect long-term variability in conditions due to their diverse life histories [72], and it is this same quality that makes them excellent tools to measure streamflow duration and other hydrological impacts [1].

Indicators can represent responses or controls of streamflow duration [1]. Although a number of geomorphological and geospatial “control” metrics did pass the distribution and responsiveness criteria (Table 2) and were identified as candidate indicators, only biological response indicators were selected by statistical analysis for inclusion in the beta AW method. In contrast, a number of stream classification models developed for parts of the AW [4] or include the AW [5,7,8,31] use geospatial predictors that characterize climatic, physiographic, geologic, and land cover controls at the scales larger than an assessment reach. Measures collected remotely and/or calculated from spatially and temporally coarse datasets may not be able to capture variation at smaller scales that can be captured by reach-scale field measurements [73]. However, it is likely that physical controls of streamflow duration within stream reaches are best represented by a combination of watershed and local reach-scale factors that describe the dynamic balance between flow sources and losses from infiltration and evapotranspiration [10].

Many of our study reaches did not coincide spatially with the pour point of subcatchments. Unlike previous studies [4,5,8] that used primarily reaches with stream gages that are often positioned near the base of subcatchment, our study design sought to have reaches evenly distributed across ephemeral, intermittent and perennial reaches. This design meant that many of our study reaches were distant from the pour-point of catchments recognized by geospatial datasets, like StreamCat [37], and the associated subcatchment data may not reflect the actual upstream characteristics. This may, in part, explain why relatively few geospatial metrics were identified candidate indicators in our study relative to previous studies. As the spatial and temporal resolution and continuity of geospatial datasets improve through improvements in remote sensing technology and user platforms, we expect that those datasets will become more frequently utilized in streamflow duration classifications.

Although hydrologic indicators were among the 155 candidate metrics in our study (Table 1), most of them were ultimately excluded from consideration because those data were used to confirm the direct flow duration classification. For example, if a stream thought to be ephemeral had flowing water during sampling, we conducted additional investigations (such as contacting local experts) to determine if a reclassification was warranted. Direct hydrologic measures or metrics have been identified as important predictors in other AW classification models [4,5,8]. The degree of circularity of using hydrologic metrics will depend upon whether or not and to what extent those measures were used in the original streamflow duration classification.

#### 4.3.1. Hydrophytic Plants

Our finding that hydrophytic plants are a good indicator of streamflow duration is well supported by several studies in the AW (e.g., [25,74-76]). Caskey and others showed a decrease in wetland plant occurrence after diversion of perennial flow along stream reaches in the Routt National Forest, CO [76]. Reynolds and Shafroth noted a number of plant species indicative of perennial versus intermittent flow regimes in high- and low-elevation streams in the Colorado Basin [77]. Although that study did not identify ephemeral streams, the authors report that the driest streams in their study were dominated by upland plants, such as sagebrush and juniper (Lindsay Reynolds, personal communication). Thus, the taxonomic composition of riparian and wetland plants appears to be a well-supported indicator of flow duration.

An advantage of riparian plants over other biological indicators of flow duration is that they are non-motile organisms, some of which have very long lifespans (i.e., decades). Therefore, they are well suited to reflect local, long-term conditions in a way that fish or invertebrates cannot. Another factor that sets them apart from other biological indicators is that they function both as responses to streamflow duration gradients, and, through their effect on evapotranspiration rates, a driver as well (e.g., [78,79]).

#### 4.3.2. Aquatic Invertebrate Abundance and Composition

Numerous studies have demonstrated strong relationships between flow duration and the composition of aquatic invertebrates (e.g., [15,19,22,64,65,80]), although only a few have reported effects on abundance [25,81]. Within the AW, Bogan and others report comparable levels of abundance in perennial and intermittent headwater reaches, both of which were considerably lower than downstream perennial river reaches [62]. Despite this relatively limited evidence about the efficacy of invertebrate abundance, it is a widely used indicator in other SDAMs [35], including both the PNW and NM methods.

In contrast to abundance differences, compositional differences in aquatic invertebrate assemblages of perennial and intermittent streams are well documented in the literature. Most studies report higher richness or diversity at reaches with longer duration, particularly among EPT taxa [82,83]. Several studies identify individual taxa as indicators of perennial flow (e.g., [82,84-86]), while a few identify taxa that prefer intermittent flow (e.g., [87,88]). In general, intermittent reaches within arid regions appear to support a subset of the taxa found at perennial reaches in a region, rather than a distinct set of taxa [81], although a few intermittent specialists are known (e.g., the stonefly *Mesocapnia arizonensis* [89], the fishfly *Neohermes filicornis* [90], and several genera of Chironomidae [91]). However, our reliance on family-level identifications likely limited the influence of these taxa on our method.

Studies on the effects of flow duration on aquatic invertebrates almost exclusively focus on intermittent and perennial reaches, presumably because of the difficulty of collecting aquatic taxa from ephemeral reaches. However, one study collected diverse macroinvertebrates from ephemeral streams sampled shortly after the onset of flow [92]. Clarke and others collected macroinvertebrates from debris jams in an ephemeral stream, which retained moisture throughout the dry season [81]. In our study, no dry streams were noted as having woody jams that harbored moisture or sustained aquatic invertebrates, suggesting that the microhabitat studied by Clarke and others ([81]) may not be prevalent in the AW. Thus, it appears that aquatic invertebrates are only rarely observed in ephemeral streams sampled under typical hydrologic conditions.

#### 4.3.3. Algal Indicators

Although algae may begin to grow immediately following inundation, extensive growth of mats and other forms that are easily visible typically requires multiple weeks of flow [93-96]. We did not observe evidence of algal growth in any ephemeral stream in our study, but the rapid growth rate of algae (particularly in unshaded reaches) may lead to overestimates of flow duration from this indicator if ephemeral reaches are sampled shortly after flow events. However, Robson and others note that recolonization is slowest in hydrologically isolated streams that lack perennial refugia, which likely limits the opportunity for growth of algal mats in most ephemeral streams [94].

The persistence of dead or desiccated algal mats contributed to the beta SDAM’s ability to discriminate between dry ephemeral and dry intermittent stream reaches. Although many algal species are resistant to desiccation, the breakdown of algal cells begins within a few hours of exposure to air [96-98]. The persistence of particulate organic matter in dry streams has been well documented (e.g., [99-101]), although most studies focus on leaf litter rather than algal mats. Robson noted that dry algal biofilms are often visible in rocky intermittent streams in Victoria, Australia, and it is likely they are conspicuous features of intermittent streams in most arid regions of the world [102]. Although the breakdown of dried algal mats may be accelerated by terrestrial scavengers (e.g., tetrigid grasshoppers [103]), our own observations suggest that in arid climates, visible evidence of algal growth often persists throughout the dry season.

### 4.4. Lessons Learned about SDAM Development

This study demonstrates that the steps outlined by Fritz and others [1] support the successful development of an SDAM. Our experience in developing a beta SDAM for the AW reinforces the importance of several steps highlighted by Fritz and co-authors and brings a few new ones to light.

#### 4.4.1. Engage End-Users throughout the Development Process

Engagement of representatives of diverse groups into the development of environmental assessment tools facilitates acceptance of final products, particularly if tools will be used for regulatory purposes [104], and is a key principle for open science for applied environmental research [105,106]. The RSC played a key role in guiding method development, reviewing interim products, and vetting major decisions. They provided technical insights, local knowledge, and connected us with valuable resources informing the conceptual framework of building an SDAM (indicators, study reaches, and hydrological data).

While the RSC was limited to staff from federal regulatory agencies, members provided the perspective of both end-users and implementers to the development of the beta method. An important driver behind release of a beta method prior to a final method in the process steps described by Fritz and others [1] (Figure 1) is that it provides a meaningful feedback opportunity for affected sectors and the wider end-user community. As end-users, the RSC helped assure that the beta method is relevant, rapid, user friendly, and likely to be accepted by the user community. As representatives of the implementing agencies, the RSC also is playing an important role, post-beta release, in engaging diverse groups and end-users through outreach and training during the beta period, assuring that a wide range of input is considered in creating the final method. The RSC is poised to play a similar role in the production and implementation of the final method and forms the core of a trained regional practitioner network.

The focus-area studies proffered an additional feedback opportunity through engagement with study partners: a state regulatory agency and a private consultant working on behalf of regional stormwater agencies. We received valuable insights regarding on-the-ground usability of method components (e.g., the user manual, field forms, and data management systems), level of effort for application, and consistency of results. In describing challenges and needs, these partners contributed to many improvements during the development of the beta method and helped us consider how to produce a tool that is useful for multiple management needs.

#### 4.4.2. Statistical Complexity Does Not Need to Create a Barrier for End-Users

We used random forest statistical models because of their ability to handle complex, non-linear relationships that are common to ecological and hydrological data [107,108]. These characteristics make random forest and related machine learning methods increasingly popular in environmental research, yet their complexity and dependence on specialized software creates challenges for communication, as well as for adoption into programs where the typical end-users have little familiarity with machine learning methods [109]. However, by converting the indicators into simple categorical metrics, we were able to create a table to crosswalk metric values to outcomes of the random forest model (Table 6). Thus, no expertise is required to make use of the complex statistics underpinning the beta SDAM.

#### 4.4.3. Poor Documentation of Ephemeral Streams Creates Major but Surmountable Challenges

Although ephemeral reaches are recognized to be widespread globally [31], very few are documented with sufficient rigor to support their use in SDAM calibration. Careful interpretation and verification are necessary when using the flow classifications reported in studies for SDAM development. Terminology and definitions used in reports or studies varies considerably [9], and a stream described as ephemeral in one study may be more appropriately described as intermittent using the definitions above. Studies may differ in how snowmelt affects classification, or how many flowing days distinguish ephemeral from intermittent streams. For example, Jaeger and Olden identified ephemeral reaches as those with <5% flowing days [40], while the threshold used by Hedman and Osterkamp was twice as high [110]. Some hydrology studies may identify reaches as ephemeral based on rigorously documented ephemeral flows (e.g., [111]), yet longer-duration flows that occur outside the study period may be undetected or unreported.

Due to the inconsistent terminology applied to non-perennial streams in the literature, evaluating hydrologic data may be the most reliable way to identify ephemeral reaches. When studies provide access to the underlying hydrologic data, these data may be re-examined and classified according to a potentially different but within-study consistent set of rules. However, long-term hydrologic data are rarely collected from ephemeral reaches, and the availability of reliable information may vary widely across the country based on state and local monitoring programs, regulatory treatment of ephemeral streams, and the density and perceived importance of ephemeral streams. In the absence of reliable data, SDAMs can use tools like baseline monitoring via loggers, as well as observations from local experts (see next section) to classify streams as ephemeral [1,9]. However, baseline monitoring may require long-term support in order to generate reliable data that can be used to identify ephemeral reaches with high confidence in light of year-to-year variability.

#### 4.4.4. Make the Best Use of Local Expertise

Local expertise allowed us to fill in crucial data gaps (e.g., finding ephemeral streams in regions where they were poorly documented). Local experts came from a wide range of backgrounds, such as hydrologists, engineers, monitoring specialists, professional and non-professional (citizen) scientists, park rangers, and university researchers. Without their assistance, it may have been impossible to identify enough ephemeral reaches for method calibration.

Given the importance of local expertise, we need better ways to interpret, standardize, and make use of this data source. Our quality assurance process characterized how well local experts knew a study reach based on years of experience and recency of visitation (Supplementary File S5), but this process may not have been sufficient to gauge how well expert definitions of streamflow duration classes matched our study requirements. Few experts reported more than 5 years’ experience with a reach, which may be insufficient to assess the frequency of flow events that might sustain hydrophytes in what might otherwise appear to be an ephemeral system. Given the long-term perspective required to understand streamflow duration, traditional ecological and hydrologic knowledge may be especially important in verifying streamflow duration classes (e.g., [112-116]).

#### 4.4.5. Recognize the True Complexity of Streamflow Duration Gradients

To calibrate the SDAM, study reaches were classified into one of three categories. Streams exist along a continuum of flow duration, and the length of flow events is just one dimension of this variability in addition to seasonality, timing, predictability, and frequency [3,9,13]. Thus, each class of stream reaches encompasses a great deal of hydrologic variability, which certainly impacted our efforts to develop an SDAM. In this study, we made no effort to focus on streams that characterize “extreme” or ideal representations of their class, nor did we try to exclude borderline cases (such as reaches that exhibit intermittent flow in only wet years). It remains an open question whether our strategy is more effective for SDAM development (because the calibration data represents the full range of hydrologic conditions found in the AW), or if we would have had greater success by focusing on non-borderline reaches. This question may be investigated with larger datasets generated in the future.

### 4.5. Future Research and Method Development Needs

#### 4.5.1. Investigate the Persistence of Indicator Expression at Reaches That Have Undergone Changes in Streamflow Duration

In general, reaches that have recently experienced a long-term change in flow duration or are in transition from one flow duration class to another should not be used for development of SDAMs. However, evaluation of such reaches could provide insight into timelines of how indicators respond to these changes. This research would allow managers and researchers to understand whether a streamflow duration assessment is providing information about present-day or historic conditions. Such questions may arise when making assessments on reaches that have undergone changes due to either natural or anthropogenic causes. Several studies have documented the slow decline of long-lived hydrophytic trees following diversions or groundwater extraction, whereas studies of other indicators (e.g., short-lived plants, invertebrates) tend to show a more rapid response (e.g., [76,117,118]). Therefore, it may be possible to identify indicators that can detect reaches that have transitioned from one flow duration class to another.

#### 4.5.2. Address Challenges Created by the Dependence on Taxonomic Expertise

Feedback from both the RSC and the practitioners of the watershed studies indicated that field-based family-level identification of aquatic invertebrates would be a challenge for widespread use of an SDAM. This was true even for practitioners that had extensive backgrounds in bioassessment and experience identifying aquatic invertebrates in lab settings. We addressed this challenge by requiring only identification of three insect orders (Ephemeroptera, Plecoptera, or Trichoptera), which all practitioners agreed could be reasonably achieved through brief trainings. However, order-level identifications may not have the same ability to discriminate between intermittent and perennial reaches as genus or species level identifications. It is likely that better taxonomic resolution is required to detect the influence of flow duration on aquatic invertebrate assemblages [65,85,88]. These challenges may be overcome either by requiring higher taxonomic resolution, perhaps in a tiered approach following the NM method [34], or by harnessing new technology for taxonomic data generation, such as DNA barcoding (e.g., [119,120]), or automated image recognition (e.g., [121,122]). Taxonomic expertise is also required for the identification of hydrophytes, although this barrier is less substantial than for aquatic invertebrates among practitioners typically involved in wetland delineation. Notwithstanding the considerable diversity of riparian plants in the AW [123-125], only a small number of species accounted for the majority of hydrophyte observations in this study. For example, at least one of four willow species (*Salix exigua*, *S. goodingi*, *S. laevigata*, and *S. lasiolepis*) were observed at 63% of intermittent or perennial reaches. Therefore, new practitioners need only develop expertise in identifying a handful of species to use the method.

#### 4.5.3. Get More and Better Hydrologic Data

Development of the beta SDAM AW highlighted the need for increased spatial and temporal resolution of hydrologic data collection in the AW at non-perennial stream reaches, as anticipated in Fritz et al. (2020). An analysis based on the National Hydrography Dataset at the 1:100,000 scale showed that non-perennial streams comprise approximately 59% of the total stream length in the U.S., excluding Alaska; however, intermittent and ephemeral streams are more highly concentrated in the western U.S. (e.g., 94% of Arizona’s stream length was found to be non-perennial [21]). Despite a high proportion of non-perennial streams in our study region, identifying documentation of non-perennial stream classifications was a primary challenge for developing the beta SDAM. We expect to experience the same challenge in developing SDAMs for other regions of the country. We anticipate that the rising interest in mapping and modeling non-perennial streams coupled with the availability of low-cost data loggers [41,126] may increase the availability of continuous hydrologic data for characterizing ephemeral streams to support SDAM development. The AW would also be a strong candidate region to explore and test remote sensing options (e.g., daily satellite imagery) to fill in hydrologic data gaps at certain reaches, such as at large rivers in the desert with low canopy cover, potentially in conjunction with hydrologic models [127]. As described by Fritz and others, future efforts should focus not just on increasing spatial coverage of flow records but also on increasing the temporal coverage of flow records for intermittent and ephemeral streams [1]. Long-term records are required to ensure that the flow classification is not influenced by a year or two of atypical data (e.g., due to droughts or floods) and to identify changes in hydrologic regime due to anthropogenic influences (e.g., water withdrawals) or climate change. Recent advances in timeseries statistical analyses have enhanced our ability to characterize streamflow duration, even when gage or logger records are interrupted by gaps created by instrument malfunction or ice-jams [128,129].

#### 4.5.4. Identify Positive Indicators of Ephemeral Streamflow Duration

All the indicators in the beta SDAM AW were positively correlated with streamflow duration. That is, they were present with higher frequency, abundance, or diversity at perennial or intermittent streams than at ephemeral streams. Thus, ephemeral status is inferred from lower values or the absence of these indicators. We recognize that indicators whose presence can be interpreted as positive evidence of ephemeral status could increase confidence in these classifications. However, our review of the literature and discussions with experts identified few such indicators that were practical to explore in SDAM development [35]. One potential indicator from the NM method (prevalence of upland plants in the streambed) was able to discriminate between perennial and non-perennial reaches, but not between ephemeral and intermittent reaches (Table 5), suggesting it may not be appropriate to interpret it as a positive indicator of ephemerality. It was not selected for inclusion in the model, presumably because other metrics provided greater discriminatory power. Positive indicators of ephemerality may lead to greater confidence in the acceptance of ephemeral classifications from an SDAM. Some studies suggest that terrestrial arthropods and non-hydrophytic plants may serve as useful indicators [77,130,131], but further exploration is needed to incorporate them into SDAMs.

## 5. Conclusions

This study illustrates the successful implementation of an approach to develop SDAMs described by Fritz and others [1] in the Arid West of the United States. We found that biological indicators were particularly useful because of their ability to reflect long-term patterns in streamflow duration exhibited at a site. Our final SDAM was more successful in distinguishing ephemeral from intermittent reaches than perennial from intermittent, consistent with SDAMs developed in other regions. The most substantial limitation was the scarcity of non-perennial reaches with sufficiently documented flow regimes that could be used to calibrate an SDAM. Thanks to the oversight of a regional steering committee comprised of technical experts who require streamflow duration information in their management and monitoring programs, we were able to ensure that this SDAM would be accessible and easy to use by its target audience, despite the statistical complexity underlying its classifications. All data collected for this study and code used for analysis are available in Supplementary File S1.

## Supplementary Material

SI

## Figures and Tables

**Table 1.** Indicators evaluated in the present study. Indicators with “NM” in the Origin column were measured following the NM method protocol [34], and indicators marked with “PNW” were measured following the PNW protocol [27]; other indicators were measured with protocols developed for this study, which are provided in Supplementary File S1, and come from sources reviewed in a study by McCune and Mazor [35]. Asterisks (*) indicate hydrologic indicators that are considered direct measures of water presence.

Indicator	Description	Origin
***Geomorphic Indicators***		
**Sinuosity**	Visual estimate of the curviness of the stream channel	NM
**Bankfull width**	Width of the channel at bankfull height	PNW
**Floodplain channel dimensions**	Visual estimate of the extent of channel entrenchment and connectivity to the floodplain	NM
**Particle size/stream substrate sorting**	Visual estimate of the extent of evidence of substrate sorting within the channel	NM
**In-channel structure/riffle pool sequence**	Visual estimate of the diversity and distinctiveness of riffles, pools, and other flow-based microhabitats	NM
**Sediment deposition on plants and debris**	Visual estimate of the extent of evidence of sediment deposition on plants and on debris within the floodplain	NM
***Hydrologic indicators***		
Surface and subsurface flow *	Estimates of the percent of the reach-length with surface and subsurface flow	PNW
Isolated pools *	Number of pools in the channel without any connection to flowing surface water	PNW
Water in channel *	Visual estimate of the extent of surface flow in the channel	NM
Seeps and springs *	Presence/absence of springs or seeps within one-half channel width of the channel	NM
**Hydric soils**	Presence/absence of hydric soils within the channel, measured at up to 3 locations	NM
Soil moisture and texture *	Extent of soil saturation and texture measured at three locations in the channel	
**Woody jams**	Number of woody jams within the channel	
***Biological indicators***		
**Live and dead algal cover**	Visual estimate of the percent of streambed covered by live or dead algal growth	
**Filamentous algal abundance**	Estimate of the overall abundance of filamentous algae within the channel	NM
**Stream shading**	Percent shade-providing cover above the streambed measured with a densiometer at three locations	
**Hydrophytic plant species**	Number of OBL or FACW-rated plants (as listed in [36]) growing within the channel or a half-channel width from the channel	PNW
**Fish**	Estimate of the overall abundance of fish (other than non-native mosquitofish) in the channel.	NM
**Aquatic invertebrates**	Abundance and richness of aquatic invertebrate families collected from the channel	PNW
**Aquatic invertebrates**	Estimate of the overall abundance of aquatic invertebrates within the channel	NM
**Amphibians**	Estimate of the overall abundance of amphibians within the channel	NM
**Mosses and liverworts**	Visual estimate of the percent of streambed and banks covered by live or dead bryophytes or liverworts	
**Differences in vegetation (riparian corridor)**	Visual estimate of the distinctiveness of vegetation in the riparian corridor compared to surrounding upland vegetation	NM
**Absence of upland rooted plants in the streambed**	Visual estimate of the extent of upland rooted plants growing within the streambed	NM
**Presence of iron-oxidizing fungi or bacteria**	Presence of oily sheens indicative of iron-oxidizing fungi or bacteria within the assessment reach	NM
**Presence of aquatic or semi-aquatic snakes**	Presence of aquatic or semi-aquatic snakes (e.g., most garter snake species) in the channel	PNW
**Geospatial**		
**Location and watershed characteristics**	Latitude, longitude, elevation, and watershed area (watershed area retrieved from StreamCat database [37])	
**Long-term normal precipitation and temperature**	30-year normal mean annual and monthly precipitation, and 30-y normal mean, maximum, and minimum annual temperature (PRISM climate data; [38]).	
**Soil type**	Landscape metrics related to soil (such as erodibility, hydraulic conductivity, and bulk density) calculated at the watershed and catchment scale (StreamCat database [37])	
**Geology**	Landscape metrics related to geology (such as geological nitrogen content in bedrock) calculated at the watershed and catchment scale (StreamCat database [37])	
**Ecoregion**	Level 2 and 3 ecoregions for the Western United States [39]	

**Table 2.** Metric screening criteria. Metrics had to meet the distribution criterion and at least one responsiveness criterion to be considered in further analysis.

Criterion		Definition
**Distribution Criterion**		
% dominance of most common value	<95%	Frequency of most common value (typically, zero) in the development dataset.
**Responsiveness criteria**		
PvIvE	F > 2	F-statistic in a comparison of values at perennial versus intermittent versus ephemeral reaches
EvALI	t > 2	t-statistic in a comparison of values at ephemeral versus at least intermittent reaches
PvNP_t	t > 2	t-statistic in a comparison of values at perennial versus non-perennial reaches
PvIwet_t	t > 2	t-statistic in a comparison of values at perennial versus flowing intermittent reaches
EvIdry_t	t > 2	t-statistic in a comparison of values at ephemeral versus dry intermittent reaches
rf_MDA	Top quartile	Mean decrease accuracy (MDA) in a random forest model to predict perennial, intermittent, or ephemeral streamflow duration class

**Table 3.** Number of sampled reaches by streamflow duration class and subregion. AZ: Arizona. CA: California. CO, WY, UT, and MT: Colorado, Wyoming, Utah, and Montana. NM and TX: New Mexico and Texas. NV: Nevada.

Subregion	Ephemeral	Intermittent	Perennial
AZ	12	8	6
AZ revisits	1	1	1
CA	3	9	5
CA revisits	1	1	1
CO, WY, UT, and MT	6	6	3
NM and TX	5	4	5
NV	4	7	6
NV revisits	0	3	2

**Table 4.** Indicator metric descriptions and screening criteria; only metrics that passed screening criteria are shown. The full list of metrics evaluated in this study are presented in Supplementary File S3. (NM) indicates metrics that are scored following the NM protocol [34]. % dom: percent dominance of most common value. PvIvE-F: F-statistic from an analysis of variance comparing values at perennial versus intermittent versus ephemeral reaches. EvALI-t: t-statistic from a comparison of mean values at ephemeral versus at least intermittent reaches. PvNP-t: t-statistic from a comparison of mean values at perennial versus non-perennial reaches. PvIwet-t: t-statistic from a comparison of mean values at perennial versus wet intermittent reaches. PvIdry-t: t-statistic from a comparison of mean values at ephemeral versus dry intermittent reaches. RF-MDA: Variable importance (as mean decrease accuracy) from a random forest model predicting perennial, intermittent, or ephemeral streamflow duration class. Black text indicates metric values that passed screening criteria, while gray text indicates metric values that did not pass screening criteria. To pass, a metric had to pass the % dominance criterion, plus at least one responsiveness criterion.

			Responsiveness Criteria					
			PvIvE	EvALI	PvNP	PvIwet	EvIdry	RF
Indicator	Description	% dom	F	t	t	t	t	MDA
***Biological indicators***								
**Invertebrate metrics**								
**bmiabund_score**	Aquatic invertebrate abundance score (NM)	40%	67.72	12.97	9.03	2.69	2.56	0.0067
**TotalAbundance**	Total aquatic invertebrate abundance	34%	32.63	10.21	5.85	2.64	2.03	0.0094
**Richness**	Total aquatic invertebrate richness	34%	37.63	10.72	6.14	2.53	2.11	0.0061
**mayfly_abundance**	Abundance of mayflies	49%	31.82	10.16	5.79	2.63	1.26	0.0049
**perennial_abundance**	Abundance of perennial indicator taxa	65%	16.05	6.26	4.33	2.55	1.00	0.0004
**perennial_taxa**	Richness of perennial indicator taxa	65%	19.02	7.49	4.61	2.37	1.00	0.0010
**perennial_live_abundance**	Abundance of live perennial indicator taxa	66%	15.54	6.05	4.29	2.58	1.00	0.0008
**EPT_abundance**	Ephemeroptera, Plecoptera, and Trichoptera (EPT) abundance	46%	33.09	9.26	6.13	3.41	1.02	0.0045
**EPT_taxa**	EPT richness	46%	37.65	10.33	6.65	3.29	1.59	0.0061
**EPT_relabd**	EPT relative abundance	46%	34.13	10.66	6.40	2.46	1.32	0.0049
**EPT_reltaxa**	EPT relative richness	46%	34.64	10.96	6.39	2.63	1.49	0.0056
**GOLD_relabd**	Gastropoda, Oligochaeta, and Diptera (GOLD) relative abundance	43%	9.91	6.06	1.93	0.75	1.48	0.0008
**GOLD_reltaxa**	GOLD relative richness	43%	11.79	5.78	2.73	0.31	1.41	0.0027
**OCH_relabd**	Odonata, Coleoptera, and Heteroptera (OCH) relative abundance	56%	2.38	2.03	0.19	1.27	2.27	0.0004
**OCH_reltaxa**	OCH relative richness	55%	5.10	3.17	0.16	1.40	2.63	0.0004
**GOLDOCH_relabd**	GOLD + OCH relative abundance	38%	11.32	4.94	1.31	1.65	2.50	0.0017
**GOLDOCH_reltaxa**	GOLD + OCH relative richness	38%	14.34	5.57	1.94	1.33	2.68	0.0018
**Noninsect_abundance**	Non-insect abundance	67%	4.01	4.36	1.57	1.08	2.01	0.0001
**Noninsect_taxa**	Non-insect richness	67%	5.95	5.31	2.00	0.96	2.00	0.0004
**Noninsect_relabund**	Non-insect relative abundance	67%	3.55	4.05	0.57	0.02	2.06	−0.0002
**Noninsect_reltaxa**	Non-insect relative richness	67%	4.82	4.93	0.77	0.49	2.19	0.0002
**Vertebrate metrics**								
**fishabund_score2**	Fish abundance score (NM) (excluding mosquitofish)	78%	6.16	5.63	2.12	0.73	2.03	0.0001
**frogvoc_score**	Presence of frog vocalizations	93%	3.19	1.47	1.93	2.31	0.28	0.0005
**vert_score**	Presence of aquatic vertebrates	84%	3.29	2.31	0.93	1.77	0.89	0.0002
**vertvoc_score**	Presence of aquatic vertebrates, including frog vocalizations	81%	3.14	2.04	1.01	1.95	0.50	−0.0003
**vert_sumscore**	Total number of aquatic vertebrate types detected	84%	3.73	2.47	1.19	1.88	1.06	0.0005
**vertvoc_sumscore**	Total number of aquatic vertebrate types detected, including frog vocalizations	81%	5.34	2.69	1.86	2.53	0.98	0.0012
**Algal metrics**								
**algabund_score**	Algal abundance score (NM)	49%	24.96	8.93	5.06	1.28	1.81	0.0053
**alglive_cover_score**	Live algal cover on the streambed	51%	24.38	7.89	5.42	1.61	1.53	0.0043
**algdead_noupstream_cover_score**	Dead algal cover on the streambed, excluding mats deposited from upstream sources	81%	1.53	2.11	0.13	0.04	1.51	0.0006
**alglivedead_cover_score**	Live or dead algal cover on the streambed	46%	21.86	7.39	5.03	1.61	1.66	0.0017
**Plant metrics**								
**vegdiff_score**	Difference in vegetation score (NM)	28%	18.42	5.87	4.80	1.51	1.11	0.0019
**rootedplants_score**	Uplant rooted plants in streambed score (NM)	44%	14.92	4.71	4.15	0.63	0.80	0.0007
**hydrophytes_present_noflag**	Numer of hydrophytic plant species observed (FACW and OBL)	37%	24.29	8.13	5.10	1.85	3.02	0.0042
**moss_cover_score**	Streamer moss cover in the channel	80%	7.34	4.95	2.81	1.86	1.46	0.0000
**liverwort_cover_score**	Liverwort cover in the channel	91%	2.04	3.32	0.20	0.77	1.00	0.0000
**PctShading**	Percent stream shading	19%	10.12	5.41	2.69	1.02	3.32	0.0035
**Other biological metrics**								
**iofb_score**	Presence of iron-oxidizing fungi or bacteria	82%	6.40	5.59	2.30	0.86	1.45	−0.0001
***Geomorphological indicators***								
**sinuosity_score**	Sinuosity score (NM)	38%	1.70	1.72	0.27	0.51	2.15	−0.0003
**riffpoolseq_score**	Riffle-pool sequence score (NM)	28%	11.25	4.10	3.93	2.09	1.93	0.0003
**substratesorting_score**	Substrate sorting score (NM)	29%	7.34	3.19	3.30	1.23	0.90	0.0001
**BankWidthMean**	Mean bankfull width	5%	10.16	3.13	1.19	0.43	3.31	0.0029
***Hydrologic indicators***								
**waterinchannel_score**	Water in channel score (NM)	53%	68.24	10.55	10.27	1.30	2.77	0.0145
**hydric_score**	Presence of hydric soils	78%	12.78	6.51	3.82	2.49	1.83	0.0004
**pctsurfaceflow**	Percent surface flow in channel	59%	66.26	11.33	10.72	1.41	0.82	0.0127
**pctsubsurfaceflow**	Percent surface or subsurface flow in channel	60%	59.57	10.53	9.22	0.55	1.53	0.0105
**SoilMoist_MaxScore**	Maximum soil moisture	69%	58.23	8.86	8.05	0.00	2.77	0.0133

**Table 5.** Performance of final methods and calibrated models. SDAM AW: the final streamflow duration assessment method for the AW, including all modifications and use of single indicators. NM: the New Mexico method [34]. PNW: the Pacific Northwest method [27]. Base: Biological metrics, geomorphological metrics, and hydrologic metrics that did not directly measure the presence of water. GIS: Geospatial metrics. H2O: Hydrological metrics that directly measure the presence of surface water. Accuracy measures are proportion of correct classifications. Cal: Calibration data. Val: Validation data. PvIvE: Proportion of reaches correctly classified as perennial, intermittent, or ephemeral. EvALI: Proportion of reaches correctly classified as ephemeral or at least intermittent reaches. PvIwet: Proportion of flowing reaches correctly classified as perennial or intermittent. EvIdry: Proportion of dry reaches correctly classified as ephemeral or intermittent. EnotP: Proportion of ephemeral reaches correctly not classified as perennial. PnotE: Proportion of perennial reaches correctly not classified as ephemeral. PvNP: Proportion of reaches correctly classified as perennial or non-perennial. Repeatability: Proportion of revisited reaches with the same classification for each visit. Repeatability was not assessed for calibration and validation data separately. Because the NM and PNW methods were developed with independent data, the division of calibration and validation data is not applicable. The highlighted rows marked with ^a^ indicate the performance measures for the final method. The highlighted rows marked with ^b^ indicate the model that was selected for refinement to create the final model.

				Accuracy								
Method	Dataset			PvIvE	EvALI	PvIwet	EvIdry	EnotP	PnotE	PvNP	Repeatability	
*Final methods*												
SDAM AW	Cal			0.56	0.81	0.50	0.67	0.95	0.96	0.72	0.50	^a^
	Val			0.65	0.88	0.40	0.75	1.00	1.00	0.76		
NM				0.62	0.82	0.60	0.67	1.00	0.96	0.79	0.42	
PNW				0.60	0.83	0.49	0.74	1.00	0.96	0.75	0.42	
*Calibrated models*												
Indicators	Stratification	Model type	Dataset									
Base	No	Random Forest	Cal	0.64	0.85	0.56	0.76	0.96	1.00	0.78	0.33	^b^
			Val	0.35	0.65	0.25	0.38	0.80	1.00	0.65		
		Single Tree	Cal	0.76	0.86	0.76	0.79	1.00	0.95	0.89	0.58	
			Val	0.41	0.65	0.25	0.46	1.00	1.00	0.76		
	Yes	Random Forest	Cal	0.52	0.81	0.32	0.67	1.00	1.00	0.71	0.37	
			Val	0.60	0.85	0.64	0.50	0.75	1.00	0.70		
		Single Tree	Cal	0.78	0.88	0.74	0.81	1.00	1.00	0.90	0.58	
			Val	0.35	0.75	0.36	0.33	1.00	1.00	0.60		
GIS	No	Random Forest	Cal	0.65	0.83	0.57	0.71	1.00	0.95	0.80	0.58	
			Val	0.56	0.88	0.56	0.57	1.00	1.00	0.69		
		Single Tree	Cal	0.79	0.85	0.86	0.71	0.88	1.00	0.90	0.50	
			Val	0.50	0.88	0.33	0.71	1.00	1.00	0.63		
	Yes	Random Forest	Cal	0.56	0.77	0.53	0.58	0.96	0.94	0.76	0.43	
			Val	0.65	0.82	0.63	0.63	1.00	1.00	0.82		
		Single Tree	Cal	0.71	0.84	0.75	0.70	1.00	1.00	0.87	0.63	
			Val	0.65	0.82	0.63	0.63	1.00	1.00	0.82		
H2O	No	Random Forest	Cal	0.64	0.85	0.63	0.69	0.96	1.00	0.78	0.58	
			Val	0.47	0.76	0.29	0.60	1.00	1.00	0.71		
		Single Tree	Cal	0.78	0.90	0.79	0.81	0.96	1.00	0.86	0.58	
			Val	0.59	0.76	0.57	0.60	1.00	1.00	0.82		
	Yes	Random Forest	Cal	0.60	0.78	0.65	0.58	1.00	1.00	0.82	0.58	
			Val	0.71	0.94	0.50	0.89	1.00	1.00	0.76		
		Single Tree	Cal	0.74	0.89	0.70	0.82	1.00	1.00	0.85	0.62	
			Val	0.82	0.94	0.75	0.89	1.00	1.00	0.88		
H20 + GIS	No	Random Forest	Cal	0.66	0.87	0.61	0.71	1.00	1.00	0.79	0.58	
			Val	0.47	0.65	0.63	0.43	1.00	1.00	0.82		
		Single Tree	Cal	0.81	0.93	0.78	0.85	1.00	1.00	0.89	0.75	
			Val	0.35	0.71	0.25	0.57	0.80	1.00	0.59		
	Yes	Random Forest	Cal	0.61	0.86	0.52	0.71	1.00	1.00	0.74	0.57	
			Val	0.62	0.86	0.55	0.70	1.00	1.00	0.76		
		Single Tree	Cal	0.74	0.88	0.76	0.77	0.87	1.00	0.82	0.78	
			Val	0.48	0.71	0.55	0.40	1.00	1.00	0.76		

**Table 6.** Classification table for the beta SDAM AW. Blank entries in a column mean that the state of a certain indicator does not change the outcome. For example, if a reach has no hydrophytes, few aquatic invertebrates, and EPT taxa are present, it will be classified as *At least intermittent*, regardless of whether algae or fish are observed.

1. Hydrophytic Plant Species	2. Aquatic Invertebrates	3. EPT Taxa	4. Algae	5. Single Indicators • Fish Present • Algal Cover ≥ 10%	Classification
None	None	Absent	Absent	Absent	**Ephemeral**
				Present	**At least intermittent**
			Present	Absent	**Need more information**
				Present	**At least intermittent**
	Few (1-19)	Absent	Absent	Absent	**Need more information**
				Present	**At least intermittent**
			Present	Absent	**Need more information**
				Present	**At least intermittent**
		Present			**At least intermittent**
	Many (20+)	Absent	Absent	Absent	**Need more information**
				Present	**At least intermittent**
			Present	Absent	**Need more information**
				Present	**At least intermittent**
		Present			**At least intermittent**
Few (1-2)	None	Absent	Absent	Absent	**Need more information**
				Present	**At least intermittent**
			Present		**At least intermittent**
	Few (1-19)	Absent	Absent		**Intermittent**
			Present		**At least intermittent**
		Present			**At least intermittent**
	Many (20+)	Absent	Absent		**Intermittent**
			Present		**At least intermittent**
		Present	Absent		**At least intermittent**
			Present		**Intermittent**
Many (3+)	None	Absent	Absent	Absent	**Need more information**
				Present	**At least intermittent**
			Present		**At least intermittent**
	Few (1-19)	Absent			**At least intermittent**
		Present			**Perennial**
	Many (20+)	Absent			**At least intermittent**
		Present			**Perennial**

**Table 7.** Number of reach-visits classified by the beta SDAM AW. Bold values are number of reach visits with agreement between the true streamflow duration class and the beta SDAM AW classification. This table shows results for both calibration and validation reaches.

	True Streamflow Duration Class				
	Ephemeral		Intermittent		Perennial
Observed Flow during Sampling	Dry	Flowing	Dry	Flowing	Flowing
Classification by beta SDAM AW					
Initial reach visit					
- Ephemeral	**24**	**0**	7	1	0
- **Intermittent**	0	0	**2**	**7**	4
- **At least intermittent**	0	0	**2**	**6**	**7**
- Perennial	1	0	1	4	**14**
- Need more information	3	2	2	2	0
Second reach visit					
- Ephemeral	**1**	**0**	0	0	0
- Intermittent	0	0	**1**	**3**	3
- At least intermittent	0	0	**1**	**0**	**1**
- Perennial	0	0	0	2	**0**
- Need more information	0	0	0	0	0

**Table 8.** Comparison of classifications by the beta SDAM with other methods used in the AW. The New Mexico method allows “gray-zone” tentative classifications when reaches have intermediate scores. Concordant classifications are shown in bold. For revisited reaches, classifications of both visits are included.

	Classification from the beta SDAM AW				
Method	Ephemeral	Intermittent	Perennial	At Least Intermittent	Need More Information
New Mexico					
Ephemeral	**28**	0	0	2	2
Intermittent (tentative)	3	**1**	0	0	2
Intermittent	2	**5**	2	**5**	5
Perennial (tentative)	0	2	**2**	**4**	0
Perennial	0	12	**18**	**6**	0
Pacific Northwest					
Ephemeral	**30**	0	0	2	1
Intermittent	3	**13**	10	**11**	8
Perennial	0	7	**12**	4	0

## Data Availability

All data collected for this study and code used for analysis are available in Supplementary File S1.

## Acronyms and Abbreviations

ALI: At least intermittent
AW: Arid West
AZ: Arizona
CA: California
CO: Colorado
Corps: United States Army Corps of Engineers
EPT: Ephemeroptera, Plecoptera, and Trichoptera taxa
EvALI: Ephemeral versus at least intermittent
FACW: Facultative-Wet wetland plant indicator status
GIS: Geographic Information System
GOLD: Gastropoda, Oligochaeta, and Diptera taxa
GOLDOCH: Gastropoda, Oligochaeta, Diptera, Odonata, Coleoptera, and Heteroptera taxa
H2O: Direct measures of water presence
MDA: Mean decrease accuracy (a measure of variable importance in random forest models)
MDS: Multidimensional scaling
MT: Montana
NHD: National Hydrography Dataset
NM: New Mexico
NMI: Need more information
NV: Nevada
OBL: Obligate wetland plant indicator status
OCH: Odonata, Coleoptera, and Heteroptera taxa
PNW: Pacific Northwest
PRISM: Parameter-elevation Regressions on Independent Slope Models (a repository of modeled climate data)
PvIvE: Perennial versus intermittent versus ephemeral
PvNP: Perennial versus non-perennial
RSC: Regional Steering Committee
SDAM: Streamflow duration assessment method
SDAM AW: Streamflow duration assessment method for the Arid West
SDAM NM: Streamflow duration assessment method for New Mexico
SDAM PNW: Streamflow duration assessment method for the Pacific Northwest
STIC: Stream Temperature, Intermittence, and Conductivity logger
TX: Texas
USEPA: United States Environmental Protection Agency
USGS: United States Geological Survey
UT: Utah
WY: Wyoming

## References

[1] Fritz KM; Nadeau T-L; Kelso JE; Beck WS; Mazor RD; Harrington RA; Topping BJ Classifying Streamflow Duration: The Scientific Basis and an Operational Framework for Method Development. Water 2020, 12, 2545. 10.3390/w12092545 PMC7592706 33133647

[2] Granato GE; Ries III KG; Steeves PA Compilation of Streamflow Statistics Calculated from Daily Mean Streamflow Data Collected during Water Years 1901–2015 for Selected U.S. Geological Survey Streamgages; U.S. Geological Survey Open-File Report 2017-1108; U.S. Geological Survey: Reston, VA, USA, 2017; p. 17. Available online: https://pubs.er.usgs.gov/publication/ofr20171108 (accessed on 20 November 2021).

[3] Zimmer MA; Kaiser KE; Blaszczak JR; Zipper SC; Hammond JC; Fritz KM; Costigan KH; Hosen J; Godsey SE; Allen GH; Zero or Not? Causes and Consequences of Zero-flow Stream Gage Readings. WIREs Water 2020, 7. 10.1002/wat2.1436 PMC7425737 32802326

[4] Anning DW; Parker JTC Predictive Models of the Hydrological Regime of Unregulated Streams in Arizona; U.S. Geological Survey Open-File Report; US Geological Survey: Tucson, AZ, USA, 2009; p. 33. Available online: https://pubs.usgs.gov/of/2009/1269/ (accessed on 20 November 2021).

[5] Reynolds LV; Shafroth PB; LeRoy Poff N Modeled Intermittency Risk for Small Streams in the Upper Colorado River Basin under Climate Change. J. Hydrol 2015, 523, 768–780.

[6] Sando R; Blasch KW Predicting Alpine Headwater Stream Intermittency: A Case Study in the Northern Rocky Mountains. Ecohydrol. Hydrobiol 2015, 15, 68–80.

[7] Lane BA; Dahlke HE; Pasternack GB; Sandoval-Solis S Revealing the Diversity of Natural Hydrologic Regimes in California with Relevance for Environmental Flows Applications. J. Am. Water Resour. Assoc 2017, 53, 411–430.

[8] Merritt A; Lane B; Hawkins C Classification and Prediction of Natural Streamflow Regimes in Arid Regions of the USA. Water 2021, 13, 380.

[9] Busch MH; Costigan KH; Fritz KM; Datry T; Krabbenhoft CA; Hammond JC; Zimmer M; Olden JD; Burrows RM; Dodds WK; What’s in a Name? Patterns, Trends, and Suggestions for Defining Non-Perennial Rivers and Streams. Water 2020, 12, 1980. 33274073 PMC7707420

[10] Costigan KH; Jaeger KL; Goss CW; Fritz KM; Goebel PC Understanding Controls on Flow Permanence in Intermittent Rivers to Aid Ecological Research: Integrating Meteorology, Geology and Land Cover: Integrating Science to Understand Flow Intermittence. Ecohydrology 2016, 9, 1141–1153.

[11] Leasure DR; Magoulick DD; Longing SD Natural Flow Regimes of the Ozark-Ouachita Interior Highlands Region. River Res. Applic 2016, 32, 18–35.

[12] Texas Commission on Environmental Quality. Chapter 307—Texas Surface Water Quality Standards; Rule Project No. 2016-002-307-OW; Texas Commission on Environmental Quality: Austin, TX, USA, 2018; p. 212.

[13] Poff NL; Ward JV Implications of Streamflow Variability and Predictability for Lotic Community Structure: A Regional Analysis of Streamflow Patterns. Can. J. Fish. Aquat. Sci 1989, 46, 1805–1818.

[14] Winter TC; Harvey JW; Franke OL; Alley WM Groundwater and Surface Water: A Single Resource; US Geological Survey: Denver, CO, USA, 1998; p. 79.

[15] Chadwick MA; Huryn AD Role of Habitat in Determining Macroinvertebrate Production in an Intermittent-Stream System. Freshw. Biol 2007, 52, 240–251.

[16] Fritz KM; Johnson BR; Walters DM Physical Indicators of Hydrologic Permanence in Forested Headwater Streams. J. N. Am. Benthol. Soc 2008, 27, 690–704.

[17] Austin BJ; Strauss EA Nitrification and Denitrification Response to Varying Periods of Desiccation and Inundation in a Western Kansas Stream. Hydrobiologia 2011, 658, 183–195.

[18] Datry T Benthic and Hyporheic Invertebrate Assemblages along a Flow Intermittence Gradient: Effects of Duration of Dry Events: River Drying and Temporary River Invertebrates. Freshw. Biol 2012, 57, 563–574.

[19] Schriever TA; Bogan MT; Boersma KS; Cañedo-Argüelles M; Jaeger KL; Olden JD; Lytle DA Hydrology Shapes Taxonomic and Functional Structure of Desert Stream Invertebrate Communities. Freshw. Sci 2015, 34, 399–409.

[20] Wipfli MS; Richardson JS; Naiman RJ Ecological Linkages Between Headwaters and Downstream Ecosystems: Transport of Organic Matter, Invertebrates, and Wood Down Headwater Channels1: Ecological Linkages Between Headwaters and Downstream Ecosystems. JAWRA J. Am. Water Resour. Assoc 2007, 43, 72–85.

[21] Nadeau T-L; Rains MC Hydrological Connectivity Between Headwater Streams and Downstream Waters: How Science Can Inform Policy. JAWRA J. Am. Water Resour. Assoc 2007, 43, 118–133.

[22] Stubbington R; Bogan MT; Bonada N; Boulton AJ; Datry T; Leigh C; Vander Vorste R The Biota of Intermittent Rivers and Ephemeral Streams: Aquatic Invertebrates. In Intermittent Rivers and Ephemeral Streams; Elsevier: Amsterdam, The Netherlands, 2017; pp. 217–243. ISBN 978-0-12-803835-2.

[23] Steward AL; Langhans SD; Corti R; Datry T The Biota of Intermittent Rivers and Ephemeral Streams: Terrestrial and Semiaquatic Invertebrates. In Intermittent Rivers and Ephemeral Streams; Elsevier: Amsterdam, The Netherlands, 2017; p. 245. ISBN 978-0-12-803835-2.

[24] Leigh C; Datry T Drying as a Primary Hydrological Determinant of Biodiversity in River Systems: A Broad-Scale Analysis. Ecography 2017, 40, 487–499.

[25] Nadeau T-L; Leibowitz SG; Wigington PJ; Ebersole JL; Fritz KM; Coulombe RA; Comeleo RL; Blocksom KA Validation of Rapid Assessment Methods to Determine Streamflow Duration Classes in the Pacific Northwest, USA. Environ. Manag 2015, 56, 34–53. 10.1007/s00267-015-0466-4 25931296

[26] U.S. Army Corps of Engineers; U.S. Environmental Protection Agency. Notice of Availability of the Beta Streamflow Duration Assessment Method for the Arid West; U.S. Army Corps of Engineers: Washington, DC, USA, 2021. Available online: https://www.epa.gov/sites/production/files/2021-03/documents/joint_spd_pn_sdam_11mar21_final_508.pdf (accessed on 20 November 2021).

[27] Nadeau T-L Streamflow Duration Assessment Method for the Pacific Northwest; EPA 910-K-14-001; U.S. Environmental Protection Agency: Washington, DC, USA, 2015; p. 36. Available online: https://www.epa.gov/sites/default/files/2016-01/documents/streamflow_duration_assessment_method_pacific_northwest_2015.pdf (accessed on 20 November 2021).

[28] U.S. Army Corps of Engineers. Regional Supplement to the Corps of Engineers Wetland Delineation Manual: Arid West Region (Version 2.0); Regional Supplements to the Corps of Engineers Wetland Delineation Manual; U.S. Army Engineer Research and Development Center: Vicksburg, MS, USA; Army Engineer Research and Development Center: Hanover, NH, USA, 2008; p. 135.

[29] Gasith A; Resh VH Streams in Mediterranean Climate Regions: Abiotic Influences and Biotic Responses to Predictable Seasonal Events. Annu. Rev. Ecol. Syst 1999, 30, 51–81.

[30] Goodrich DC; Kepner WG; Levick LR; Wigington PJ Southwestern Intermittent and Ephemeral Stream Connectivity. J. Am. Water Resour. Assoc 2018, 54, 400–422.

[31] Messager ML; Lehner B; Cockburn C; Lamouroux N; Pella H; Snelder T; Tockner K; Trautmann T; Watt C; Datry T Global Prevalence of Non-Perennial Rivers and Streams. Nature 2021, 594, 391–397. 34135525 10.1038/s41586-021-03565-5

[32] Sleeter BM; Wilson TS; Soulard CE; Liu J Estimation of Late Twentieth Century Land-Cover Change in California. Environ. Monit. Assess 2011, 173, 251–266. 20217217 10.1007/s10661-010-1385-8

[33] Mackun P Fast Growth in the Desert Southwest Continues. In America Counts: Stories Behind the Numbers US Census Bureau; 2019. Available online: https://www.census.gov/library/stories/2019/02/fast-growth-in-desert-southwest-continues.html (accessed on 20 November 2021).

[34] New Mexico Environment Department (NMED). Hydrology Protocol for the Determination of Uses Supported by Ephemeral, Intermittent, and Perennial Waters; Surface Water Quality Bureau, New Mexico Environment Department: Albuquerque, NM, USA, 2011; p. 35.

[35] McCune K; Mazor RD Review of Flow Duration Methods and Indicators of Flow Duration in the Scientific Literature: Arid Southwest; SCCWRP Technical Report 1063; Southern California Coastal Water Research Project: Costa Mesa, CA, USA, 2019; p. 90. Available online: https://ftp.sccwrp.org/pub/download/DOCUMENTS/TechnicalReports/1063_FlowMethodsReview.pdf (accessed on 20 November 2021).

[36] Lichvar RW; Banks DL; Kirchner WN; Melvin NC The National Wetland Plant List: 2016 Wetland Ratings. Phytoneutron 2016, 30, 1–17.

[37] Hill RA; Weber MH; Leibowitz SG; Olsen AR; Thornbrugh DJ The Stream-Catchment (StreamCat) Dataset: A Database of Watershed Metrics for the Conterminous United States. J. Am. Water Resour. Assoc 2016, 52, 120–128.

[38] Hart E; Bell K Prism: Access Data from the Oregon State Prism Climate Project; R Package Version 0.0.6; 2015. Available online: https://www.researchgate.net/publication/308963991_prism_Access_data_from_the_Oregon_State_Prism_climate_project (accessed on 20 November 2021).

[39] Omernik JM; Griffith GE Ecoregions of the Conterminous United States: Evolution of a Hierarchical Spatial Framework. Environ. Manag 2014, 54, 1249–1266. 10.1007/s00267-014-0364-1 25223620

[40] Jaeger KL; Olden JD Electrical Resistance Sensor Arrays as a Means to Quantify Longitudinal Connectivity of Rivers. River Res. Applic 2012, 28, 1843–1852.

[41] Chapin TP; Todd AS; Zeigler MP Robust, Low-Cost Data Loggers for Stream Temperature, Flow Intermittency, and Relative Conductivity Monitoring. Water Resour. Res 2014, 50, 6542–6548.

[42] McKay L; Bondelid T; Dewald T; Johnson J; Moore R; Rea A NHDPlus Version 2: User Guide; U.S. Geological Survey: Reston, VA, USA, 2014; p. 173.

[43] Oksanen J; Blanchet FG; Friendly M; Kindt R; Legendre P; McGlinn D; Minchin PR; O’Hara RB; Simpson GL; Solymos P; Vegan: Community Ecology Package; R Package Version 2.5-6; 2019. Available online: https://CRAN.R-project.org/package=vegan (accessed on 20 November 2021).

[44] Stoddard JL; Herlihy AT; Peck DV; Hughes RM; Whittier TR; Tarquinio E A Process for Creating Multimetric Indices for Large-Scale Aquatic Surveys. J. N. Am. Benthol. Soc 2008, 27, 878–891.

[45] Hawkins CP; Cao Y; Roper B Method of Predicting Reference Condition Biota Affects the Performance and Interpretation of Ecological Indices. Freshw. Biol 2010, 55, 1066–1085.

[46] Cao Y; Hawkins CP The Comparability of Bioassessments: A Review of Conceptual and Methodological Issues. J. N. Am. Benthol. Soc 2011, 30, 680–701.

[47] Mazor RD; Rehn AC; Ode PR; Engeln M; Schiff KC; Stein ED; Gillett DJ; Herbst DB; Hawkins CP Bioassessment in Complex Environments: Designing an Index for Consistent Meaning in Different Settings. Freshw. Sci 2016, 35, 249–271.

[48] Liaw A; Wiener M Classification and Regression by RandomForest. R News 2002, 2, 18–22.

[49] Kuhn M Caret: Classification and Regression Training; R Package Version 6.0-86; 2020. Available online: https://CRAN.R-project.org/package=caret (accessed on 20 November 2021).

[50] Therneau T; Atkinson B Rpart: Recursive Partitioning and Regression Trees; R Package Version 4.1-15; 2019. Available online: https://CRAN.R-project.org/package=rpart (accessed on 20 November 2021).

[51] Topping BJD; Nadeau T-L; Turaski MR Oregon Streamflow Duration Assessment Method—Interm Version; U.S. Environmental Protection Agency: Portland, OR, USA, 2009; p. 60. Available online: https://www.epa.gov/sites/production/files/201601/documents/streamflow_duration_assessment_method_oregon_interim_2009.pdf (accessed on 20 November 2021).

[52] Dorney J; Russell P North Carolina Division of Water Quality Methodology for Identification of Intermittent and Perennial Streams and Their Origins. In Wetland and Stream Rapid Assessments; Elsevier: Amsterdam, The Netherlands, 2018; pp. 273–279. ISBN 978-0-12-805091-0.

[53] Gallart F; Llorens P; Latron J; Cid N; Rieradevall M; Prat N Validating Alternative Methodologies to Estimate the Regime of Temporary Rivers When Flow Data Are Unavailable. Sci. Total. Environ 2016, 565, 1001–1010. 27251770 10.1016/j.scitotenv.2016.05.116

[54] Gallart F; Cid N; Latron J; Llorens P; Bonada N; Jeuffroy J; Jiménez-Argudo S-M; Vega R-M; Solà C; Soria M; TREHS: An Open-Access Software Tool for Investigating and Evaluating Temporary River Regimes as a First Step for Their Ecological Status Assessment. Sci. Total. Environ 2017, 607–608, 519–540. 10.1016/j.scitotenv.2017.06.209 28704676

[55] Ohio EPA. Field Evaluation Manual for Ohio’s Primary Headwater Habitat Streams; Version 3.0; Ohio EPA Division of Surface Water: Columbus, OH, USA, 2012; p. 117. Available online: https://epa.ohio.gov/portals/35/wqs/headwaters/PHWHManual_2012.pdf (accessed on 20 November 2021).

[56] Svec JR; Kolka RK; Stringer JW Defining Perennial, Intermittent, and Ephemeral Channels in Eastern Kentucky: Application to Forestry Best Management Practices. For. Ecol. Manag 2005, 214, 170–182.

[57] Fritz KM; Johnson BR; Walters DM Field Operations Manual for Assessing the Hydrologic Permanence and Ecological Condition of Headwater Streams; U.S. Environmental Protection Agency, Ed; Office of Research and Development: Washington, DC, USA, 2006; p. 151. Available online: https://cfpub.epa.gov/si/si_public_record_report.cfm?Lab=NERL&dirEntryId=159984 (accessed on 20 November 2021).

[58] Greenwell BM Pdp: An R Package for Constructing Partial Dependence Plots. R J. 2017, 9, 421–436.

[59] Fritz KM; Wenerick WR; Kostich MS A Validation Study of a Rapid Field-Based Rating System for Discriminating Among Flow Permanence Classes of Headwater Streams in South Carolina. Environ. Manag 2013, 52, 1286–1298. 10.1007/s00267-013-0158-x 24000112

[60] Mazor RD Bioassessment Survey of the Stormwater Monitoring Coalition: Workplan for the Years 2021 through 2015; Version 1.0; Southern California Coastal Water Research Project: Costa Mesa, CA, USA, 2021; p. 52. Available online: https://ftp.sccwrp.org/pub/download/DOCUMENTS/TechnicalReports/1174_SMCBioassessmentWorkplan.pdf (accessed on 20 November 2021).

[61] Bêche LA; Resh VH Biological Traits of Benthic Macroinvertebrates in California Mediterranean-Climate Streams: Long-Term Annual Variability and Trait Diversity Patterns. Fundam. Appl. Limnol 2007, 169, 1–23.

[62] Bogan MT; Boersma KS; Lytle DA Flow Intermittency Alters Longitudinal Patterns of Invertebrate Diversity and Assemblage Composition in an Arid-Land Stream Network: Intermittent Flow Alters Longitudinal Patterns. Freshw. Biol 2013, 58, 1016–1028.

[63] Boersma KS; Dee LE; Miller SJ; Bogan MT; Lytle DA; Gitelman AI Linking Multidimensional Functional Diversity to Quantitative Methods: A Graphical Hypothesis-Evaluation Framework. Ecology 2016, 97, 583–593. 27197386 10.1890/15-0688

[64] Lawrence JE; Lunde KB; Mazor RD; Bêche LA; McElravy EP; Resh VH Long-Term Macroinvertebrate Responses to Climate Change: Implications for Biological Assessment in Mediterranean-Climate Streams. J. N. Am. Benthol. Soc 2010, 29, 1424–1440.

[65] Mazor RD; Stein ED; Ode PR; Schiff K Integrating Intermittent Streams into Watershed Assessments: Applicability of an Index of Biotic Integrity. Freshw. Sci 2014, 33, 459–474.

[66] Boyle TP; Fraleigh HD Natural and Anthropogenic Factors Affecting the Structure of the Benthic Macroinvertebrate Community in an Effluent-Dominated Reach of the Santa Cruz River, AZ. Ecol. Indic 2003, 3, 93–117.

[67] Halaburka BJ; Lawrence JE; Bischel HN; Hsiao J; Plumlee MH; Resh VH; Luthy RG Economic and Ecological Costs and Benefits of Streamflow Augmentation Using Recycled Water in a California Coastal Stream. Environ. Sci. Technol 2013, 47, 10735–10743. 23688175 10.1021/es305011z

[68] Hamdhani H; Eppehimer DE; Bogan MT Release of Treated Effluent into Streams: A Global Review of Ecological Impacts with a Consideration of Its Potential Use for Environmental Flows. Freshw Biol 2020, 65, 1657–1670.

[69] Baker SC; Sharp HF Evaluation of the Recovery of a Polluted Urban Stream Using the Ephemeroptera-Plecoptera-Trichoptera Index. J. Freshw. Ecol 1998, 13, 229–234.

[70] Mazor RD; Topping BJ; Nadeau T-L; Fritz KM; Kelso JE; Harrington RA; Beck WS; McCune K; Lowman H; Aaron A; User Manual for a Beta Streamflow Duration Assessment Method for the Arid West of the United States; Version 1.0; 2021; p. 83. Available online: https://www.epa.gov/sites/production/files/2021-03/documents/user_manual_beta_sdam_aw.pdf (accessed on 20 November 2021).

[71] U.S. Army Corps of Engineers. Antecedent Precipitation Tool (APT); 1.0.19; U.S. Army Corps of Engineers: Washington, DC, USA, 2020. Available online: https://github.com/jDeters-USACE/Antecedent-Precipitation-Tool/releases/latest (accessed on 20 November 2021).

[72] Freshwater Biomonitoring and Benthic Macroinvertebrates; Rosenberg DM; Resh VH (Eds.); Chapman & Hall: New York, NY, USA, 1993; ISBN 978-0-412-02251-7.

[73] Dias-Silva K; Vieira TB; de Matos TP; Juen L; Simião-Ferreira J; Hughes RM; De Marco Júnior P Measuring Stream Habitat Conditions: Can Remote Sensing Substitute for Field Data? Sci. Total. Environ 2021, 788, 147617. 34134352 10.1016/j.scitotenv.2021.147617

[74] Stromberg JC Influence of Stream Flow Regime and Temperature on Growth Rate of the Riparian Tree, Platanus wrightii, in Arizona: Influence of Stream Flow. Freshw. Biol 2001, 46, 227–239.

[75] Stromberg JC; Lite SJ; Marler R; Paradzick C; Shafroth PB; Shorrock D; White JM; White MS Altered Stream-Flow Regimes and Invasive Plant Species: The Tamarix Case. Glob. Ecol Biogeogr 2007, 16, 381–393.

[76] Caskey ST; Blaschak TS; Wohl E; Schnackenberg E; Merritt DM; Dwire KA Downstream Effects of Stream Flow Diversion on Channel Characteristics and Riparian Vegetation in the Colorado Rocky Mountains, USA: Effects of Flow Diversion in Colorado Rocky Mountains. Earth Surf. Process. Landf 2015, 40, 586–598.

[77] Reynolds LV; Shafroth PB Riparian Plant Composition along Hydrologic Gradients in a Dryland River Basin and Implications for a Warming Climate. Ecohydrology 2017, 10, e1864.

[78] Hill LW; Rice RM Converting from Brush to Grass Increases Water Yield in Southern California. J. Range Manag 1963, 16, 300.

[79] Bren LJ Effects of Slope Vegetation Removal on the Diurnal Variations of a Small Mountain Stream. Water Resour. Res 1997, 33, 321–331.

[80] Price K; Suski A; McGarvie J; Beasley B; Richardson JS Communities of Aquatic Insects of Old-Growth and Clearcut Coastal Headwater Streams of Varying Flow Persistence. Can. J. For. Res 2003, 33, 1416–1432.

[81] Clarke A; Mac Nally R; Bond N; Lake PS Flow Permanence Affects Aquatic Macroinvertebrate Diversity and Community Structure in Three Headwater Streams in a Forested Catchment. Can. J. Fish. Aquat. Sci 2010, 67, 1649–1657.

[82] Bonada N; Rieradevall M; Prat N; Resh VH Benthic Macroinvertebrate Assemblages and Macrohabitat Connectivity in Mediterranean-Climate Streams of Northern California. J. N. Am. Benthol. Soc 2006, 25, 32–43.

[83] Lusardi RA; Bogan MT; Moyle PB; Dahlgren RA Environment Shapes Invertebrate Assemblage Structure Differences between Volcanic Spring-Fed and Runoff Rivers in Northern California. Freshw. Sci 2016, 35, 1010–1022.

[84] Bramblett RG; Fausch KD Fishes, Macroinvertebrates, and Aquatic Habitats of the Purgatoire River in Pinon Canyon, Colorado. Southwest. Nat 1991, 36, 281.

[85] Mazzacano C; Black SH Using Aquatic Macroinvertebrates as Indicators of Streamflow Duration; The Xerces Society: Portland, OR, USA, 2008; p. 28.

[86] King AJ; Townsend SA; Douglas MM; Kennard MJ Implications of Water Extraction on the Low-Flow Hydrology and Ecology of Tropical Savannah Rivers: An Appraisal for Northern Australia. Freshw. Sci 2015, 34, 741–758.

[87] Miller MP; Brasher AMD Differences in Macroinvertebrate Community Structure in Streams and Rivers with Different Hydrologic Regimes in the Semi-Arid Colorado Plateau. River Syst. 2011, 19, 225–238.

[88] Straka M; Polášek M; Syrovátka V; Stubbington R; Zahrádková S; Němejcová D; Šikulová L; Řezničková P; Opatřilová L; Datry T; Recognition of Stream Drying Based on Benthic Macroinvertebrates: A New Tool in Central Europe. Ecological. Indic 2019, 106, 105486.

[89] Bogan MT Hurry up and Wait: Life Cycle and Distribution of an Intermittent Stream Specialist (*Mesocapnia arizonensis*). Freshw. Sci 2017, 36, 805–815.

[90] Cover MR; Seo JH; Resh VH Life History, Burrowing Behavior, and Distribution of Neohermes Filicornis (Megaloptera: Corydalidae), a Long-Lived Aquatic Insect in Intermittent Streams. West. N. Am. Nat 2015, 75, 474.

[91] Cañedo-Argüelles M; Bogan MT; Lytle DA; Prat N Are Chironomidae (Diptera) Good Indicators of Water Scarcity? Dryland Streams as a Case Study. Ecol. Indic 2016, 71, 155–162.

[92] De Jong GD; Canton SP; Lynch JS; Murphy M Aquatic Invertebrate and Vertebrate Communities of Ephemeral Stream Ecosystems In the Arid Southwestern United States. Southwest. Nat 2015, 60, 349–359.

[93] Benenati PL; Shannon JP; Blinn DW Desiccation and Recolonization of Phytobenthos in a Regulated Desert River: Colorado River at Lees Ferry, Arizona, USA. Regul. Rivers Res. Manag 1998, 14, 519–532.

[94] Robson BJ; Matthews TG; Lind PR; Thomas NA Pathways for Algal Recolonization in Seasonally-Flowing Streams. Freshw. Biol 2008, 53, 2385–2401.

[95] Chester ET; Robson BJ Do Recolonisation Processes in Intermittent Streams Have Sustained Effects on Benthic Algal Density and Assemblage Composition? Mar. Freshwater Res 2014, 65, 784.

[96] Sabater S; Timoner X; Bornette G; De Wilde M; Stromberg JC; Stella JC The Biota of Intermittent Rivers and Ephemeral Streams: Algae and Vascular Plants. In Intermittent Rivers and Ephemeral Streams; Elsevier: Amsterdam, The Netherlands, 2017; pp. 189–216. ISBN 978–0-12–803835-2.

[97] Wyatt KH; Rober AR; Schmidt N; Davison IR Effects of Desiccation and Rewetting on the Release and Decomposition of Dissolved Organic Carbon from Benthic Macroalgae. Freshw. Biol 2014, 59, 407–416.

[98] Sabater S; Timoner X; Borrego C; Acuña V Stream Biofilm Responses to Flow Intermittency: From Cells to Ecosystems. Front. Environ. Sci 2016, 4.

[99] Datry T; Corti R; Claret C; Philippe M Flow Intermittence Controls Leaf Litter Breakdown in a French Temporary Alluvial River: The “Drying Memory”. Aquat. Sci 2011, 73, 471–483.

[100] Timoner X; Acuña V; Von Schiller D; Sabater S Functional Responses of Stream Biofilms to Flow Cessation, Desiccation and Rewetting: Flow Intermittency Effects on Stream Biofilms. Freshw. Biol 2012, 57, 1565–1578.

[101] von Schiller D; Bernal S; Dahm CN; Marti E Nutrient and Organic Matter Dynamics in Intermittent Rivers and Ephemeral Streams. In Intermittent Rivers and Ephemeral Streams; Elsevier: Amsterdam, The Netherlands, 2017; pp. 135–160. ISBN 978-0-12-803835-2.

[102] Robson BJ Role of Residual Biofilm in the Recolonization of Rocky Intermittent Streams by Benthic Algae. Mar. Freshw. Res 2000, 51, 725.

[103] Bastow JL; Sabo JL; Finlay JC; Power ME A Basal Aquatic-Terrestrial Trophic Link in Rivers: Algal Subsidies via Shore-Dwelling Grasshoppers. Oecologia 2002, 131, 261–268. 28547694 10.1007/s00442-002-0879-7

[104] Nash J; Walters DE Public Engagement and Transparency in Regulation: A Field Guide to Regulatory Excellence; University of Pennsylvania Law School: Philadelphia, PA, USA, 2015; p. 43.

[105] Hampton SE; Anderson SS; Bagby SC; Gries C; Han X; Hart EM; Jones MB; Lenhardt WC; MacDonald A; Michener WK; The Tao of Open Science for Ecology. Ecosphere 2015, 6, art120.

[106] Beck MW; O’Hara C; Stewart Lowndes JS; Mazor RD; Theroux S; Gillett DJ; Lane B; Gearheart G The Importance of Open Science for Biological Assessment of Aquatic Environments. Peer J. 2020, 8, e9539. 32742805 10.7717/peerj.9539 PMC7377246

[107] Cutler DR; Edwards TC; Beard KH; Cutler A; Hess KT; Gibson J; Lawler JJ Random Forests for Classification in Ecology. Ecology 2007, 88, 2783–2792. 18051647 10.1890/07-0539.1

[108] Booker DJ; Snelder TH Comparing Methods for Estimating Flow Duration Curves at Ungauged Sites. J. Hydrol 2012, 434–435, 78–94.

[109] Petkovic D; Alavi A; Cai D; Wong M Random Forest Model and Sample Explainer for Non-Experts in Machine Learning—Two Case Studies. In Pattern Recognition. ICPR International Workshops and Challenges; Del Bimbo A, Cucchiara R, Sclaroff S, Farinella GM, Mei T, Bertini M, Escalante HJ, Vezzani R, Eds.; Lecture Notes in Computer Science; Springer International Publishing: Cham, Switerland, 2021; Volume 12663, pp. 62–75. ISBN 978-3-030-68795-3.

[110] Hedman ER; Osterkamp WR Streamflow Characteristics Related to Channel Geometry of Streams in Western United States; Water-Supply Paper; US Geological Survey: Washington, DC, USA, 1982; p. 17.

[111] Malmon DV; Reneau SL; Katzman D; Lavine A; Lyman J Suspended Sediment Transport in an Ephemeral Stream Following Wildfire. J. Geophys. Res 2007, 112, F02006.

[112] Esselman PC; Opperman JJ Overcoming Information Limitations for the Prescription of an Environmental Flow Regime for a Central American River. Ecol. Soc 2010, 15, 6.

[113] Cranney K; Tan P-L Old Knowledge in Freshwater: Why Traditional Ecological Knowledge Is Essential for Determining Environmental Flows in Water Plans. Australas. J. Nat. Resour. Law Policy 2011, 14, 71. Available online: https://search.informit.org/doi/abs/10.3316/ielapa.201200874 (accessed on 20 November 2021).

[114] Woodward E; Jackson S; Finn M; McTaggart PM. Utilising Indigenous Seasonal Knowledge to Understand Aquatic Resource Use and Inform Water Resource Management in Northern Australia: RESEARCH REPORT. Ecol. Manag. Restor 2012, 13, 58–64.

[115] Caruso BS GIS-Based Stream Classification in a Mountain Watershed for Jurisdictional Evaluation. J. Am. Water Resour. Assoc 2014, 50, 1304–1324.

[116] Chief K; Meadow A; Whyte K Engaging Southwestern Tribes in Sustainable Water Resources Topics and Management. Water 2016, 8, 350.

[117] Lines GC Health of Native Riparian Vegetation and Its Relation to Hydrologic Conditions along the Mojave River, Southern California; Water-Resources Investigations Report; US Geological Survey: Sacramento, CA, USA, 1999.

[118] King RS; Scoggins M; Porras A Stream Biodiversity Is Disproportionately Lost to Urbanization When Flow Permanence Declines: Evidence from Southwestern North America. Freshw. Sci 2016, 35, 340–352.

[119] Elbrecht V; Vamos EE; Meissner K; Aroviita J; Leese F Assessing Strengths and Weaknesses of DNA Metabarcoding-based Macroinvertebrate Identification for Routine Stream Monitoring. Methods Ecol. Evol 2017, 8, 1265–1275.

[120] Cordier T; Alonso-Sáez L; Apothéloz-Perret-Gentil L; Aylagas E; Bohan DA; Bouchez A; Chariton A; Creer S; Frühe L; Keck F; Ecosystems Monitoring Powered by Environmental Genomics: A Review of Current Strategies with an Implementation Roadmap. Mol. Ecol 2020, mec.15472. 10.1111/mec.15472 PMC8358956 32416615

[121] Joutsijoki H; Meissner K; Gabbouj M; Kiranyaz S; Raitoharju J; Ärje J; Kärkkäinen S; Tirronen V; Turpeinen T; Juhola M Evaluating the Performance of Artificial Neural Networks for the Classification of Freshwater Benthic Macroinvertebrates. Ecol. Inform 2014, 20, 1–12.

[122] Raitoharju J; Riabchenko E; Ahmad I; Iosifidis A; Gabbouj M; Kiranyaz S; Tirronen V; Ärje J; Kärkkäinen S; Meissner K Benchmark Database for Fine-Grained Image Classification of Benthic Macroinvertebrates. Image Vis. Comput 2018, 78, 73–83.

[123] Katz GL; Denslow MW; Stromberg JC The Goldilocks Effect: Intermittent Streams Sustain More Plant Species than Those with Perennial or Ephemeral Flow: Desert Riparian Diversity. Freshw. Biol 2012, 57, 467–480.

[124] Smith DM; Finch DM Riparian Trees and Aridland Streams of the Southwestern United States: An Assessment of the Past, Present, and Future. J. Arid. Environ 2016, 135, 120–131.

[125] Cubley ES; Bateman HL; Merritt DM; Cooper DJ Using Vegetation Guilds to Predict Bird Habitat Characteristics in Riparian Areas. Wetlands 2020, 40, 1843–1862.

[126] Kaplan NH; Sohrt E; Blume T; Weiler M Monitoring Ephemeral, Intermittent and Perennial Streamflow: A Dataset from 182 Sites in the Attert Catchment, Luxembourg. Earth Syst. Sci. Data 2019, 11, 1363–1374.

[127] Jiang D; Wang K The Role of Satellite-Based Remote Sensing in Improving Simulated Streamflow: A Review. Water 2019, 11, 1615.

[128] Ghaderpour E; Vujadinovic T; Hassan QK Application of the Least-Squares Wavelet Software in Hydrology: Athabasca River Basin. J. Hydrol. Reg. Stud 2021, 36, 100847.

[129] Dembélé M; Oriani F; Tumbulto J; Mariéthoz G; Schaefli B Gap-Filling of Daily Streamflow Time Series Using Direct Sampling in Various Hydroclimatic Settings. J. Hydrol 2019, 569, 573–586.

[130] Zimmerman JC; DeWald LE; Rowlands PG Vegetation Diversity in an Interconnected Ephemeral Riparian System of North-Central Arizona, USA. Biol. Conserv 1999, 90, 217–228.

[131] Moody EK; Sabo JL Dissimilarity in the Riparian Arthropod Communities along Surface Water Permanence Gradients in Aridland Streams. Ecohydrology 2017, 10, e1819.

