# Supplementary material for: Implementing an Operational Framework to Develop a Streamflow Duration Assessment Method: A Case Study from the Arid West United States
Source: Water (Basel). Author manuscript; Available in PMC 2022 Nov 22. (PMC8715911; doi:10.3390/w13223310)
Supplement: SI [file NIHMS1762665-supplement-SI.zip › water-1442778-SM.pdf]

Supplementary Materials

File S1. Sampling protocol, quality assurance, data and R code for the Beta SDAM AW 2
File S2. Determination of streamflow duration classes from hydrologic data 3
File S3. Indicator metrics evaluated in the development of the beta SDAM AW 6
File S4. Performance evaluation measures for all models across each sub-region. 21
File S5. Focus-area studies 29

File S1. Sampling protocol, quality assurance, data and R code for the Beta SDAM AW

The protocol may be downloaded here:

https://ftp.sccwrp.org/pub/download/PROJECTS/Attachment%201_Flow%20Duration%20Protocol_version2.zip

The quality assurance project plan may be downloaded here:

https://ftp.sccwrp.org/pub/download/SDAM/SDAM_for_Arid_SW_QAPP_version_2.0_Final_wattach_nosignatures.pdf

Data and R code used in analysis may be accessed here (doi:10.23719/1523371):

https://ftp.sccwrp.org/pub/download/PROJECTS/asw_fdam_FinalDataAndCode_Final.zip

File S2. Determination of streamflow duration classes from hydrologic data

Multiple hydrologic data sources were evaluated in order to classify candidate study reaches. Reaches were considered "acceptable" for the study if they had one source of hydrologic information, and "preferred" if they had two or more sources that provided consistent information. Reaches that had multiple conflicting sources that could not be reconciled were excluded from consideration. The sources of hydrologic data included:

Long-term continuous records, namely, data from the USGS stream gage network. USGS gage reaches were classified based on mean daily discharge at USGS gaging station within the AW region. Daily discharge data from gages within the ASW region active from 1959 to 2019 were downloaded with the *dataRetrieval* package in R [130]. Gages were considered for inclusion in the study if they had at least three years of continuous data and had a value for mean daily discharge for at least 99% of days over the period of record. Gages with more recent observations were prioritized for reach selection over older gage records, but due to the limited number of non-perennial gages with continuous and complete records older gages were considered and included in the study. The number of days within each water year with mean daily discharge equal to zero was used to calculate the percent of zero-flow days for period of record. Gages with <5% of the period of record with zero-flow days were considered perennial and the remainder of gages were considered non-perennial. The hydrograph for each non-perennial gage was then plotted and visually inspected. If discharge was greater than zero for several continuous days and the reach appeared to flow only in response to precipitation it was classified as ephemeral. If the reach was neither perennial nor ephemeral, it was classified as intermittent.

If it was unclear if gages were flowing only in response to precipitation, the *rnoaa* package [131] was used to download precipitation data from weather stations within 100 km of USGS gage locations. Then, cumulative precipitation within a water year was then plotted alongside discharge hydrographs. If a non-perennial gage appeared to be possibly perennial, or if a classification between intermittent and ephemeral was difficult to discern, the gage was flagged for review by the RSC. For example, if a reach showed different patterns in recent versus historical patterns, the hydrograph was flagged for RSC review. The RSC were provided with several feedback options for flagged USGS gages. Reviewers could suggest a likely classification, identify additional sources of information to confirm a classification, or recommend that a gage be excluded from the study because a definitive classification could not be determined. USGS stream gages were the primary source for perennial streams in the region.

Because the USGS stream gage network included few non-perennial streams, and because we preferred to have multiple sources of information whenever possible, we evaluated these additional sources of hydrologic data:

Short-term continuous records, such as data loggers (such as the STIC loggers mentioned above, or pressure transducers) and wildlife cameras deployed under other studies. Water presence data from these sources were plotted and visually inspected. Streams were classified as perennial if records indicated continuous flow in most years; as intermittent if extended dry periods were evident in most years; or as ephemeral if wet periods were typically lasted fewer than a few days.

Discontinuous records, such as field photos, aerial imagery, and field notes, were used to document the presence or absence of water during a reach-visit or a single point in time. Because of the sporadic nature of this data type, these data were only used to classify streams as intermittent in cases where

photos indicated the presence of surface water multiple weeks apart, as well as the absence of water in that same year. Field photographs and other discontinuous data sources were not used by themselves to classify streams as perennial or ephemeral, but could be used to corroborate classifications based on continuous records.

Published studies. Studies that identified intermittent or ephemeral stream-reaches were evaluated (e.g., [63,75,110]). Reaches were included if it was possible to align the class definitions above with the data or information presented in the report, and a precise location could be determined. In several cases, we contacted study authors to request raw data or obtain their judgments about their study reaches. For example, a previous study in the upper Colorado River basin used USGS gage data to classify streams as perennial, strongly intermittent and weakly intermittent and noted that strongly intermittent reaches likely included some ephemeral reaches [5]. We interviewed the lead author and asked her to identify reaches that she believed matched our definition of an ephemeral reach based on her knowledge of existing hydrologic data and previous reach visits to study reaches.

Local expertise. Streamflow classification was determined through interviews with experts familiar with the stream and specific reach hydrology. To qualify as an expert the person ideally had multiple years of familiarity with the reach and had visited it recently. We provided experts with our definitions of flow duration classes and asked them to classify streams based on direct observations of stream hydrology, e.g., the presence or absence of water, pools or flow and not based on inferences of the timing of streamflow for other streams in the area (e.g., seasonal snowmelt). Experts were also discouraged from considering indirect indicators of flow duration, such as the presence of absence of hydrophytes in the stream channel.

Reclassification of reaches:

When conditions during data collection appeared to be inconsistent with expectations (e.g., surface flow observed at ephemeral reaches despite the lack of recent rainfall), the data underlying the original classification was reviewed; local experts were consulted and re-interviewed whenever possible. If new information revealed that the original classification was incorrect, either a new classification was applied, or the reach was withheld from analysis (with RSC review and approval). A total of 5 reaches were re-evaluated, and all were reclassified from ephemeral to intermittent based on new information.

In three instances, the field crews sampled a reach close to but distinct from the intended location. In one of these instances, consultation with a local expert who knew the sampled location well provided us with information that allowed its subsequent classification. In the other instances, new information could not be found, and the reaches were excluded from analysis.

File S3. Indicator metrics evaluated in the development of the beta SDAM AW

File S3.1: Metric abbreviations, descriptions, and selection criteria

Metrics followed by an asterisk (*) are hydrologic metrics considered direct measures of water presence. (NM) indicates metrics that are scored following the NM protocol [34]. C: Continuous metrics. O: Ordinal metrics. B: Binary metrics. % dom: Percent dominance of most common value. PvIvE-F: F-statistic from an analysis of variance comparing values at perennial, intermittent, and ephemeral reaches. EvALI-t: t-statistic from a comparison of values at ephemeral and at least intermittent reaches. PvNP-t: t-statistic from a comparison of values at perennial and non-perennial reaches. PvIwet-t: t-statistic from a comparison of values at perennial and wet intermittent reaches. PvIdry-t: t-statistic from a comparison of values at ephemeral and dry intermittent reaches. RF-MDA: Variable importance (as mean decrease accuracy) from a random forest model predicting perennial, intermittent, or ephemeral streamflow duration class. Black text indicates metric values that passed screening criteria, while gray text indicates metric values that did not pass screening criteria. To pass, a metric had to pass the % dominance criterion, plus any of the responsiveness criteria.

Indicator	Form	Description	% dom	Responsiveness criteria PvIvE F	EvALI t	PvNP t	PvIwet t	EvIdry t	RF MDA	Pass
**Biological indicators**										
Invertebrate metrics										
bmiabund_score	O	aquatic invertebrate abundance score (NM)	40%	67.72	12.97	9.03	2.69	2.56	0.0067	Yes
TotalAbundance	C	Total aquatic invertebrate abundance	34%	32.63	10.21	5.85	2.64	2.03	0.0094	Yes
Richness	C	Total aquatic invertebrate richness	34%	37.63	10.72	6.14	2.53	2.11	0.0061	Yes
mayfly_abundance	C	Abundance of mayflies	49%	31.82	10.16	5.79	2.63	1.26	0.0049	Yes
perennial_abundance	C	Abundance of perennial indicator taxa	65%	16.05	6.26	4.33	2.55	1.00	0.0004	Yes
perennial_taxa	C	Richness of perennial indicator taxa	65%	19.02	7.49	4.61	2.37	1.00	0.0010	Yes
perennial_live_abundance	C	Abundance of live perennial indicator taxa	66%	15.54	6.05	4.29	2.58	1.00	0.0008	Yes
EPT_abundance	C	Ephemeroptera, Plecoptera, and Trichoptera (EPT) abundance	46%	33.09	9.26	6.13	3.41	1.02	0.0045	Yes
EPT_taxa	C	EPT richness	46%	37.65	10.33	6.65	3.29	1.59	0.0061	Yes
EPT_relabd	C	EPT relative abundance	46%	34.13	10.66	6.40	2.46	1.32	0.0049	Yes
EPT_reltaxa	C	EPT relative richness	46%	34.64	10.96	6.39	2.63	1.49	0.0056	Yes
GOLD_relabd	C	Gastropoda, Oligochaeta, and Diptera (GOLD) relative abundance	43%	9.91	6.06	1.93	0.75	1.48	0.0008	Yes
GOLD_reltaxa	C	GOLD relative richness	43%	11.79	5.78	2.73	0.31	1.41	0.0027	Yes
OCH_relabd	C	Odonata, Coleoptera, and Heteroptera (OCH) relative abundance	56%	2.38	2.03	0.19	1.27	2.27	0.0004	Yes
OCH_reltaxa	C	OCH relative richness	55%	5.10	3.17	0.16	1.40	2.63	0.0004	Yes

GOLDOCH_relabd	C	GOLD + OCH relative abundance	38%	11.32	4.94	1.31	1.65	2.50	0.0017	Yes
GOLDOCH_reltaxa	C	GOLD + OCH relative richness	38%	14.34	5.57	1.94	1.33	2.68	0.0018	Yes
Noninsect_abundance	C	Non-insect abundance	67%	4.01	4.36	1.57	1.08	2.01	0.0001	Yes
Noninsect_taxa	C	Non-insect richness	67%	5.95	5.31	2.00	0.96	2.00	0.0004	Yes
Noninsect_relabund	C	Non-insect relative abundance	67%	3.55	4.05	0.57	0.02	2.06	-0.0002	Yes
Noninsect_reltaxa	C	Non-insect relative richness	67%	4.82	4.93	0.77	0.49	2.19	0.0002	Yes
Vertebrate metrics										
fishabund_score2	O	Fish abundance score (NM) (excluding mosquitofish)	78%	6.16	5.63	2.12	0.73	2.03	0.0001	Yes
snake_score	B	Presence of aquatic snakes	97%	0.75	2.03	0.68	0.54	1.00	-0.0001	
turt_score	B	Presence of aquatic turtles	99%	0.67	1.00	1.00	1.00	0.00	0.0000	
frogvoc_score	B	Presence of frog vocalizations	93%	3.19	1.47	1.93	2.31	0.28	0.0005	Yes
vert_score	B	Presence of aquatic vertebrates	84%	3.29	2.31	0.93	1.77	0.89	0.0002	Yes
vertvoc_score	B	Presence of aquatic vertebrates, including frog vocalizations	81%	3.14	2.04	1.01	1.95	0.50	-0.0003	Yes
vert_sumscore	O	Total number of aquatic vertebrate types detected	84%	3.73	2.47	1.19	1.88	1.06	0.0005	Yes
vertvoc_sumscore	O	Total number of aquatic vertebrate types detected, including frog vocalizations	81%	5.34	2.69	1.86	2.53	0.98	0.0012	Yes
Algal metrics										
algabund_score	O	Algal abundance score (NM)	49%	24.96	8.93	5.06	1.28	1.81	0.0053	Yes
alglive_cover_score	O	Live algal cover on the streambed	51%	24.38	7.89	5.42	1.61	1.53	0.0043	Yes
algdead_cover_score	O	Dead algal cover on the streambed	81%	1.22	1.82	0.06	0.04	1.37	-0.0002	

algdead_noupstream_cover_score	O	Dead algal cover on the streambed, excluding mats deposited from upstream sources	81%	1.53	2.11	0.13	0.04	1.51	0.0006	Yes
alglivedead_cover_score	O	Live or dead algal cover on the streambed	46%	21.86	7.39	5.03	1.61	1.66	0.0017	Yes
Plant metrics										
vegdiff_score	O	Difference in vegetation score (NM)	28%	18.42	5.87	4.80	1.51	1.11	0.0019	Yes
rootedplants_score	O	Upland rooted plants in streambed score (NM)	44%	14.92	4.71	4.15	0.63	0.80	0.0007	Yes
hydrophytes_present_noflag	O	Number of hydrophytic plant species observed (FACW and OBL)	37%	24.29	8.13	5.10	1.85	3.02	0.0042	Yes
moss_cover_score	O	Streamer moss cover in the channel	80%	7.34	4.95	2.81	1.86	1.46	0.0000	Yes
liverwort_cover_score	O	Liverwort cover in the channel	91%	2.04	3.32	0.20	0.77	1.00	0.0000	Yes
PctShading	C	Percent stream shading	19%	10.12	5.41	2.69	1.02	3.32	0.0035	Yes
Other biological metrics										
iofb_score	B	Presence of iron-oxidizing fungi or bacteria (NM)	82%	6.40	5.59	2.30	0.86	1.45	-0.0001	Yes
**Geomorphological indicators**										
sinuosity_score	O	Sinuosity score (NM)	38%	1.70	1.72	0.27	0.51	2.15	-0.0003	Yes
floodplaindim_score	O	Floodplain and channel dimensions score (NM)	27%	0.15	0.40	0.15	0.40	0.79	0.0004	
riffpoolseq_score	O	Riffle-pool sequence score (NM)	28%	11.25	4.10	3.93	2.09	1.93	0.0003	Yes
substratesorting_score	O	Substrate sorting score (NM)	29%	7.34	3.19	3.30	1.23	0.90	0.0001	Yes
seddep_score	O	Sediment deposition score (NM)	75%	0.19	0.62	0.19	0.44	1.25	-0.0002	
BankWidthMean	C	Mean bankfull width	5%	10.16	3.13	1.19	0.43	3.31	0.0029	Yes

valleyslope		C	Valley slope	25%	0.20	0.47	0.14	0.30	0.69	0.0006	
**Hydrologic indicators**											
waterinchannel_score	*	O	Water in channel score (NM)	53%	68.24	10.55	10.27	1.30	2.77	0.0145	Yes
hydric_score		B	Presence of hydric soils (NM)	78%	12.78	6.51	3.82	2.49	1.83	0.0004	Yes
springs_score	*	B	Presence of springs and seeps (NM)	94%	0.06	0.02	0.32	0.06	1.44	-0.0001	
pctsurfaceflow	*	C	Percent surface flow in channel	59%	66.26	11.33	10.72	1.41	0.82	0.0127	Yes
pctsubsurfaceflow	*	B	Percent surface or subsurface flow in channel	60%	59.57	10.53	9.22	0.55	1.53	0.0105	Yes
numberwoodyjams		B	Number of woody jams in reach	84%	0.79	1.28	0.91	0.81	1.15	0.0001	
SoilMoist_MaxScore	*	O	Maximum soil moisture	69%	58.23	8.86	8.05	0.00	2.77	0.0133	Yes
**Geospatial indicators**											
Point-based metrics											
EcoII		C	Level 2 ecoregion	26%	0.49	1.09	0.69	0.04	0.94	0.0003	
EcoIII		C	Level 3 ecoregion	16%	5.18	2.80	2.26	1.74	2.65	0.0001	Yes
tmean		C	30-year normal mean annual temperature at the reach	1%	8.57	3.96	2.29	0.78	3.10	0.0032	Yes
tmax		C	30-year normal maximum annual temperature at the reach	1%	8.72	3.98	2.28	0.80	3.09	0.0028	Yes
tmin		C	30-year normal minimum annual temperature at the reach	1%	7.76	3.85	2.22	0.73	2.90	0.0034	Yes
ppt		C	30-year normal total annual precipitation at the reach	1%	4.47	4.50	0.68	0.24	1.99	0.0007	Yes
ppt.m01		C	30-year normal total January precipitation at the reach	1%	4.61	4.62	0.19	0.88	1.74	0.0003	Yes

ppt.m02	C	30-year normal total February precipitation at the reach	1%	4.82	4.54	0.19	0.98	1.71	0.0002	Yes
ppt.m03	C	30-year normal total March precipitation at the reach	1%	5.04	4.79	0.41	0.67	1.86	0.0004	Yes
ppt.m04	C	30-year normal total April precipitation at the reach	2%	4.79	4.49	0.85	0.20	2.03	0.0010	Yes
ppt.m05	C	30-year normal total May precipitation at the reach	1%	2.64	2.76	1.13	0.45	1.73	0.0010	Yes
ppt.m06	C	30-year normal total June precipitation at the reach	1%	0.72	1.24	0.58	0.40	1.16	0.0014	Yes
ppt.m07	C	30-year normal total July precipitation at the reach	2%	0.91	0.51	0.93	1.41	0.29	-0.0001	
ppt.m08	C	30-year normal total August precipitation at the reach	1%	1.32	1.25	0.49	1.26	0.20	0.0006	
ppt.m09	C	30-year normal total September precipitation at the reach	1%	0.57	0.08	0.91	1.37	0.80	-0.0003	
ppt.m10	C	30-year normal total October precipitation at the reach	1%	3.23	3.53	1.17	0.70	1.90	0.0018	Yes
ppt.m11	C	30-year normal total November precipitation at the reach	1%	4.02	4.52	0.36	0.33	1.83	0.0022	Yes
ppt.m12	C	30-year normal total December precipitation at the reach	1%	3.96	4.20	0.13	0.88	1.68	0.0009	Yes
StreamCat metrics										
ElevCat	C	Mean elevation in the catchment	2%	3.68	2.57	2.06	0.91	1.59	0.0005	Yes
ElevWs	C	Mean elevation in the watershed	2%	6.62	3.15	3.14	1.83	1.68	0.0003	Yes
AgKffactCat	C	Soil erodibility (k-factor) in agricultural lands in the catchment	69%	0.25	0.67	0.54	0.77	0.66	-0.0001	

KffactCat	C	Soil erodibility (k-factor) in the catchment	2%	0.50	1.00	0.27	0.94	0.25	-0.0002	
AgKffactWs	C	Soil erodibility (k-factor) in agricultural lands in the watershed	45%	1.21	2.63	0.81	0.64	0.99	-0.0006	Yes
KffactWs	C	Soil erodibility (k-factor) in the watershed	2%	0.34	0.70	0.67	0.12	1.10	0.0001	
Al2O3Cat	C	Al2O3 content in the catchment geology	2%	0.43	0.81	0.82	0.01	0.13	-0.0005	
CaOCat	C	CaO content in the catchment geology	2%	2.04	2.37	0.50	0.06	1.37	-0.0011	Yes
Fe2O3Cat	C	Fe2O3 content in the catchment geology	2%	0.26	0.69	0.01	0.15	0.86	-0.0003	
K2OCat	C	K2O content in the catchment geology	2%	0.12	0.52	0.22	0.63	0.53	-0.0005	
MgOCat	C	MgO content in the catchment geology	2%	3.22	3.04	1.55	1.32	1.75	0.0008	Yes
Na2OCat	C	Na2O content in the catchment geology	2%	1.27	1.70	1.03	0.05	0.86	0.0006	
P2O5Cat	C	P2O5 content in the catchment geology	2%	0.50	1.09	0.10	0.51	1.68	-0.0003	
SCat	C	S content in the catchment geology	2%	0.34	0.53	0.85	0.64	0.30	0.0006	
SiO2Cat	C	SiO2 content in the catchment geology	2%	0.79	1.37	0.30	0.27	1.01	-0.0003	
Al2O3Ws	C	Al2O3 content in the watershed geology	2%	1.78	1.78	1.02	0.89	0.29	-0.0008	
CaOWs	C	CaO content in the watershed geology	2%	0.69	0.80	0.31	1.63	0.43	-0.0002	
Fe2O3Ws	C	Fe2O3 content in the watershed geology	2%	1.25	1.30	1.38	0.22	0.61	0.0007	
K2OWs	C	K2O content in the watershed geology	2%	0.14	0.47	0.02	1.12	0.17	-0.0002	

MgOWs	C	MgO content in the watershed geology	2%	0.50	0.65	0.28	2.70	0.29	0.0007	Yes
Na2OWs	C	Na2O content in the watershed geology	2%	1.03	1.38	0.95	0.41	0.32	-0.0002	
P2O5Ws	C	P2O5 content in the watershed geology	2%	0.89	1.38	0.55	0.58	1.25	0.0002	
SWs	C	S content in the watershed geology	2%	0.74	1.07	1.44	0.69	1.47	-0.0004	
SiO2Ws	C	SiO2 content in the watershed geology	2%	0.91	0.61	0.76	2.33	0.38	-0.0001	Yes
NCat	C	N content in the catchment geology	2%	1.23	1.60	0.92	0.47	1.03	0.0003	
NWs	C	N content in the watershed geology	2%	1.19	1.27	0.73	0.60	0.68	-0.0003	
HydrlCondCat	C	Mean hydraulic conductivity in the catchment	2%	2.18	1.65	0.57	0.31	1.16	-0.0007	Yes
HydrlCondWs	C	Mean hydraulic conductivity in the watershed	2%	1.11	1.09	0.32	0.62	1.33	-0.0002	
CompStrgthCat	C	Mean compressive strength in the catchment	2%	5.80	3.62	1.55	0.32	1.91	-0.0001	Yes
CompStrgthWs	C	Mean compressive strength in the watershed	2%	2.62	2.28	0.88	0.12	0.84	-0.0010	Yes
Precip8110Cat	C	30-year normal total annual precipitation in the catchment	2%	4.34	4.44	0.63	0.30	1.88	0.0004	Yes
Tmax8110Cat	C	30-year normal max annual temperature in the catchment	2%	9.79	4.11	2.67	0.92	2.99	0.0004	Yes
Tmean8110Cat	C	30-year normal mean annual temperature in the catchment	2%	9.68	4.11	2.69	0.93	3.07	0.0014	Yes
Tmin8110Cat	C	30-year normal min annual temperature in the catchment	2%	8.92	4.03	2.63	0.91	2.97	0.0018	Yes
Precip8110Ws	C	30-year normal total annual precipitation in the watershed	2%	5.57	5.39	1.67	0.60	1.81	0.0000	Yes

Tmax8110Ws	C	30-year normal max annual temperature in the watershed	2%	12.44	4.67	3.57	1.80	3.41	0.0005	Yes
Tmean8110Ws	C	30-year normal mean annual temperature in the watershed	2%	11.40	4.52	3.36	1.59	3.32	0.0024	Yes
Tmin8110Ws	C	30-year normal min annual temperature in the watershed	2%	9.93	4.28	3.07	1.35	3.06	0.0005	Yes
ClayCat	C	Mean % clay content of soils in the catchment	2%	0.64	0.64	0.60	0.34	2.23	- 0.0006	Yes
SandCat	C	Mean % sand content of soils in the catchment	2%	0.16	0.41	0.11	0.59	1.63	- 0.0004	
ClayWs	C	Mean % clay content of soils in the watershed	2%	0.97	0.84	1.34	0.77	1.32	- 0.0001	
SandWs	C	Mean % sand content of soils in the watershed	2%	0.83	0.81	1.20	0.10	1.56	0.0001	
OmCat	C	Mean % oragnic matter content of soils in the catchment	2%	2.54	3.04	0.20	0.21	1.56	- 0.0007	Yes
PermCat	C	Mean permeability of soils in the catchment	2%	0.84	0.75	0.48	0.10	1.74	- 0.0002	
RckDepCat	C	Mean depth to bedrock in the catchment	13%	0.55	1.02	0.16	0.46	0.21	0.0005	
WtDepCat	C	Mean seasonal water table depth in the catchment	55%	0.99	1.73	0.83	0.96	1.30	0.0005	
OmWs	C	Mean % oragnic matter content of soils in the watershed	2%	1.57	1.98	0.08	0.85	1.20	- 0.0005	
PermWs	C	Mean permeability of soils in the watershed	2%	1.50	1.02	1.50	0.67	0.95	- 0.0001	
RckDepWs	C	Mean depth to bedrock in the watershed	2%	2.12	0.46	2.09	1.29	0.41	0.0009	Yes
WtDepWs	C	Mean seasonal water table depth in the watershed	41%	0.49	0.86	0.73	0.15	0.57	0.0000	

PctCarbResidCat	C	Percent carbonate residual material in catchment geology	92%	0.51	0.60	0.74	0.77	0.32	0.0000	
PctNonCarbResidCat	C	Percent non-carbonate residual material in catchment geology	48%	0.23	0.35	0.70	0.16	0.01	-0.0001	
PctAlkIntruVolCat	C	Percent alkaline intrusive volcanic material in catchment geology	98%	3.12	1.44	1.42	1.00	1.00	0.0000	
PctSilicicCat	C	Percent silicic material in catchment geology	52%	4.62	3.94	1.29	0.14	1.71	0.0007	Yes
PctEolCrsCat	C	Percent coarse eolian sediment material in catchment geology	99%	1.51	1.00	1.00	0.00	1.00	0.0000	
PctEolFineCat	C	Percent fine eolian sediment material in catchment geology	100%	0.00	0.00	0.00	0.00	0.00	0.0000	
PctAlluvCoastCat	C	Percent alluvium and fine-textured coastal zone sediment material in catchment geology	49%	2.87	2.06	0.42	0.79	1.56	-0.0002	Yes
PctCarbResidWs	C	Percent carbonate residual material in watershed geology	65%	0.13	0.47	0.15	0.36	0.41	0.0001	
PctNonCarbResidWs	C	Percent non-carbonate residual material in watershed geology	25%	0.93	0.95	1.35	0.02	0.09	0.0002	
PctAlkIntruVolWs	C	Percent alkaline intrusive volcanic material in watershed geology	98%	3.12	1.44	1.42	1.00	1.00	0.0000	
PctSilicicWs	C	Percent silicic material in watershed geology	30%	3.29	2.85	1.00	0.30	1.06	0.0001	Yes
PctExtruVolWs	C	Percent extrusive volcanic material in watershed geology	95%	0.67	1.53	0.75	0.68	1.14	0.0001	

PctEolCrsWs	C	Percent coarse eolian sediment material in watershed geology	96%	0.67	0.36	1.26	0.54	0.83	0.0000	
PctEolFineWs	C	Percent fine eolian sediment material in watershed geology	98%	0.33	1.41	0.38	1.00	1.00	0.0000	
PctAlluvCoastWs	C	Percent alluvium and fine-textured coastal zone sediment material in watershed geology	41%	1.57	1.53	0.11	0.64	1.72	-0.0003	
WetIndexCat	C	Mean composite topographic index (wetness index) in the catchment	2%	3.01	1.85	2.25	0.98	0.57	0.0001	Yes
WetIndexWs	C	Mean composite topographic index (wetness index) in the watershed	2%	0.95	1.48	0.85	0.29	0.68	0.0005	
urb	C	Total percent urban landcover in the catchment	28%	3.62	2.11	1.26	0.95	2.63	0.0001	Yes
urbRip	C	Total percent urban landcover in the catchment within 100 m of the stream flow-line	36%	1.05	1.39	0.37	0.48	2.56	0.0003	Yes
forr	C	Total percent forested landcover in the catchment	36%	5.07	3.76	2.27	1.50	2.03	0.0006	Yes
forrRip	C	Total percent forested landcover in the catchment within 100 m of the stream flow-line	44%	4.79	4.13	1.99	0.92	1.95	0.0002	Yes
ag	C	Total percent agricultural landcover in the catchment	70%	0.17	0.66	0.39	0.56	0.67	-0.0003	
agRip	C	Total percent agricultural landcover in the catchment within 100 m of the stream flow-line	80%	0.79	1.35	0.97	1.55	1.01	-0.0001	

wetlands	C	Total percent wetlands landcover in the catchment	41%	4.39	3.57	2.29	1.41	0.94	0.0004	Yes
wetlandsRip	C	Total percent wetlands landcover in the catchment within 100 m of the stream flow-line	45%	9.40	3.98	3.27	2.54	0.59	0.0020	Yes
urbWs	C	Total percent urban landcover in the watershed	17%	0.76	1.06	0.23	0.66	2.40	-0.0003	Yes
urbRipWs	C	Total percent urban landcover in the watershed within 100 m of the stream flow-line	21%	0.57	0.54	0.57	0.10	2.25	0.0002	Yes
forrWs	C	Total percent forested landcover in the watershed	2%	12.41	5.17	3.85	2.60	3.09	0.0011	Yes
forrWsRip	C	Total percent forested landcover in the watershed within 100 m of the stream flow-line	3%	13.56	5.56	3.96	2.44	3.04	0.0017	Yes
agWs	C	Total percent agricultural landcover in the watershed	51%	0.91	2.34	0.50	0.24	0.92	-0.0002	Yes
agRipWs	C	Total percent agricultural landcover in the watershed within 100 m of the stream flow-line	55%	1.10	2.56	0.70	0.64	1.00	-0.0004	Yes
wetlandsWs	C	Total percent wetlands landcover in the watershed	20%	3.76	3.95	1.85	0.43	0.03	0.0004	Yes
wetlandsRipWS	C	Total percent wetlands landcover in the watershed within 100 m of the stream flow-line	23%	7.48	4.98	2.67	0.75	0.97	-0.0006	Yes

File S3.2 Spearman rank correlations between indicators and ordination axes shown in **Error! Reference source not found.**

Part 1: Biological, geomorphological, and hydrologic indicators

Indicator	Rho1	Rho2	Indicator	Rho1	Rho2
Biological indicators					
Invertebrate metrics			Geomorphological indicators		
bmiabund_score	-0.88	-0.08	sinuosity_score	0.01	0.07
TotalAbundance	-0.90	-0.08	floodplaindim_score	-0.12	0.02
Richness	-0.90	-0.10	riffpoolseq_score	-0.59	0.07
mayfly_abundance	-0.83	-0.12	substratesorting_score	-0.63	-0.05
perennial_abundance	-0.72	-0.15	seddep_score	-0.25	-0.20
perennial_taxa	-0.71	-0.14	BankWidthMean	0.14	-0.01
perennial_live_abundance	-0.72	-0.15	valleyslope	-0.24	0.04
EPT_abundance	-0.84	-0.11			
EPT_taxa	-0.83	-0.14	Hydrologic indicators		
EPT_relabd	-0.76	-0.16	waterinchannel_score	-0.85	-0.29
EPT_reltaxa	-0.76	-0.15	hydric_score	-0.50	-0.40
GOLD_relabd	-0.70	0.12	springs_score	-0.18	0.17
GOLD_reltaxa	-0.70	0.10	pctsurfaceflow	-0.82	-0.19
OCH_relabd	-0.64	-0.11	pctsubsurfaceflow	-0.81	-0.26
OCH_reltaxa	-0.61	-0.11	numberwoodyjams	0.01	0.05
GOLDOCH_relabd	-0.67	-0.02	SoilMoist_MaxScore	-0.84	-0.15
GOLDOCH_reltaxa	-0.67	-0.05			
Noninsect_abundance	-0.60	0.08	Geospatial indicators		
Noninsect_taxa	-0.60	0.08	Point-based metrics		
Noninsect_relabund	-0.55	0.13	EcoII	-0.29	0.12
Noninsect_reltaxa	-0.55	0.13	EcoIII	-0.05	0.18
Vertebrate metrics			tmean	0.01	0.16
fishabund_score2	-0.37	-0.25	tmax	0.02	0.16
snake_score	-0.23	0.23	tmin	0.03	0.14
turt_score	-0.15	0.12	ppt	-0.39	0.12

frogvoc_score	-0.31	0.36	ppt.m01	-0.34	0.05
vert_score	-0.51	0.63	ppt.m02	-0.31	0.06
vertvoc_score	-0.53	0.65	ppt.m03	-0.31	0.08
vert_sumscore	-0.51	0.63	ppt.m04	-0.17	0.07
vertvoc_sumscore	-0.53	0.65	ppt.m05	0.02	-0.01
Algal metrics			ppt.m06	0.05	-0.05
algabund_score	-0.77	0.16	ppt.m07	0.00	0.04
alglive_cover_score	-0.81	0.15	ppt.m08	0.02	0.02
algdead_cover_score	-0.36	0.21	ppt.m09	0.05	0.02
algdead_noupstream_cover_score	-0.39	0.20	ppt.m10	0.03	-0.02
alglivedead_cover_score	-0.75	0.23	ppt.m11	-0.30	0.04
Plant metrics			ppt.m12	-0.36	0.04
vegdiff_score	-0.62	-0.29			
rootedplants_score	-0.59	-0.27			
hydrophytes_present_noflag	-0.67	-0.20			
moss_cover_score	-0.29	0.03			
liverwort_cover_score	-0.14	-0.12			
PctShading	-0.35	-0.16			
Other biological metrics					
iofb_score	-0.44	-0.22			

File S3.2, Part 2: Geospatial metrics from the StreamCat dataset [40].

StreamCat metrics	Rho1	Rho2	StreamCat metrics	Rho1	Rho2
ElevCat	-0.01	-0.11	OmCat	-0.16	0.05
ElevWs	-0.02	-0.24	PermCat	-0.08	-0.22
AgKffactCat	0.17	-0.08	RckDepCat	0.18	-0.13
KffactCat	0.20	0.02	WtDepCat	0.06	-0.08
AgKffactWs	0.02	-0.02	OmWs	-0.11	0.00
KffactWs	0.10	0.06	PermWs	-0.11	-0.13
Al2O3Cat	-0.07	0.07	RckDepWs	-0.02	-0.08
CaOCat	-0.25	0.06	WtDepWs	0.15	0.00
Fe2O3Cat	0.10	-0.13	PctCarbResidCat	-0.08	0.20
K2OCat	0.10	0.04	PctNonCarbResidCat	-0.04	-0.06
MgOCat	-0.32	0.10	PctSilicicCat	-0.26	0.10
Na2OCat	-0.08	0.02	PctEolCrsCat	0.14	-0.05
P2O5Cat	0.06	-0.07	PctAlluvCoastCat	0.23	-0.14
SCat	0.04	-0.10	PctCarbResidWs	0.06	0.17
SiO2Cat	0.09	0.11	PctNonCarbResidWs	0.22	-0.03
Al2O3Ws	-0.29	-0.06	PctSilicicWs	-0.36	-0.01
CaOWs	-0.01	0.13	PctExtruVolWs	0.06	-0.06
Fe2O3Ws	-0.16	-0.14	PctEolCrsWs	0.03	-0.16
K2OWs	-0.14	-0.08	PctEolFineWs	0.01	-0.20
MgOWs	-0.09	0.14	PctAlluvCoastWs	0.12	-0.14
Na2OWs	-0.27	-0.06	WetIndexCat	0.30	-0.10
P2O5Ws	-0.08	0.04	WetIndexWs	0.26	-0.03
SWs	0.32	0.08	urb	0.08	-0.18
SiO2Ws	-0.05	-0.02	urbRip	0.05	-0.13
NCat	0.21	-0.18	forr	-0.18	-0.08
NWs	0.25	-0.12	forrRip	-0.23	0.02
HydrlCondCat	0.20	-0.15	ag	0.17	-0.05
HydrlCondWs	0.08	-0.16	agRip	0.05	-0.06
CompStrgthCat	-0.30	0.12	wetlands	-0.33	-0.02
CompStrgthWs	-0.32	0.05	wetlandsRip	-0.35	0.05
Precip8110Cat	-0.39	0.12	urbWs	-0.08	-0.13
Tmax8110Cat	0.07	0.13	urbRipWs	-0.11	-0.18
Tmean8110Cat	0.05	0.12	forrWs	-0.23	-0.14
Tmin8110Cat	0.06	0.12	forrWsRip	-0.27	-0.15
Precip8110Ws	-0.42	-0.19	agWs	-0.04	-0.10
Tmax8110Ws	0.06	0.25	agRipWs	-0.10	-0.08
Tmean8110Ws	0.04	0.23	wetlandsWs	-0.29	-0.03
Tmin8110Ws	0.05	0.18	wetlandsRipWS	-0.33	-0.02
ClayCat	0.05	0.13			
SandCat	-0.09	-0.12			
ClayWs	-0.05	0.07			
SandWs	-0.01	-0.11			

File S4. Performance evaluation measures for all models across each sub-region.

File S4.1. Plot of performance statistics for all models by sub-region.

The outlined symbols represent final selected model, and SDAM AW represents the final, simplified version that includes single indicators. EnotP: Proportion of ephemeral reaches correctly not classified as perennial. EvALI: Proportion of reaches correctly classified as ephemeral or at least intermittent. Proportion of dry reaches correctly classified as ephemeral or intermittent. PnotE: Proportion of perennial reaches correctly not classified as ephemeral. PvIvE: Proportion of reaches correctly classified perennial, intermittent, or ephemeral. PvIwet: Proportion of flowing reaches correctly classified as perennial or intermittent. PvNP: Proportion of reaches correctly classified as perennial or non-perennial. GIS: Models that include geospatial data. H2O: Models that include direct measures of water presence. Other: Models that either exclude geospatial metrics and direct measures of water presence, or results from the PNW and NM SDAMs.

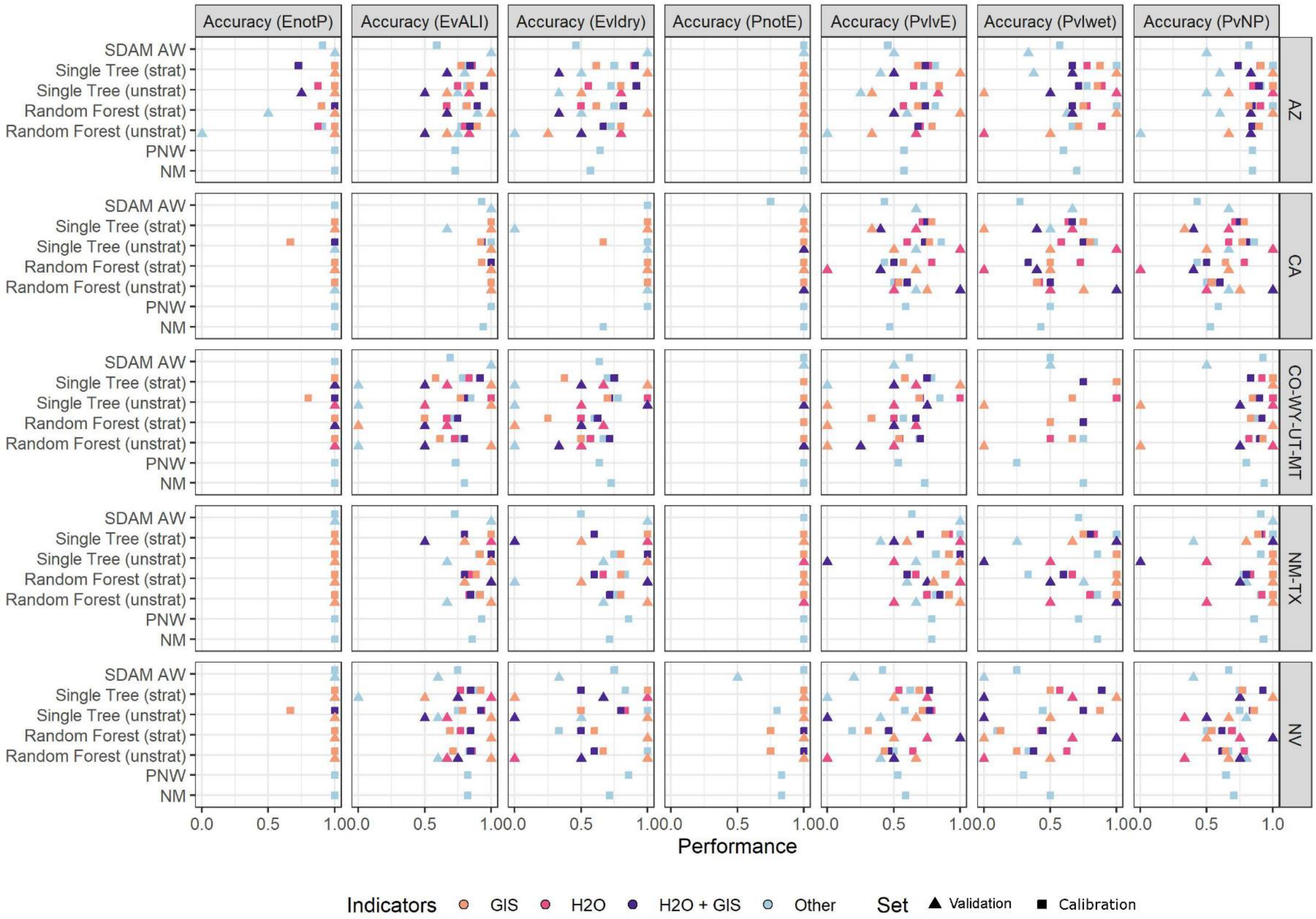
Accuracy (EnotP)
Accuracy (EvALI)
Accuracy (Evldry)
Accuracy (PnotE)
Accuracy (PvlvE)
Accuracy (Pvlwet)
Accuracy (PvNP)
SDAM AW
Single Tree (strat)
Single Tree (unstrat)
Random Forest (strat)
Random Forest (unstrat)
PNW
NM
AZ
CA
CO-WY-UT-MT
NM-TX
NV
0.0
0.5
1.0
Performance
Indicators
GIS
H2O
H2O + GIS
Other
Set
Validation
Calibration

File S4.2 Table of performance statistics for all models by sub-region

SDAM AW: the final Streamflow Duration Assessment Method for the AW, including all modifications and use of single indicators. NM: the New Mexico method [34]. PNW: the Pacific Northwest method [27]. Base: Biological metrics, geomorphological metrics, and hydrologic metrics that did not directly measure the presence of water. GIS: Geospatial metrics. H2O: Hydrological metrics that directly measure the presence of surface water. Model type is the approach used to develop the final method. ST: single decision tree. RF: random forest. Accuracy measures are proportion of correct classifications for a variety of comparisons. EnotP: Proportion of ephemeral reaches correctly not classified as perennial. EvIdry: Proportion of dry reaches correctly classified as ephemeral or intermittent. EvALI: Proportion of reaches correctly classified as ephemeral or at least intermittent reaches. PnotE: Proportion of perennial reaches correctly not classified as ephemeral. PvIvE: Proportion of reaches correctly classified as perennial, intermittent, or ephemeral. PvIwet: Proportion of flowing reaches correctly classified as perennial or intermittent. PvNP: Proportion of reaches correctly classified as perennial or non-perennial. Repeatability: Proportion of revisited reaches with the same classification for each visit. n: Number of reaches evaluated in the comparison. % cor: Percent correct. Asterisk indicates the model that was selected for refinement to create the final model. Due to the small sizes of data sets, calibration and validation results are combined.

				AZ		CA		CO-WY-UT-MT		NM-TX		NV	
Final methods			Accuracy Measure	n	% cor	n	% cor	n	% cor	n	% cor	n	% cor
SDAM AW			EnotP	12	0.92	3	1.00	6	1.00	5	1.00	4	1.00
SDAM AW			EvIdry	14	0.57	3	1.00	11	0.64	7	0.86	7	0.71
SDAM AW			EvALI	26	0.73	17	0.94	15	0.73	14	0.86	17	0.88
SDAM AW			PnotE	6	1.00	5	0.80	3	1.00	5	1.00	6	1.00
SDAM AW			PvIvE	26	0.54	17	0.53	15	0.60	14	0.79	17	0.47
SDAM AW			PvIwet	10	0.60	14	0.43	4	0.50	7	0.71	10	0.30
SDAM AW			PvNP	26	0.77	17	0.53	15	0.87	14	0.93	17	0.59
NM			EnotP	12	1.00	3	1.00	6	1.00	5	1.00	4	1.00
NM			EvIdry	14	0.57	3	0.67	11	0.73	7	0.71	7	0.71
NM			EvALI	26	0.73	17	0.94	15	0.80	14	0.86	17	0.82
NM			PnotE	6	1.00	5	1.00	3	1.00	5	1.00	6	0.83
NM			PvIvE	26	0.58	17	0.47	15	0.73	14	0.79	17	0.59
NM			PvIwet	10	0.70	14	0.43	4	0.75	7	0.86	10	0.50
NM			PvNP	26	0.85	17	0.53	15	0.93	14	0.93	17	0.71
PNW			EnotP	12	1.00	3	1.00	6	1.00	5	1.00	4	1.00
PNW			EvIdry	14	0.64	3	1.00	11	0.64	7	0.86	7	0.86
PNW			EvALI	26	0.73	17	1.00	15	0.73	14	0.93	17	0.82
PNW			PnotE	6	1.00	5	1.00	3	1.00	5	1.00	6	0.83
PNW			PvIvE	26	0.58	17	0.59	15	0.53	14	0.79	17	0.53
PNW			PvIwet	10	0.60	14	0.50	4	0.25	7	0.71	10	0.30
PNW			PvNP	26	0.85	17	0.59	15	0.80	14	0.86	17	0.65
Calibrated models													
Indicators	Stratified	Model Type	Accuracy Measure										
Base	No	RF	EnotP	12	0.83	3	1.00	6	1.00	5	1.00	4	1.00
Base	No	RF	EvIdry	14	0.57	3	1.00	11	0.55	7	0.71	7	0.71
Base	No	RF	EvALI	26	0.77	17	1.00	15	0.67	14	0.86	17	0.76
Base	No	RF	PnotE	6	1.00	5	1.00	3	1.00	5	1.00	6	1.00

Base	No	RF	PvIvE	26	0.58	17	0.53	15	0.60	14	0.79	17	0.47
Base	No	RF	PvIwet	10	0.60	14	0.43	4	0.75	7	0.86	10	0.30
Base	No	RF	PvNP	26	0.73	17	0.53	15	0.93	14	0.93	17	0.71
Base	No	ST	EnotP	12	1.00	3	1.00	6	1.00	5	1.00	4	1.00
Base	No	ST	EvIdry	14	0.64	3	1.00	11	0.64	7	0.71	7	0.71
Base	No	ST	EvALI	26	0.81	17	1.00	15	0.73	14	0.86	17	0.71
Base	No	ST	PnotE	6	1.00	5	1.00	3	1.00	5	1.00	6	0.83
Base	No	ST	PvIvE	26	0.65	17	0.82	15	0.73	14	0.79	17	0.53
Base	No	ST	PvIwet	10	0.70	14	0.79	4	1.00	7	0.86	10	0.40
Base	No	ST	PvNP	26	0.85	17	0.82	15	1.00	14	0.93	17	0.76
Base	Yes	RF	EnotP	12	0.92	3	1.00	6	1.00	5	1.00	4	1.00
Base	Yes	RF	EvIdry	14	0.71	3	1.00	11	0.55	7	0.71	7	0.43
Base	Yes	RF	EvALI	26	0.85	17	1.00	15	0.67	14	0.86	17	0.71
Base	Yes	RF	PnotE	6	1.00	5	1.00	3	1.00	5	1.00	6	1.00
Base	Yes	RF	PvIvE	26	0.73	17	0.47	15	0.53	14	0.64	17	0.24
Base	Yes	RF	PvIwet	10	0.70	14	0.36	4	0.50	7	0.57	10	0.10
Base	Yes	RF	PvNP	26	0.85	17	0.47	15	0.87	14	0.79	17	0.53
Base	Yes	ST	EnotP	12	1.00	3	1.00	6	1.00	5	1.00	4	1.00
Base	Yes	ST	EvIdry	14	0.71	3	0.67	11	0.64	7	1.00	7	0.71
Base	Yes	ST	EvALI	26	0.81	17	0.94	15	0.73	14	1.00	17	0.82
Base	Yes	ST	PnotE	6	1.00	5	1.00	3	1.00	5	1.00	6	1.00
Base	Yes	ST	PvIvE	26	0.65	17	0.71	15	0.73	14	0.79	17	0.59
Base	Yes	ST	PvIwet	10	0.50	14	0.71	4	1.00	7	0.57	10	0.50
Base	Yes	ST	PvNP	26	0.85	17	0.76	15	1.00	14	0.79	17	0.76
GIS	No	RF	EnotP	12	1.00	3	1.00	5	1.00	5	1.00	4	1.00
GIS	No	RF	EvIdry	14	0.64	3	1.00	10	0.50	7	0.86	7	0.71
GIS	No	RF	EvALI	25	0.84	17	1.00	14	0.64	14	0.93	17	0.76
GIS	No	RF	PnotE	5	1.00	5	1.00	3	1.00	5	1.00	6	0.83
GIS	No	RF	PvIvE	25	0.68	17	0.59	14	0.50	14	0.93	17	0.47
GIS	No	RF	PvIwet	9	0.67	14	0.50	4	0.50	7	1.00	10	0.30
GIS	No	RF	PvNP	25	0.84	17	0.59	14	0.86	14	1.00	17	0.65
GIS	No	ST	EnotP	12	1.00	3	0.67	5	0.80	5	1.00	4	0.75

GIS	No	ST	EvIdry	14	0.71	3	0.67	10	0.70	7	0.86	7	0.57
GIS	No	ST	EvALI	25	0.80	17	0.94	14	0.79	14	0.93	17	0.82
GIS	No	ST	PnotE	5	1.00	5	1.00	3	1.00	5	1.00	6	1.00
GIS	No	ST	PvIvE	25	0.72	17	0.71	14	0.64	14	0.93	17	0.71
GIS	No	ST	PvIwet	9	0.67	14	0.71	4	0.50	7	1.00	10	0.80
GIS	No	ST	PvNP	25	0.92	17	0.71	14	0.79	14	1.00	17	0.82
GIS	Yes	RF	EnotP	12	0.92	3	1.00	5	1.00	5	1.00	4	1.00
GIS	Yes	RF	EvIdry	14	0.64	3	1.00	10	0.20	7	0.71	7	0.71
GIS	Yes	RF	EvALI	25	0.84	17	0.94	14	0.43	14	0.86	17	0.76
GIS	Yes	RF	PnotE	5	1.00	5	1.00	3	1.00	5	1.00	6	0.83
GIS	Yes	RF	PvIvE	25	0.72	17	0.59	14	0.29	14	0.86	17	0.35
GIS	Yes	RF	PvIwet	9	0.78	14	0.50	4	0.50	7	1.00	10	0.10
GIS	Yes	RF	PvNP	25	0.84	17	0.65	14	0.86	14	1.00	17	0.53
GIS	Yes	ST	EnotP	12	1.00	3	1.00	5	1.00	5	1.00	4	1.00
GIS	Yes	ST	EvIdry	14	0.64	3	1.00	10	0.50	7	0.86	7	0.71
GIS	Yes	ST	EvALI	25	0.80	17	1.00	14	0.64	14	0.93	17	0.82
GIS	Yes	ST	PnotE	5	1.00	5	1.00	3	1.00	5	1.00	6	1.00
GIS	Yes	ST	PvIvE	25	0.72	17	0.71	14	0.64	14	0.79	17	0.65
GIS	Yes	ST	PvIwet	9	0.89	14	0.64	4	1.00	7	0.71	10	0.60
GIS	Yes	ST	PvNP	25	0.92	17	0.71	14	1.00	14	0.86	17	0.82
H2O	No	RF	EnotP	12	0.92	3	1.00	6	1.00	5	1.00	4	1.00
H2O	No	RF	EvIdry	14	0.71	3	1.00	11	0.55	7	0.71	7	0.57
H2O	No	RF	EvALI	26	0.81	17	1.00	15	0.67	14	0.86	17	0.82
H2O	No	RF	PnotE	6	1.00	5	1.00	3	1.00	5	1.00	6	1.00
H2O	No	RF	PvIvE	26	0.69	17	0.53	15	0.53	14	0.71	17	0.53
H2O	No	RF	PvIwet	10	0.80	14	0.43	4	0.50	7	0.71	10	0.50
H2O	No	RF	PvNP	26	0.85	17	0.53	15	0.87	14	0.86	17	0.71
H2O	No	ST	EnotP	12	0.92	3	1.00	6	1.00	5	1.00	4	1.00
H2O	No	ST	EvIdry	14	0.64	3	0.67	11	0.82	7	1.00	7	0.71
H2O	No	ST	EvALI	26	0.77	17	0.94	15	0.87	14	1.00	17	0.88
H2O	No	ST	PnotE	6	1.00	5	1.00	3	1.00	5	1.00	6	1.00
H2O	No	ST	PvIvE	26	0.69	17	0.65	15	0.87	14	0.93	17	0.65

H2O	No	ST	PvIwet	10	0.90	14	0.64	4	1.00	7	0.86	10	0.60
H2O	No	ST	PvNP	26	0.88	17	0.71	15	1.00	14	0.93	17	0.76
H2O	Yes	RF	EnotP	12	1.00	3	1.00	6	1.00	5	1.00	4	1.00
H2O	Yes	RF	EvIdry	14	0.64	3	1.00	11	0.55	7	0.71	7	0.57
H2O	Yes	RF	EvALI	26	0.73	17	1.00	15	0.67	14	0.86	17	0.82
H2O	Yes	RF	PnotE	6	1.00	5	1.00	3	1.00	5	1.00	6	1.00
H2O	Yes	RF	PvIvE	26	0.65	17	0.65	15	0.53	14	0.71	17	0.53
H2O	Yes	RF	PvIwet	10	0.80	14	0.57	4	0.50	7	0.71	10	0.50
H2O	Yes	RF	PvNP	26	0.92	17	0.65	15	0.87	14	0.86	17	0.71
H2O	Yes	ST	EnotP	12	1.00	3	1.00	6	1.00	5	1.00	4	1.00
H2O	Yes	ST	EvIdry	14	0.93	3	1.00	11	0.73	7	1.00	7	0.57
H2O	Yes	ST	EvALI	26	0.88	17	1.00	15	0.80	14	1.00	17	0.82
H2O	Yes	ST	PnotE	6	1.00	5	1.00	3	1.00	5	1.00	6	1.00
H2O	Yes	ST	PvIvE	26	0.81	17	0.71	15	0.73	14	0.93	17	0.59
H2O	Yes	ST	PvIwet	10	0.80	14	0.64	4	0.75	7	0.86	10	0.60
H2O	Yes	ST	PvNP	26	0.92	17	0.71	15	0.93	14	0.93	17	0.76
H2O + GIS	No	RF	EnotP	12	1.00	3	1.00	5	1.00	5	1.00	4	1.00
H2O + GIS	No	RF	EvIdry	14	0.64	3	1.00	10	0.60	7	0.71	7	0.57
H2O + GIS	No	RF	EvALI	25	0.76	17	1.00	14	0.71	14	0.86	17	0.82
H2O + GIS	No	RF	PnotE	5	1.00	5	1.00	3	1.00	5	1.00	6	1.00
H2O + GIS	No	RF	PvIvE	25	0.60	17	0.65	14	0.57	14	0.86	17	0.47
H2O + GIS	No	RF	PvIwet	9	0.67	14	0.57	4	0.50	7	1.00	10	0.40
H2O + GIS	No	RF	PvNP	25	0.84	17	0.65	14	0.86	14	1.00	17	0.65
H2O + GIS	No	ST	EnotP	12	0.92	3	1.00	5	1.00	5	1.00	4	1.00
H2O + GIS	No	ST	EvIdry	14	0.86	3	0.67	10	0.80	7	1.00	7	0.57
H2O + GIS	No	ST	EvALI	25	0.84	17	0.94	14	0.86	14	1.00	17	0.82
H2O + GIS	No	ST	PnotE	5	1.00	5	1.00	3	1.00	5	1.00	6	1.00
H2O + GIS	No	ST	PvIvE	25	0.72	17	0.71	14	0.71	14	0.93	17	0.59
H2O + GIS	No	ST	PvIwet	9	0.67	14	0.71	4	0.50	7	0.86	10	0.60
H2O + GIS	No	ST	PvNP	25	0.84	17	0.76	14	0.86	14	0.93	17	0.76
H2O + GIS	Yes	RF	EnotP	12	1.00	3	1.00	5	1.00	5	1.00	4	1.00
H2O + GIS	Yes	RF	EvIdry	14	0.71	3	1.00	10	0.60	7	0.71	7	0.71

H2O + GIS	Yes	RF	EvALI	25	0.84	17	1.00	14	0.71	14	0.86	17	0.88
H2O + GIS	Yes	RF	PnotE	5	1.00	5	1.00	3	1.00	5	1.00	6	1.00
H2O + GIS	Yes	RF	PvIvE	25	0.68	17	0.47	14	0.64	14	0.64	17	0.59
H2O + GIS	Yes	RF	PvIwet	9	0.67	14	0.36	4	0.75	7	0.57	10	0.50
H2O + GIS	Yes	RF	PvNP	25	0.84	17	0.47	14	0.93	14	0.79	17	0.71
H2O + GIS	Yes	ST	EnotP	12	0.75	3	1.00	5	1.00	5	1.00	4	1.00
H2O + GIS	Yes	ST	EvIdry	14	0.79	3	1.00	10	0.70	7	0.43	7	0.57
H2O + GIS	Yes	ST	EvALI	25	0.80	17	1.00	14	0.86	14	0.71	17	0.82
H2O + GIS	Yes	ST	PnotE	5	1.00	5	1.00	3	1.00	5	1.00	6	1.00
H2O + GIS	Yes	ST	PvIvE	25	0.68	17	0.65	14	0.71	14	0.64	17	0.71
H2O + GIS	Yes	ST	PvIwet	9	0.67	14	0.57	4	0.75	7	0.86	10	0.80
H2O + GIS	Yes	ST	PvNP	25	0.76	17	0.65	14	0.86	14	0.93	17	0.88

File S5. Focus-area studies

Focus area studies were conducted in two watersheds, one in California (i.e., the Santa Margarita River) and another in Arizona (i.e., the Hassayampa River). Each focus area study was led by practitioners with different backgrounds, but were likely to need to generate streamflow duration information as part of their job duties. The California focus area study was led by a private consultant with experience in wetland delineations and related jurisdictional matters, and the Arizona focus area study was led by an environmental scientist with the state regulatory agency. Each practitioner was provided with a day of training in the same protocols described in Supplement , after which they collected data from each reach during multiple repeated visits throughout the year.

Reaches within each focus area were located along a longitudinal gradient from headwaters to mainstems, without prior knowledge of flow duration; Stream Temperature, Intermittence and Conductivity loggers (STIC loggers, [36]) were installed at each watershed reach to enable their eventual classification. Data from these studies were evaluated using the beta SDAM AW, and results were presented to each practitioner. The practitioner then provided feedback on the resulting classifications, as well as on their experience using the protocol.

File S5.1. California focus-area study

Five reaches were selected in Murrieta Creek (a tributary of the Santa Margarita River in southern California), and one reach was selected in the Santa Margarita River just below the confluence. Each reach was visited on three occasions: the first in the peak of the dry season of 2019, the second a month after the end of the rainy season in 2020. The final visit occurred in the subsequent dry season in late summer of 2020. All six reaches were classified as *At least intermittent* at least once, and several were classified as perennial on one or more occasions. The practitioner conducting this focus area study determined that most classifications were correct, except for two intermittent reaches that were classified as perennial during the second visit, and one reach that was classified as *Need more information* on the first visit. The reach classified as *Need more information* was dominated by sandy substrate and is subject to frequent disturbance by off road vehicles. Thus, algal and invertebrate indicators were less able to persist into the dry season at this reach, compared to upstream reaches with more stable substrate and less active recreation. Hydrophytic plants were more robust to these disturbances and were evident at every reach-visit.

The practitioner believed that the focus area study provided new insight into his understanding of the watershed by highlighting the ability of biological indicators to integrate hydrologic information. Although he initially expected the upper-most reach in the study to be ephemeral based on its small watershed size and geomorphic characteristics, he thought that the presence of biological indicators was compelling evidence that it had intermittent flow duration.

The practitioner conducting this focus area study had a background in physical sciences and botany, but less experience with entomology. He felt that sampling and identifying aquatic invertebrates was a challenge, and he may have overlooked EPT taxa that were likely present at the lower two reaches, which are truly perennial. Deep water at those reaches—particularly during the second visit—may have further complicated sampling of aquatic invertebrates. He believed that adjustments we made to the protocol (specifically, dropping family-level identifications and replacing them with presence/absence of EPT taxa) would make the method more accessible and easier to use.

Variability in classifications and indicator measurements in the California focus area study. The total stream-length is approximately 20 km. BMI: Aquatic invertebrates. EPT: Ephemeroptera, Plecoptera, and Trichoptera. Refer to **Error! Reference source not found.** for information on indicator levels.

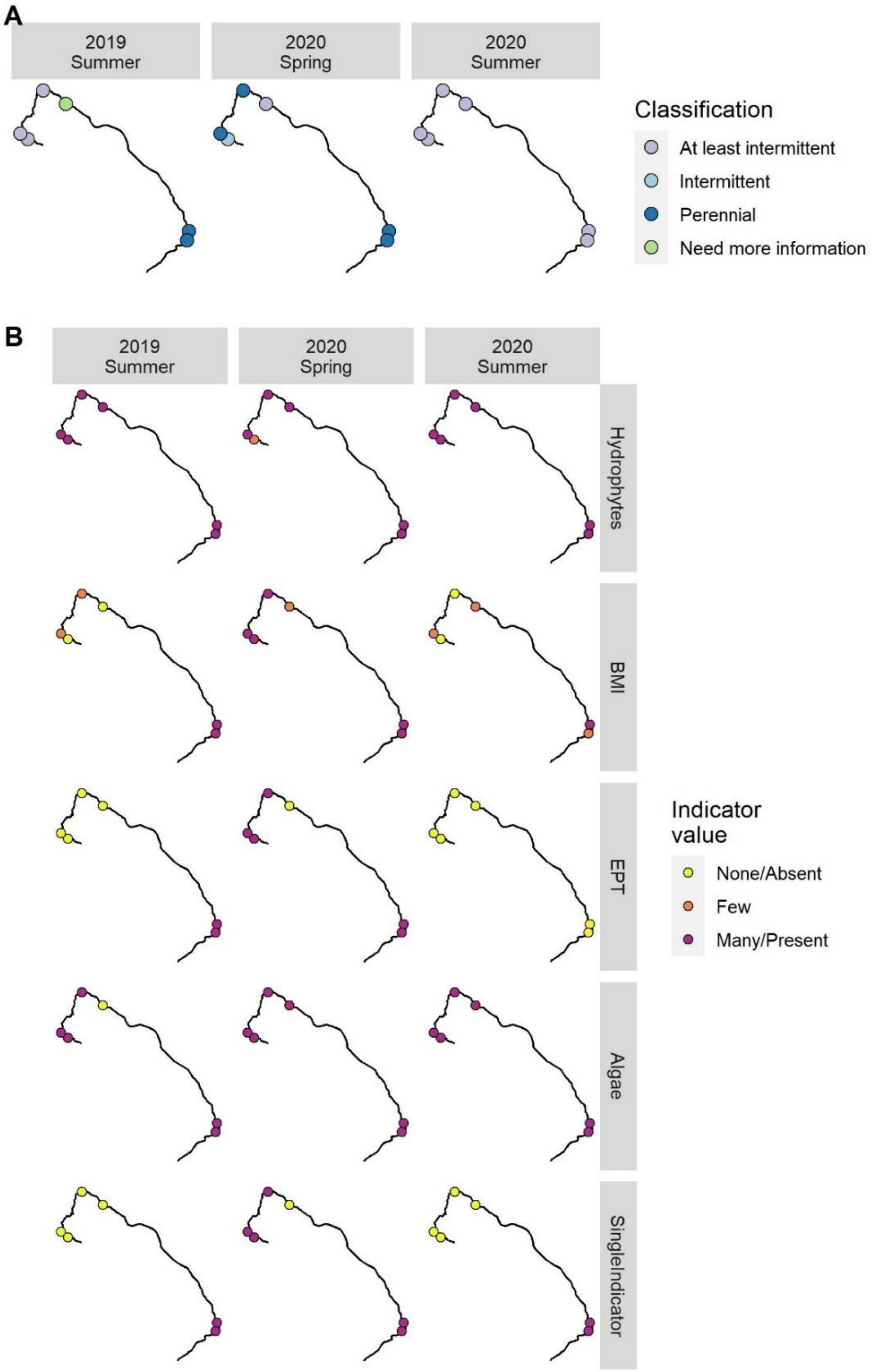

File S5.2. Arizona focus-area study

Ten reaches were selected along the Hassayampa River, a large river to the west of Phoenix. Reaches ranged from the headwaters in the Bradshaw Mountains to a few kilometers north of its confluence with the Gila River. Streamflow duration information was needed at these reaches for a number of pending management decisions related to the application of water quality standards and remediation of mining impacts. A total of 10 reaches were visited on at least two occasions between 2019 and 2021. Reaches in the upper part of the river were consistently classified as *Perennial* or *At least intermittent*, whereas classifications in the lowest portions were less consistent. Most of the variability was attributable to the aquatic invertebrate indicators, in contrast with algae and hydrophytic plant species. Classifications resulted in *Need more information* four times: twice at reaches where other assessments resulted in more conclusive classifications, and twice at the same reach.

The practitioner believed that the classifications produced by the SDAM were correct, although the frequency of *Need more information* classifications was higher than she expected. Therefore, guidance on interpreting results in those situations would be helpful. Although this practitioner was experienced with identifying aquatic invertebrates, she believed that field-based taxonomic identifications may be prone to errors and demand more training than she would be able to provide her staff.

Variability in classifications and indicator measurements in the Arizona focus area study. The total stream-length is approximately 100 km. BMI: Aquatic invertebrates. EPT: Ephemeroptera, Plecoptera, and Trichoptera. Refer to **Error! Reference source not found.** for information on indicator levels.

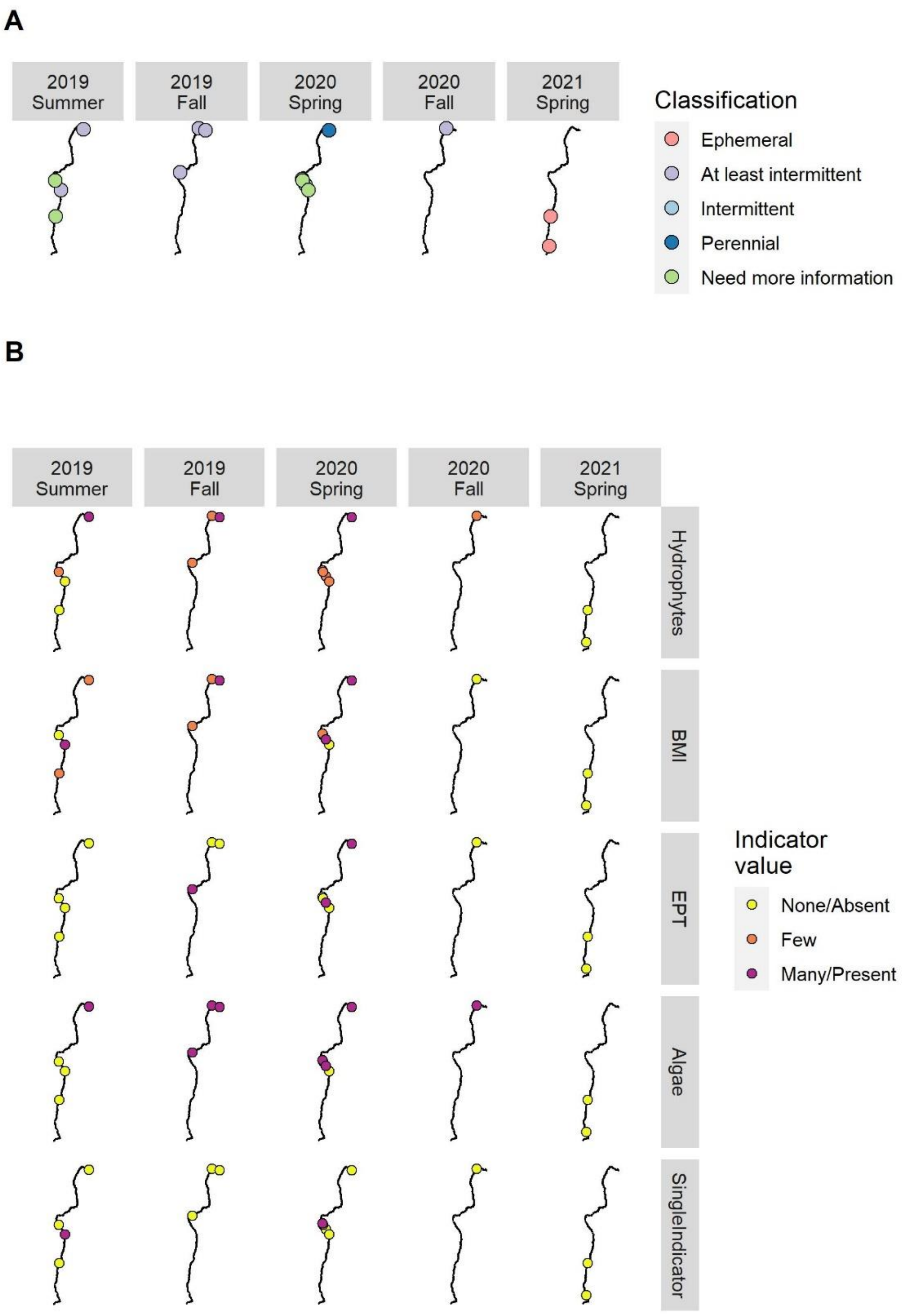
A
2019 Summer
2019 Fall
2020 Spring
2020 Fall
2021 Spring
Classification
Ephemeral
At least intermittent
Intermittent
Perennial
Need more information
B
2019 Summer
2019 Fall
2020 Spring
2020 Fall
2021 Spring
Hydrophytes
BMI
EPT
Algae
SingleIndicator
Indicator value
None/Absent
Few
Many/Present
